# Wildland–urban interface co-combustion of biomass, synthetic polymeric materials, and lithium-ion batteries generates a new class of ultrafine soot–metal–PAH hybrid particles

**DOI:** 10.3389/fpubh.2026.1768652

**Published:** 2026-03-03

**Authors:** Md Jalal Uddin Rumi, Yulin Wu, Md Jakir Hossain, Mazyar Etemadzadeh, Mengying Zhang, Todd A. Kingston, Rui Li, Guowen Song

**Affiliations:** 1 Department of Mechanical Engineering, Iowa State University, Ames, IA, United States; 2 Department of Apparel, Events, and Hospitality Management, Iowa State University, Ames, IA, United States

**Keywords:** exposure risk, metal–soot particle, polycyclic aromatic hydrocarbons, ultrafine emission, wildland–urban interface

## Abstract

Wildland–urban interface (WUI) fires increasingly involve the co-combustion of biomass with synthetic polymers such as polystyrene (PS) and lithium-ion batteries (LIBs); yet the resulting particulate emissions, including ultrafine particles (≤0.1 μm), remain insufficiently quantified and mechanistically unresolved. Here, we present a size- and chemistry-resolved analysis of particulate matter (PM) covering ultrafine particles (≤0.1 μm), fine particles (0.1–2.5 μm) and coarse particles (2.5–10 μm), trace elements, and polycyclic aromatic hydrocarbons (PAHs) emitted under controlled, near-source flaming conditions (50 kW/m^2^ radiant heat flux; 20.95% O₂) for four representative fuel combinations (Pine, Pine + PS, Pine + LIB, and Pine + PS + LIB). Pure pine combustion produced ultrafine-dominated emissions (~81% by number) with low PM mass (16 μg/m^3^), trace metals (0.41 μg/m^3^), and PAHs (13 ng/m^3^). In contrast, LIB and/or polymer involvement induced firm number–mass decoupling, shifting PM mass to the fine mode and increasing total PM up to 3.3-fold. Battery involvement led to a > 19-fold enrichment of particulate trace elements, dominated by nickel, lithium, phosphorus, cobalt, and aluminum, and to the formation of compact metal–soot hybrid particles during thermal runaway. PAHs increased concurrently, with preferential partitioning of carcinogenic high-molecular-weight species into ultrafine and fine particles. These results show that battery- and polymer-involved WUI fires generate a chemically distinct class of respirable particles enriched in toxic metals and PAHs that cannot be inferred from biomass combustion alone and are poorly captured by mass-based air-quality metrics, highlighting an emerging exposure risk for firefighters and nearby populations.

## Graphical abstract

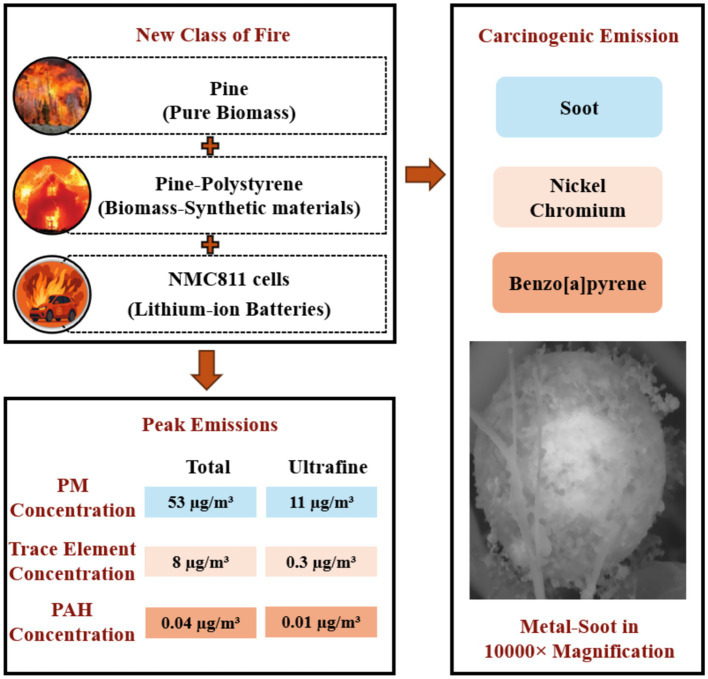

## 1 Introduction

Wildland and wildland–urban interface (WUI) fires are becoming increasingly frequent, larger, and longer-lasting due to the combined influences of climate change, fuel accumulation, and the continued expansion of housing into fire-prone regions (1, 2). Global assessments project that extreme wildfire events will rise by up to 14% by 2030, 30% by 2050, and 50% by the end of the century, making WUI communities and ecosystems particularly vulnerable (1). Recent events illustrate the scale and rapid escalation of these hazards: the 2023 Lahaina fire in Maui destroyed over 2,200 structures and caused 102 fatalities (3); the 2023 Canadian fires burned approximately 15 million hectares, the largest area in recorded history (4); and the January 2025 Eaton and Palisades fires near Los Angeles resulted in extensive structural loss, prolonged smoke exposure, and significant economic disruption (5). These cases underscore the urgent need to understand the evolving chemical nature and toxicity of WUI smoke.

From a public health standpoint, wildfire smoke is primarily evaluated through mass of PM_2.5_ (≤2.5 μm) and PM_10_ (≤10 μm), which forms the basis of air quality advisories and acute exposure guidelines while ultrafine particles/PM_0.1_ (≤0.1 μm) contributions remain insufficiently quantified and mechanistically unresolved (2, 6–10, 11). Significantly, PM chemical composition and toxicity vary strongly with particle size; the combined effects of size-resolved composition and ultrafine particles are critical drivers that are poorly captured (12, 13). A large epidemiological literature associates wildfire-derived PM₂.₅ with increased respiratory and cardiovascular morbidity, including asthma exacerbations, chronic obstructive pulmonary disease (COPD) flare-ups, pneumonia, ischemic heart disease, and cardiac arrhythmias, and with disproportionate impacts on children, older adults, and underserved populations (14–16). Meta-analyses indicate that a 1 μg/m^3^ increase in wildfire PM₂.₅ is associated with measurable rises in respiratory hospital admissions, and health effects can persist for weeks to months following major fire events (15, 16). However, this regulatory and scientific focus on mass-based PM₂.₅ and PM_10_ metrics inherently obscures the role of ultrafine particles a fraction capable of deep lung penetration, translocation into the bloodstream, and efficient transport of redox-active metals and organic toxicants, yet largely absent from wildfire monitoring and risk frameworks (8, 17, 18). As WUI fires intensify and the composition of fuels shifts, the PM₂.₅ and PM_10_ paradigm increasingly fails to capture the accurate toxicological profile of emerging smoke sources.

The chemical composition of smoke in WUI scenarios differs fundamentally from that of traditional biomass-only wildfires. The burning of vegetation now frequently co-occurs with synthetic polymer materials from the built environment (e.g., polystyrene insulation, plastics, foams) and with lithium-ion batteries (LIBs) embedded in electric vehicles (EVs) and consumer electronics (2, 8, 19, 20). Real-world events, such as the Lahaina and Los Angeles fires, demonstrate that mixed-fuel beds comprising wood, plastics, textiles, metals, and electronic components produce highly heterogeneous smoke plumes with complex physicochemical interactions. Despite this rapid shift in combustion, little is known about how mixed-fuel combustion alters particle formation pathways, toxic metal emissions, and carcinogenic organic compounds compared with biomass-only combustion.

This knowledge gap is especially critical, given the accelerating adoption of LIBs in EVs and consumer electronics within WUI zones (8, 21). Among different LIB cathode chemistries—including lithium cobalt oxide (LCO), lithium manganese oxide (LMO), lithium nickel manganese cobalt oxide (NMC), lithium nickel cobalt aluminum oxide (NCA), and lithium iron phosphate (LFP)—Ni-rich NMC variants, particularly NMC811, dominate high-performance applications due to their superior energy density and cost-effectiveness (22, 97). Nonetheless, elevated nickel (Ni) content compromises thermal and structural stability, heightening vulnerability to thermal runaway (TR) under different abusive conditions (23, 24).

TR in LIBs initiates a cascade of exothermic reactions, leading to uncontrolled temperature spikes and potential cell rupture [(25, 26, 98–100). TR can be initiated by mechanical (e.g., crushing), electrical (e.g., overcharging, external short circuit), or thermal (e.g., overheating) abuse. Under these different abuse conditions, TR typically commences with the decomposition of the solid electrolyte interphase (SEI), progressing to electrolyte solvent vaporization, cathode oxygen release, and inter-electrode reactions (24, 25). Importantly, fire is not an inherent outcome for all LIB TRs but rather depends on multiple interacting factors, including cathode chemistry, state of charge (SOC), abuse mode, environmental conditions, LIB design, and capacity, among others (27). In general, higher SOC (e.g., 50–100%) increases the likelihood and severity of TR by elevating stored chemical energy and accelerating exothermic decomposition reactions (27, 28). During these events, LIBs release metal-rich particulates (29–31).

Simultaneously, polystyrene (PS) thermally degrades into styrene monomers and aromatic oligomers that polymerize and aromatize into soot, enhancing yields of high-molecular-weight (HMW) polycyclic aromatic hydrocarbons (PAHs) relative to biomass combustion, which primarily generates lower-molecular-weight (LMW) PAHs (32–35, 101).

Under WUI-relevant conditions, the simultaneous release of metal-rich particles from LIB TR and PAH-rich soot from biomass–polymer combustion provides a mechanistically plausible pathway for enhanced particle mass loading and chemical complexity, as observed in the present study. Although prior studies have independently characterized LIB-derived metal emissions and PAH formation from biomass–plastic combustion, their combined effects on particle composition and size-resolved toxicant enrichment have not been systematically quantified. Transition metals and other trace elements associated with LIB cathode materials may further influence soot structure and surface chemistry by catalyzing carbon reorganization or stabilizing PAHs on particle surfaces, processes previously demonstrated in controlled metal–carbon systems (36, 37). These interactions suggest, but do not yet establish, that battery-involved WUI smoke may exhibit greater toxicological potency than biomass-only smoke.

Despite this mechanistic understanding, substantial research gaps persist. Most LIB TR studies focus on bulk PM₂.₅ and gaseous toxicants such as Hydrogen Fluoride (HF), Carbon Monoxide (CO), and volatile organic compounds (VOCs) while providing limited resolution of the specific hazards of ultrafine particles (8, 25, 38, 39). At the same time, WUI combustion studies rarely investigate realistic mixed-fuel packages that burn biomass, synthetic materials, and LIBs concurrently, despite such conditions being prevalent in real-world fire scenarios (2, 19, 20). Very few experiments offer size-resolved characterization of metals and PAHs under harmonized conditions, leaving significant uncertainties regarding how these toxicants partition across ultrafine particles (≤0.1 μm), fine particles (0.1–2.5 μm) and coarse particles (2.5–10 μm) or how hybrid metal–soot–PAH particles form and evolve in battery-involved WUI fires (2, 8). These gaps limit the accuracy of emission inventories, constrain exposure and dispersion modeling, and hinder the development of evidence-based protection strategies for firefighters and nearby communities.

Air-quality regulations compound this challenge. Existing standards emphasize PM₂.₅ and PM₁₀ mass while lacking metrics for PM_0.1,_ especially ultrafine number concentrations and compositionally enriched, metal-bearing particles, despite longstanding evidence of their heightened biological reactivity (10, 11, 17). This mismatch between emerging fire chemistry and regulatory indicators underscores the need for controlled scenario-specific studies that integrate physical and chemical particle characterization across size modes.

To address these gaps, the present study systematically investigates emissions from four fuel configurations representing an escalation from biomass alone to starting proximity of WUI conditions: pure biomass (Pine), biomass with NMC811 LIB (Pine + LIB), biomass with a representation of synthetic material (Pine + PS), and biomass with both synthetic material and NMC811 LIB (Pine + PS + LIB). Under a uniform 50 kW/m^2^ radiant heat flux and 20.95% oxygen (O₂), we measured real-time particle number concentration (PNC) across size distributions (0.01–10 μm) using a scanning mobility particle sizer (SMPS, NanoScan 3,910, TSI Inc.) and an optical particle sizer (OPS, 3330, TSI Inc.). Also, size-segregated particulate matter was collected (0.01–10 μm) via Dekati Low Pressure Impactor (DLPI+) cascade impactor for multi-element metal quantification via Inductively Coupled Plasma–Mass Spectrometry (ICP–MS), U.S. Environmental Protection Agency (EPA)-priority 16 PAH speciation via Gas Chromatography–Tandem Mass Spectrometry (GC–MS/MS), and microstructural analysis via Field-Emission Scanning Electron Microscopy coupled with Energy-Dispersive X-ray Spectroscopy (FE-SEM/EDS). We hypothesize that mixed-fuel combustion involving LIBs and PS will substantially alter emission profiles relative to biomass alone, producing ultrafine and fine particles enriched in transition metals and carcinogenic PAHs, with potential exceedances of short-term exposure benchmarks.

By integrating particle dynamics, size-resolved chemistry, and microstructural insights, this study aims to elucidate the formation of ultrafine metal–soot–PAH hybrids in battery-involved WUI fires and to provide exposure-relevant metrics for emission inventories, firefighter protection, and public health risk assessment.

## 2 Materials and methods

### 2.1 Selection and preparation of pine and polystyrene materials

To reflect the starting proximity of WUI fires where biomass, synthetic materials, and embedded LIBs burn concurrently, this study selected pine as pure biomass (the representative wildland fuel) and polystyrene (PS) as a starting proxy for common synthetic materials and furnishings present in North American WUI communities (2).

Both Pine and PS were processed into standardized geometries to ensure reproducible burning conditions. Bulk material was first cut into 100 × 100 mm pieces and then milled to a nominal particle size of 500 μm using a hammer mill (Schutte-Buffalo, USA). The resulting powders were compressed into cylindrical briquettes (100 mm diameter, 13 mm thickness, bulk density 1.0 g/cm^3^) using a hydraulic press (MTS, USA). This approach, adapted from prior WUI fuel investigations (40), minimized heterogeneity in packing, airflow, and heating rate compared with unprocessed boards.

All fuel discs were conditioned in an environmental chamber at 21 ± 2 °C and 65 ± 5% relative humidity for a minimum of 24 h prior to testing to reduce variability in moisture content, in line with established procedures for standardized combustion experiments (41, 42). Elemental composition (C, H, N, S, O) was determined using a CHNS/O analyzer (FlashSmart™, Thermo Scientific, USA); complete compositional data are provided in the Supplementary Section S.2.1 and Supplementary Table S.2.1.

### 2.2 Lithium-ion battery characteristics

Cylindrical 18,650-format LIBs (18 mm diameter × 65 mm length) with NMC cathode (LiNi₀.₈Mn₀.₁Co₀.₁) chemistry were selected due to their prevalence in EVs and consumer electronics (21, 22). Each NMC811 LIB had a nominal capacity of 2,500 mAh, a nominal voltage of 3.7 V, an upper cut-off voltage of 4.2 V, a lower cut-off of 2.5 V, a rated maximum continuous discharge current of 20 A, and a typical mass of ~43 g. NMC811 LIB was embedded along with pine and PS to prepare the fuel package (Section 2.4 Test matrices and experimental conditions), reflecting their widespread deployment in consumer electronics, EVs in residential and urban settings within WUI zones (2, 8).

Proprietary information on the internal electrolyte composition and electrode formulations was not disclosed in the manufacturer’s safety data sheet. Therefore, cathode chemistry was independently verified by EDS analysis of pristine electrode samples. The Ni: Mn: Co atomic ratio was approximately 7.8:1:1.4, consistent with an NMC811-type cathode (43).

### 2.3 Galvanostatic cycling and state-of-charge conditioning

To establish baseline electrochemical performance for accurate SOC determination, pristine batteries were subjected to three galvanostatic cycles at C/10 (0.25 A) between 2.7 and 4.2 V using a Neware BTS-5V12A testing system at room temperature. Charging durations were determined by each LIB’s measured capacity and then charged to achieve 50% SOC. The selected SOC is consistent with prior work linking TR severity and emissions to partial charge levels (23, 29, 30). The 100% SOC condition was omitted due to the risk of explosion from excessive gas generation in NMC811 LIB (102).

### 2.4 Test matrices and experimental conditions

Four fuel configurations were evaluated, as listed in Table 1, to represent emissions from wildland, battery-involved wildland, WUI mixed-fuel, and battery-involved WUI. This structured matrix ensures comparability across scenarios and enables direct evaluation of how batteries and synthetic materials modify particle formation, chemical composition, and toxicant emissions. All experiments were conducted under identical conditions (50 kW/m^2^; 20.95% O₂) for a fixed duration of 30 min to isolate the influence of fuel composition, combustion conditions on emission profiles. Smoke emission characterization (including particle number, mass, and size distribution) and trace element analysis by ICP-MS were performed in triplicate (n = 3) per fuel package to ensure statistical robustness (24), while PAH analysis by GC–MS/MS was conducted once (*n* = 1) per fuel package due to resource constraints.

**Table 1:** Fuel packages and thermal exposure conditions used in combustion experiments.

Sample ID	Fuel package representation	Fuel materials and weight percentile (%)	Weight (g)	Heat flux (kW/m^2^)	Oxygen (%)
Pine	Pure biomass (Wildland)	Pine 100 wt.%	Pine: 105 ± 1.50	50	20.95
Pine + LIB	LIB involved biomass (Hybrid wildland)	Pine (66 wt.%) + NMC811 LIB (34 wt.%)	Pine: 82 ± 1.53 NMC811: 43 ± 0.08	50	20.95
Pine + PS	Biomass with synthetic material (WUI mixed fuel)	Pine (95 wt.%) + PS (5 wt.%)	Pine: 100 ± 1.25 PS: 5 ± 0.25	50	20.95
Pine + PS + LIB	LIB involved biomass and synthetic material (Hybrid WUI)	Pine (61 wt.%) + PS (5 wt.%) + NMC811 LIB (34 wt.%)	Pine: 76 ± 1.52 PS: 6 ± 0.25 NMC811: 43 ± 0.03	50	20.95

### 2.5 Experimental fire simulation setup

**Figure 1:**
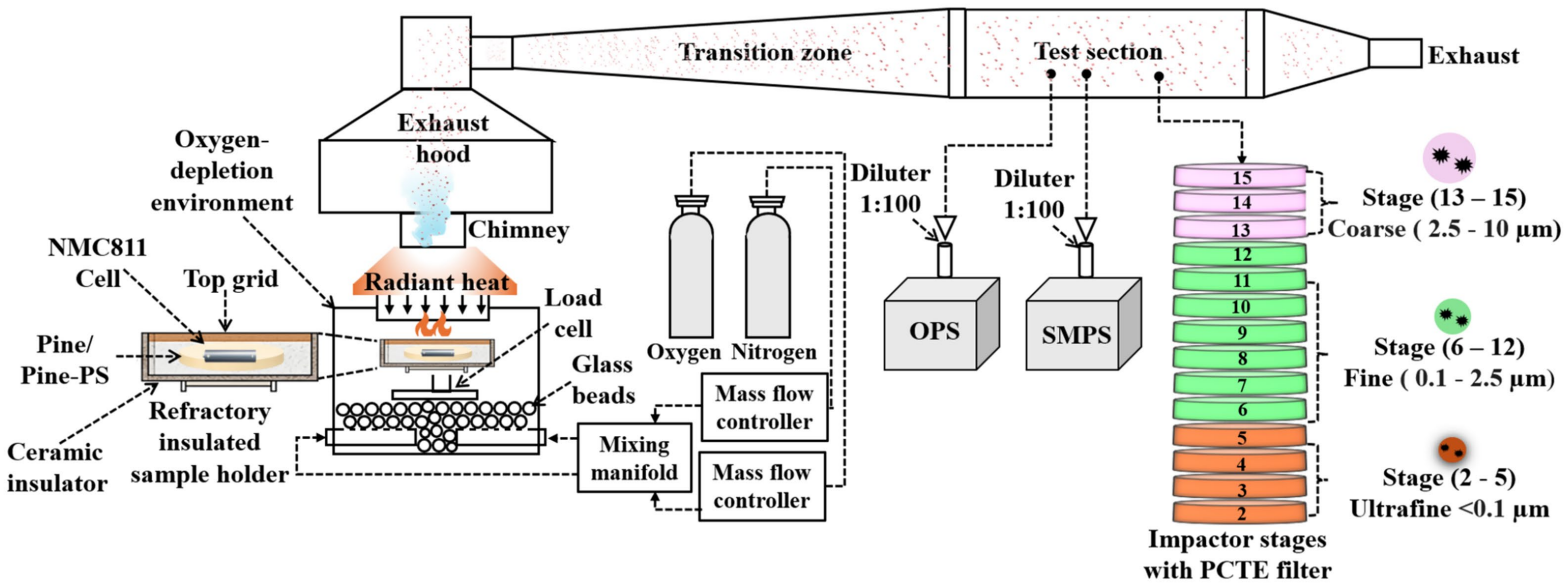
Schematic of the experimental test facility, including the combustion chamber, smoke tunnel, and instrumentation, replicating full-scale smoke exposure pathways during different fire scenarios.

The tests were carried out in a custom-built, atmosphere-controlled combustion chamber designed to meet ISO/TS 5660–5 specifications (44). The configuration was designed to simulate intense, localized heating conditions encountered during structural or compartment fires, while maintaining experimental control (Figure 1).

The fuel package was mounted in an ASTM E1354-compliant holder equipped with a stainless-steel mesh restraint to prevent fragment ejection during thermal runaway. Heating was provided by a conical radiant heater delivering the prescribed 50 kW/m^2^ heat flux (44).

**Figure 2:**
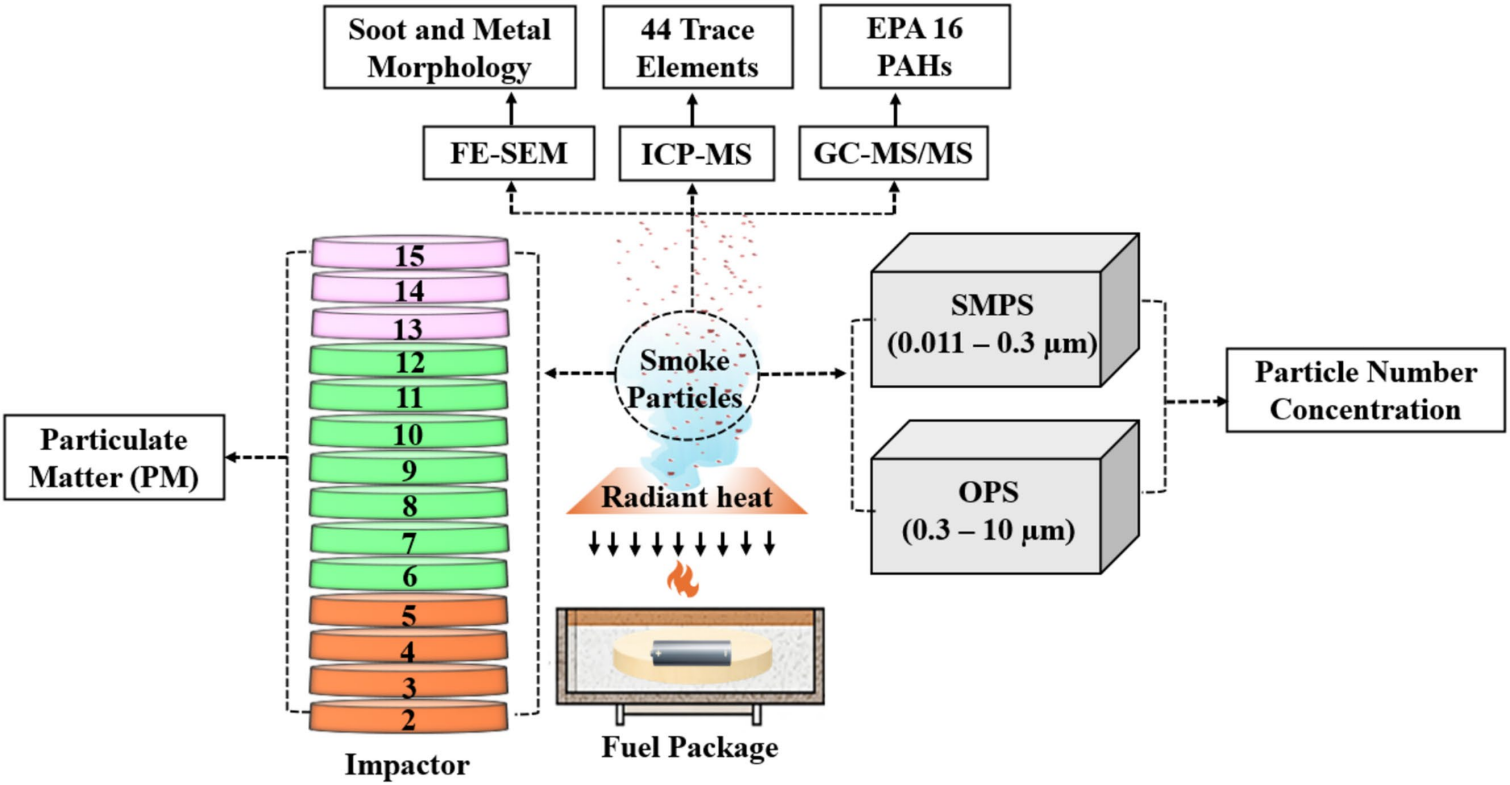
Multi-instrument workflow illustrates particle collection, size fractionation, and chemical/morphological characterization of emissions from different fire scenarios.

Gas distribution within the chamber was homogenized using a glass-bead dispersion layer, which promoted uniform temperature and oxidant delivery across the sample region (45, 46). Combustion effluents were conveyed through a 265 cm exhaust duct at a bulk flow rate of 42 L/s (test-section air velocity ~0.75 m/s), ensuring sufficient residence time and mixing before sampling (Figure 2). This configuration enabled integrated collection of particulates representative of near-field exposure conditions in enclosed or semi-enclosed environments. Although WUI fires predominantly occur in open air, the controlled lab-based combustion setup mimics the high-concentration, short-term exposures encountered by firefighters in proximity to WUI fires, where smoke particle levels are significantly elevated compared to far-field exposures affecting the general population (2, 8). This relevance stems from firefighters’ duties in semi-enclosed microenvironments during WUI incidents, enabling insights into acute health risks for first responders.

### 2.6 Particulate sampling, size segregation, and collection for chemical and morphological analyses

A multi-instrument sampling train was installed downstream of the combustion chamber (see Figure 1) to quantify particle number, mass, and their size distributions, as well as to perform morphological, trace elements, and chemical characterizations, as shown in Figure 2.

Each experimental fire was conducted for a fixed duration of 30 min. Real-time particle number and size distributions (0.011 to 10 μm) were captured using a SMPS (0.011 to 0.3 μm) and an OPS (0.3 to 10 μm), each coupled to precision diluters (1:100) to ensure concentrations remained within instrument-specific limits (47). This dilution for OPS and SMPS was applied throughout the whole 30-min experimental duration.

Particulates were collected using a DLPI+ cascade impactor directly from the raw exhaust stream without dilution at 10 L/min, covering 0.016–10 μm aerodynamic diameters and enabling size separation into ultrafine (stage 2 to 5), fine (stage 6 to 12), and coarse (stage 13 to 15) fractions consistent with previous ambient particulate studies (29, 30, 48, 49). For particulate mass, elemental, and chemical speciation, see Supplementary Section S.2.2 and Supplementary Table S.2.2 for the detailed methodology for particle collection using DLPI+. Particulates were deposited onto 0.1 μm polycarbonate track-etched (PCTE) membrane filters mounted on DLPI+ stages, which were subsequently used in ICP–MS to quantify 44 trace elements (see Supplementary Table S.3.3 for the list of 44 elements), and GC–MS/MS to determine concentrations of the 16 EPA-priority PAHs (see section 3.5).

For SEM–EDS characterization, particulates were collected with 37 mm quartz fiber filters (AQFA03700, MilliporeSigma, USA) using a sampling tube equipped with XAD-2 resins connected to Buck Elite pumps (A. P. Buck Inc., FL, USA), operated at 2 L/min (50). Quartz fiber filters were used as substrates for SEM–EDS characterization (51) to visualize metal-soot interactions at the microstructural level, rather than for quantitative analysis of elements such as carbon or other trace elements. Quantitative determination of trace elements was performed using ICP-MS, as detailed in section 2.9. A SEM-EDS on a blank quartz filter was also performed to report the microstructure and elements of the blank filter (refer to Supplementary Figure S.3.1). Sample preparation workflows for each analytical technique are described in sections 2.7–2.9.

### 2.7 Polycyclic aromatic hydrocarbon analysis by gas chromatography–tandem mass spectrometry

Size-resolved PAH emissions were quantified to characterize the organic toxicant profile of fuel packages replicating different fire scenarios (24, 29, 46, 48).

Sampling followed U.S. EPA Method TO-13A (103). Gas- and particle-phase PAHs were collected using XAD-2 cartridges (PUF–XAD-2–PUF, Restek, USA), 0.1 μm PCTE filters, and the DLPI+ impactor for size fractionation. The EPA 16 priority PAHs were analyzed using a GC–MS/MS system (Agilent 7,250 Q-TOF) equipped with a DB-5 ms column (30 m × 0.25 mm i.d., 0.25 μm film). The oven program ramped from 50 °C (held 1 min) to 320 °C at 10 °C/min, with a final hold of 5 min. Helium was used as the carrier gas at a flow rate of 1 mL/min, and ionization was performed at 70 eV (52).

Sample extraction was carried out using an automated Accelerated Solvent Extractor (ASE 350, Thermo Fisher, MA, USA), typically employing dichloromethane as the extraction solvent. Extracts were subsequently filtered and concentrated under a gentle nitrogen stream prior to injection (46). Method performance was verified through multi-point calibration (*R*^2^ > 0.99), with method detection limits ranging from 0.01 to 0.1 ng/m^3^ and quantification limits from 0.03 to 0.3 ng/m^3^. Surrogate standard recoveries between 60 and 120% were accepted (48). Additional method parameters, recoveries, and Quality Assurance/Quality Control metrics are presented in Supplementary Section S.2.3 and Supplementary Tables S.2.3, S.2.4.

### 2.8 Particle morphology and elemental mapping by field-emission scanning electron microscopy coupled with energy-dispersive X-ray spectroscopy

To elucidate the microstructural features and elemental composition of particulates, FE-SEM coupled with EDS was employed (29, 53). Particulates collected on a 37 mm quartz filter were examined using an FEI Quanta-FEG 250 microscope equipped with an Oxford AZtec EDS system.

Prior to imaging, samples were coated with a ~ 5 nm iridium layer using a Quorum Q150T ES sputter coater to improve electrical conductivity and reduce charging artifacts (54). Imaging was performed under low-vacuum conditions (0.1–0.3 Torr) at an accelerating voltage of 10 kV, a working distance of 10.8–11.3 mm, and a spot size of 3.0–4.0 μm (53). Both secondary and backscattered electron modes were used at magnifications ranging from 50 × to 50,000 × to resolve particle morphologies, aggregate structures, and surface features.

EDS mapping was applied to identify carbonaceous matrices and metal-bearing inclusions consistent with NMC LIB components and combustion residues. As is typical for SEM–EDS, detection of low-atomic-number elements (e.g., lithium) was limited by poor X-ray yield and matrix absorption, and quantification of trace elements in dense carbon matrices was constrained by background and overlapping peaks (54). These limitations motivated the complementary use of ICP–MS for high-sensitivity elemental analysis.

### 2.9 Trace element quantification by inductively coupled plasma–mass spectrometry

To provide a comprehensive and sensitive assessment of metal, metalloids, and nonmetal emissions, ICP–MS was employed for light elements and trace elements that are not reliably detected by SEM–EDS (29, 54). Analyses were conducted using a NexION 2000B ICP–MS (PerkinElmer, Waltham, MA, USA) targeting 44 elements, including key battery-relevant metals such as nickel (Ni), manganese (Mn), cobalt (Co), lithium (Li), aluminum (Al), phosphorus (P), and silicon (Si). See the Supplementary Supplementary Table S.3.3, for the complete 44-trace element list.

Particles were collected on 0.1 μm PCTE filters, chosen for their high collection efficiency and compatibility with acid digestion. Each filter was placed in a digestion tube containing 7 mL of an extraction mixture comprising 2.1% hydrogen peroxide (H₂O₂), 11% nitric acid (HNO₃), and 28.5% hydrochloric acid (HCl). Samples were digested on a hot block at 80 °C for 2 h to dissolve particulate-bound metals. After cooling, digests were quantitatively transferred and diluted to 25 mL with deionized water, then allowed to equilibrate for 12 h to ensure solution stability.

Aliquots (~2 mL) of the digested samples were introduced into the ICP–MS via a nebulizer, where the particulates were ionized in an argon plasma. The resulting ions were separated by mass-to-charge ratio and quantified against multi-element calibration standards. This method enabled the detection of low limits for a broad suite of elements, including transition metals and rare earths relevant to NMC battery chemistries, providing a robust complement to SEM–EDS for characterizing the metallic component of combustion particulates.

## 3 Results and discussion

### 3.1 Transformation of particle dynamics across fire scenarios

**Figure 3:**
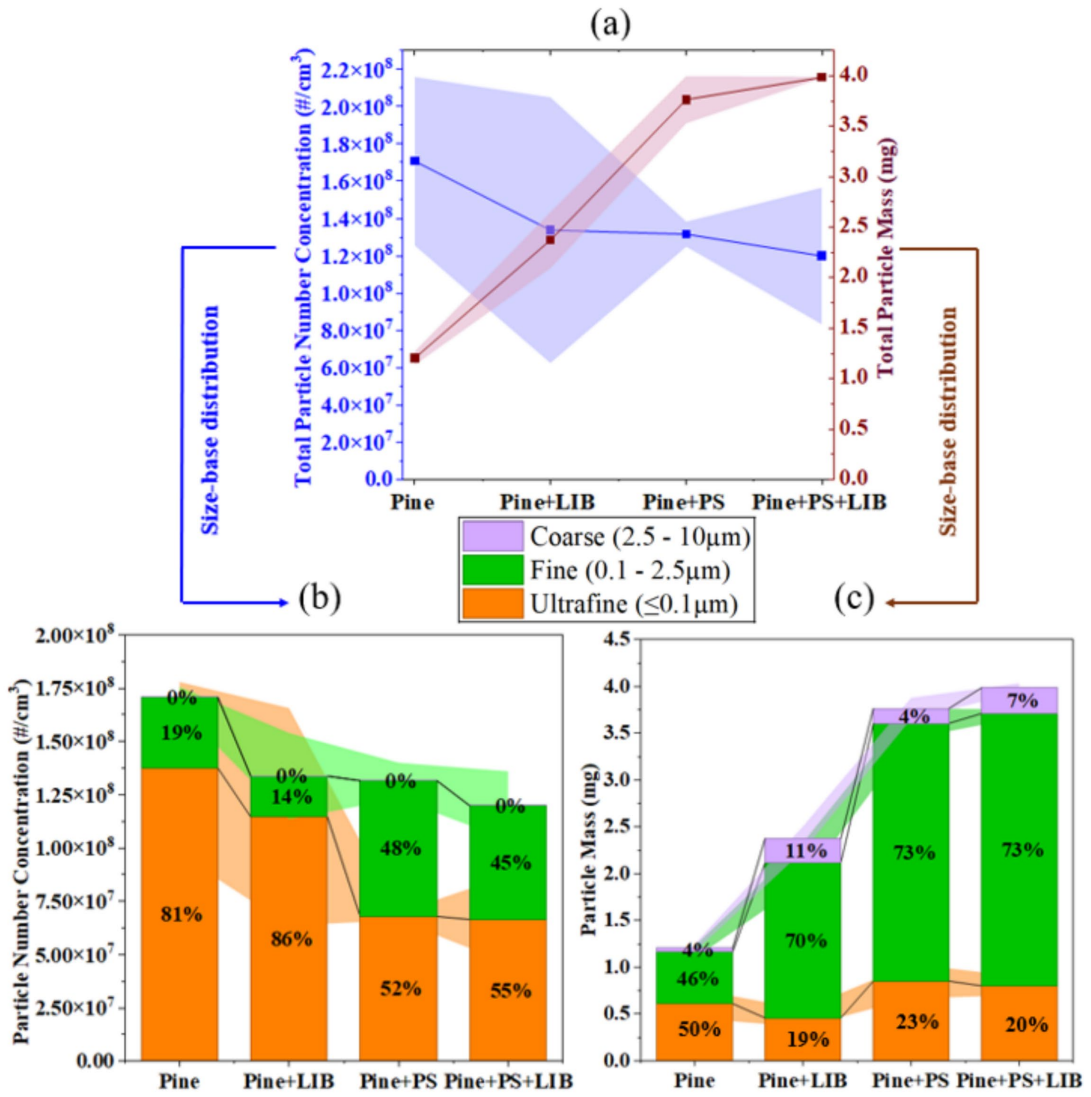
**(a)** Total particle number concentration (PNC) and total particle mass (PM); **(b)** Size-resolved PNC by bin (ultrafine particles ≤0.1 μm, fine 0.1–2.5 μm, coarse 2.5–10 μm); **(c)** Size-resolved PM by bin (ultrafine <0.1 μm, fine 0.1–2.5 μm, coarse 2.5–10 μm) in different fire scenarios.

Particle number concentration (PNC) and particulate mass (PM) across the four fuel configurations—Pine (100 wt.%), Pine + LIB (Pine 66 wt.% + LIB 34 wt.%), Pine + PS (Pine 95 wt.% + PS 5 wt.%), and Pine + PS + LIB (Pine 61 wt.% + PS 5 wt.% + LIB 34 wt.%)—reveal a systematic restructuring of the particulate from number-dominated ultrafine particles to mass-dominated fine and coarse aggregates (Figures 3a–c).

Pine combustion produced the highest total PNC 1.71 × 10^8^ ± 4.51 × 10^7^ #/cm^3^. The addition of an NMC811 as LIB and/or PS as synthetic material reduced total PNC to 1.34 × 10^8^ ± 7.12 × 10^7^ #/cm^3^ (−22%) for Pine + LIB, 1.32 × 10^8^ ± 6.71 × 10^6^ #/cm^3^ (−23%) for Pine + PS, and 1.20 × 10^8^ ± 3.66 × 10^7^ #/cm^3^ (−30%) for Pine+PS + LIB. In contrast, total collected PM increased from 1.20 ± 0.07 mg in Pine to 2.37 ± 0.28 mg (+98%) in Pine + LIB, 3.77 ± 0.23 mg (+214%) in Pine + PS, and 3.99 ± 0.01 mg (+233%) in Pine + PS + LIB. This inverse behavior of PNC and PM mass reflects accelerated coagulation and condensation driven by higher inorganic/organic loading from the synthetic material components, resulting in a marked shift from nucleation-dominated ultrafine particles toward accumulation- and coarse-mode aggregates (55–57).

Size-resolved contributions (Figures 3b,c) reinforce this transition. In Pine, ultrafine particles (≤0.1 μm), dominated number emissions, contributing ~81% of total PNC (1.38 × 10^8^ ± 4.04 × 10^7^ #/cm^3^), while fine particles contributed ~19% (3.32 × 10^7^ ± 6.86 × 10^6^ #/cm^3^) and coarse particles were negligible (<0.01%). By mass, Pine emissions were more evenly distributed, with ultrafine particles contributing ~50% (0.61 ± 0.16 mg), fine particles ~46% (0.55 ± 0.06 mg), and coarse particles ~4% (0.04 ± 0.04 mg). This is consistent with high-MCE flaming combustion of coniferous biomass, where abundant low-volatility VOC oxidation products drive rapid homogeneous nucleation and condensation, producing dominant ultrafine modes (number-weighted particle diameter, Dp ~ 0.06–0.07 μm) with limited inorganic seeding or coagulation sinks in fresh plumes (33, 58, 59).

In the Pine + LIB case, total PNC decreased, yet ultrafine particles remained dominant by number, contributing ~86% of PNC (1.15 × 10^8^ ± 5.10 × 10^7^ #/cm^3^), while fine particles accounted for ~14% (1.91 × 10^7^ ± 2.02 × 10^7^ #/cm^3^). However, ultrafine particles represented only approximately 19% of PM (0.46 ± 0.1 mg), whereas fine particles dominated the mass (~70%, 1.66 ± 0.22 mg), and coarse particles contributed approximately 11% (0.26 ± 0.13 mg). This pronounced number–mass decoupling reflects the coupled effects of NMC811 TR at 50% SOC and biomass combustion. During TR in NMC811 LIBs: electrolyte venting and SEI decomposition (SEI ~ 80–120 °C, anode–electrolyte ~120–180 °C, cathode ~180–250 °C) generate metal-rich vapors and fluorinated/organic gases that initially nucleate as <0.05 μm particles, when co-emitted with biomass-derived VOCs and soot, LIB-derived transition metals act as efficient condensation nuclei and catalytic surfaces, accelerating heterogeneous growth and Brownian coagulation into the accumulation mode (0.1–0.5 μm) within seconds to minutes (38, 60, 61). PNC of ~2 × 10^6^ #/cm^3^ and count median diameters shifting from ~0.03–0.08 μm (early venting) to 0.09–0.18 μm (peak TR) directly matches the observed redistribution (29–31).

In the Pine + PS case, the particle number distribution shifted substantially from ultrafine to fine modes. Ultrafine particles contributed ~52% of PNC (6.80 × 10^7^ ± 1.75 × 10^6^ #/cm^3^), representing a ~51% reduction relative to Pine ultrafine particles (1.38 × 10^8^ ± 4.04 × 10^7^ #/cm^3^), while fine particles increased to ~92% (6.36 × 10^7^ ± 8.45 × 10^6^ #/cm^3^) relative to Pine fine particles (3.32 × 10^7^ ± 6.86 × 10^6^ #/cm^3^). PM was strongly dominated by fine particles (~73%, 2.76 ± 0.16 mg), with smaller contributions from ultrafine particles (~23%, 0.85 ± 0.18 mg) and coarse particles (~4%, 0.16 ± 0.11 mg). This redistribution reflects aromatic-rich polystyrene pyrolysis (styrene oligomers and PAH precursors), which favors surface growth and coagulation on pre-existing nuclei and soot rather than new particle formation, producing chain-like aggregates predominantly in the 0.15–0.8 μm range even under well-ventilated conditions (62–64).

In the Pine + PS + LIB configuration, number contributions from ultrafine particles (~55%, 6.65 × 10^7^ ± 2.05 × 10^7^ #/cm^3^) and fine particles (~45%, 5.34 × 10^7^ ± 1.61 × 10^7^ #/cm^3^) were more balanced. PM reached its highest level (3.99 ± 0.01 mg), dominated by fine particles (~73%, 2.91 ± 0.04 mg), followed by ultrafine particles (~20%, 0.8 ± 0.1 mg) and coarse particles (~7%, 0.24 ± 0.09 mg). This distribution reflects synergistic effects of LIB vapor/metal nucleation bursts and PS-derived aromatic condensation promoting rapid growth into 0.1–1 μm metal–organic-soot hybrids closely mirroring real-world WUI plumes where biomass, plastics, and engineered materials (including EV batteries) co-burn (2, 61, 65).

### 3.2 Size-resolved particle number and mass distributions

**Figure 4:**
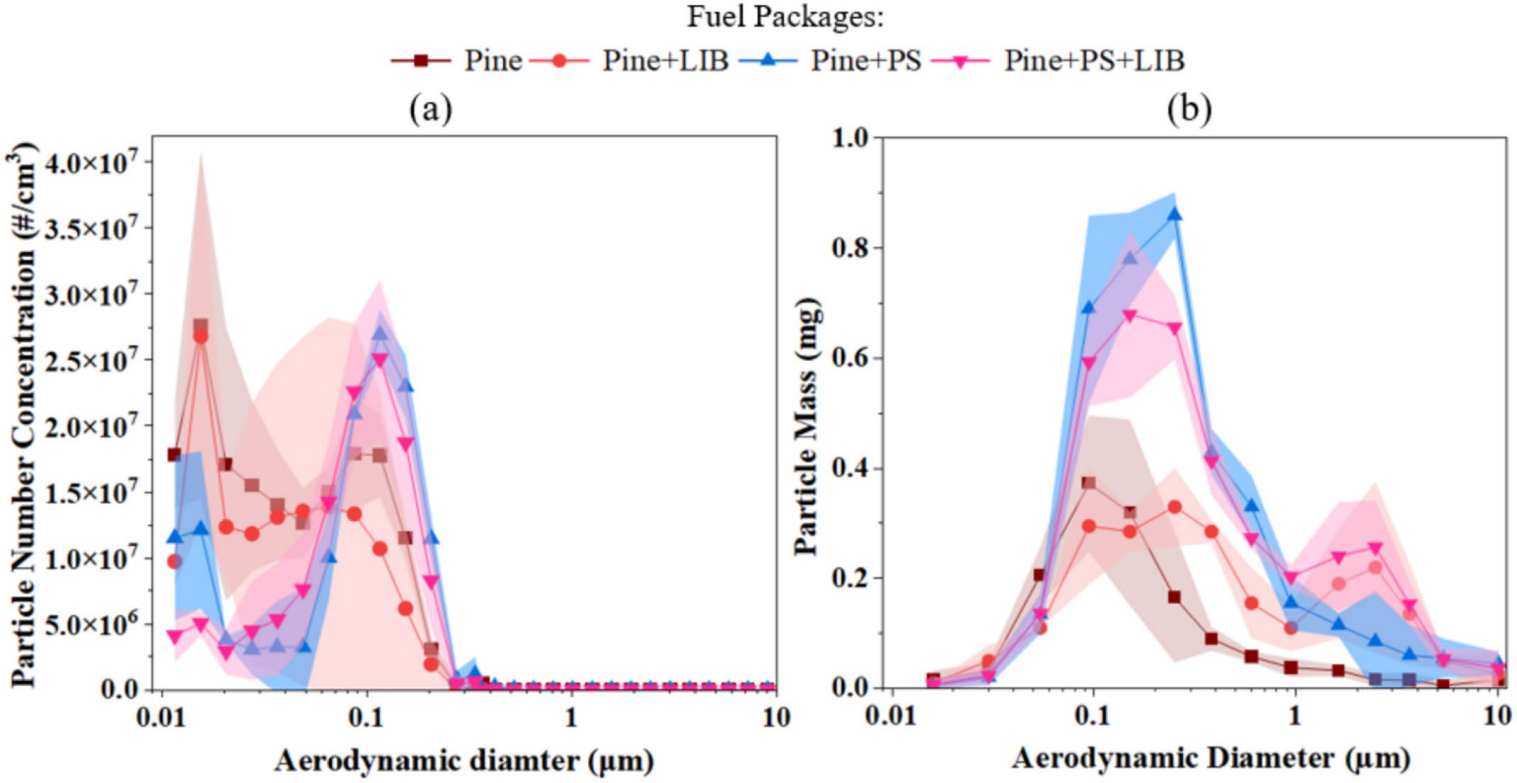
Particle size distribution by **(a)** Particle number concentration; **(b)** Particle mass in different fire scenarios.

Building on the aggregated PNC and PM behavior, the particle size distributions (PSDs) in Figure 4 illustrate how the four scenarios differ across the full aerodynamic range (0.011–10 μm for PNC and 0.016–10 μm for PM mass). Figure 4a shows that all cases exhibit firm nucleation-mode peaks (<0.1 μm) with exponentially decaying PNC toward larger sizes, while Figure 4b reveals how PM mass shifts from sub-0.1 μm to accumulation-mode diameters (0.1–1 μm) when LIB and PS are present (see Supplementary Table S.3.1 for PM and Supplementary Table S.3.2 for PNC detailed data across each size).

In the Pine case, PNC peaks at 2.76 × 10^7^ ± 1.31 × 10^7^ #/cm^3^ at 0.0154 μm, accounting for ~16% of total PNC, before rapidly declining to <10^3^ #/cm^3^ beyond ~1 μm. PM mass reaches its maximum at 0.37 ± 0.12 mg at 0.094 μm (~31% of the total PM), with substantial additional contributions between 0.05 and 0.25 μm (0.205–0.320 mg per stage, collectively accounting for ~40–45% of the total PM). This narrow, nucleation-skewed PSD is consistent with recent wildfire plume characterizations where pine and mixed conifers produce abundant sub-0.1 μm particles via rapid oxidation of biogenic volatiles (including terpenes) to low-volatility vapors, with limited growth into the accumulation mode in the absence of strong inorganic or anthropogenic influences (33, 66).

In the Pine + LIB case, PNC remains dominated by ultrafine particles, with a nucleation-mode peak of 2.68 × 10^7^ ± 1.42 × 10^7^ #/cm^3^ at 0.0154 μm, accounting for ~20% of total PNC, followed by a gradual decay toward larger diameters. In contrast to the particle number, PM mass is dominated by accumulation-mode particles, with a maximum of 0.33 ± 0.07 mg at 0.25 μm, collectively accounting for ~15–20% of total PM, while sub-0.1 μm particles contribute minimally. These features arise from TR-induced vapor bursts: fluoride–phosphate clusters from electrolyte decomposition nucleate at <0.05 μm and then grow via condensation of metal oxides (e.g., Co₃O₄, NiO), producing bimodal PSDs (0.02–0.08 μm and 0.2–0.5 μm) similar to those reported in controlled battery fire experiments (29–31).

In the Pine + PS case, PNC shifts toward larger sizes. The nucleation-mode peak occurs at 0.0866 μm with 2.09 × 10^7^ ± 5.60 × 10^5^ #/cm^3^, contributing ~16% of total PNC, a decrease of ~24% relative to the Pine only case at nucleation-mode peak from 2.76 × 10^7^ ± 1.31 × 10^7^#/cm^3^ at 0.0154 μm. In the Pine + PS case, accumulation-mode PNC peaks at 0.1155 μm with 2.69 × 10^7^ ± 1.89 × 10^6^ #/cm^3^, representing ~20% of total PNC, an increase of ~51% compared to Pine only case from 1.78 × 10^7^ ± 3.14 × 10^6^ #/cm^3^ at the same size bin. PM mass exhibits a pronounced accumulation-mode maximum at 0.15–0.25 μm, peaking with 0.86 ± 0.04 mg at 0.25 μm, corresponding to ~22% of total PM (3.77 ± 0.23 mg). This broadening and shift in modal diameter reflect PS pyrolysis and soot growth: styrene monomers and oligomers condense on existing cores, then evolve through cluster–cluster aggregation into fractal-like aggregates with mass-median diameters of 0.2–0.5 μm, as observed when synthetic materials (including polystyrene foams and vinyls) are co-combusted with biomass (32).

In the Pine + PS + LIB case, nucleation-mode PNC at 0.0154 μm decreases to 5.06 × 10^6^ ± 9.52 × 10^5^ #/cm^3^, ~4% of total PNC, representing a ~ 82% reduction relative to the Pine only case (2.76 × 10^7^ ± 1.31 × 10^7^ #/cm^3^) at the same size bin. Accumulation-mode PNC rises to 2.51 × 10^7^ ± 5.98 × 10^6^ #/cm^3^ at 0.1155 μm, ~21% of total PNC. PM mass peaks at 0.15 μm with 0.68 ± 0.15 mg, accounting for ~17% of total mass, with a minor coarse-mode contribution between 2.5–10 μm (0.03–0.15 mg, ~7% of total PM). LIB-derived trace elements provide abundant condensation nuclei and catalysts for soot ripening. At the same time, PS contributes aromatic precursors and carbonaceous mass, together producing a robust accumulation-mode population highly analogous to laboratory-measured WUI emission profiles where vegetation is co-burned with structural synthetic materials such as plastics, insulation foams under high heat flux (32).

### 3.3 Integrated PM, trace element, and PAH concentration across size fractions

**Figure 5:**
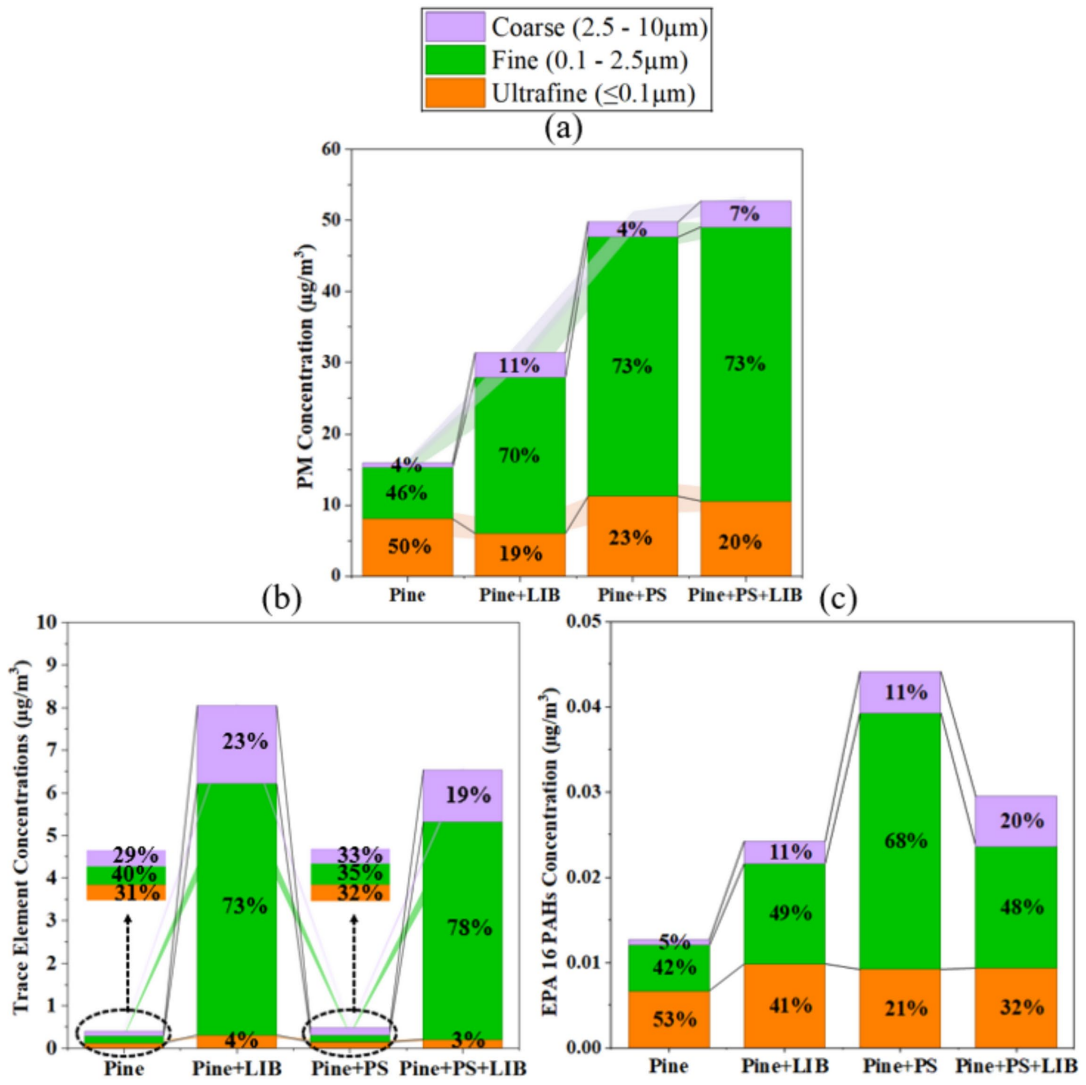
Total concentration of **(a)** PM; **(b)** metal; and **(c)** PAH in (μg/m^3^) under different fire scenarios.

The integrated PM, trace element, and PAH concentrations (Figure 5) quantify how the physical transformations described above translate into chemical loading across the ultrafine, fine, and coarse fractions.

Total PM concentration increases progressively across fire scenarios, from 16 ± 0.89 μg/m^3^ in Pine to 31.39 ± 3.65 μg/m^3^ in Pine + LIB, 49.80 ± 3.09 μg/m^3^ in Pine + PS, and 52.71 ± 0.09 μg/m^3^ in Pine + PS + LIB case (Figure 5A). In Pine, PM concentration fractions were dominated by ultrafine particles (8.07 ± 2.06 μg/m^3^, 50% of total), followed by fine fraction (7.28 ± 0.85 μg/m^3^, 46%) and coarse fraction (0.58 ± 0.55 μg/m^3^, 4%). NMC811 LIB incorporation redistributed PM concentration toward the fine fraction, with ultrafine particles decreasing to 6.04 ± 1.27 μg/m^3^ (19%), fine fraction increasing to 21.91 ± 2.87 μg/m^3^ (70%), and coarse fraction at 3.44 ± 1.73 μg/m^3^ (11%).

Addition of synthetic material PS with Pine produced 11.24 ± 2.43 μg/m^3^ ultrafine particles (23%), 36.44 ± 2.15 μg/m^3^ fine (73%), and 2.12 ± 1.50 μg/m^3^ coarse (4%), while the Pine + PS + LIB exhibited 10.58 ± 1.31 μg/m^3^ ultrafine particles (20%), 38.49 ± 0.56 μg/m^3^ fine (73%), and 3.64 ± 0.65 μg/m^3^ coarse (7%). These shifts confirm that synthetic PS material converts ultrafine biomass-derived particles into fine-mode-dominated particulates with larger aerodynamic diameters. Laboratory pine/conifer combustion studies typically report PM₂.₅ concentrations of 10–30 μg/m^3^ under flaming conditions (67) and wildfire PM_2.5_ exceeds 15 μg/m^3^ for at least two or three consecutive days from fire (68, 69), whereas biomass–synthetic material or WUI mixtures yield much higher due to enhanced soot and secondary particulate formation (32).

Mechanistically, terpenoid volatilization in Pine drives nucleation of ultrafine-dominated PM concentration (8.07 ± 2.06 μg/m^3^, 50% of total), whereas NMC811 LIB TR in Pine + PS moves concentration into the fine fraction (21.91 ± 2.87 μg/m^3^, +201% vs. Pine) via coagulation and condensation of electrolyte-derived vapors (including phosphates and carbonaceous fragments), producing fine-dominated distributions (29–31). Synthetic material-PS pyrolysis in Pine+PS further increases the fine fraction to 36.44 ± 2.15 μg/m^3^ (+400% vs. Pine) through cyclodehydrogenation and aggregation of aromatics into 0.1 μm aggregates (70, 71). In the Pine + PS + LIB case, fine fraction reaches 38.49 ± 0.56 μg/m^3^ (+428% vs. Pine), reflecting metal–hydrocarbon interactions and rapid accretion of ultrafine nuclei into the accumulation mode, consistent with enhanced soot yields and partial catalytic oxidation in mixed synthetic material-vegetation burns (32, 72).

Total trace element concentration (Figure 5b) increased markedly in scenarios involving the NMC811 LIB. Total trace element concentration rose from 0.41 ± 0.05 μg/m^3^ in Pine to 8.05 ± 0.39 μg/m^3^ in Pine + LIB, remained low in Pine + PS without NMC811 LIB (0.48 ± 0.05 μg/m^3^), and reached 6.54 ± 0.39 μg/m^3^ in Pine + PS + LIB case. In Pine, metals were relatively low across all size fractions (ultrafine 0.13 ± 0.01 μg/m^3^, 31%; fine 0.16 ± 0.02 μg/m^3^, 40%; coarse 0.12 ± 0.01 μg/m^3^, 29%). Incorporation of NMC811 LIB strongly enhanced fine-mode dominance: in Pine + LIB (ultrafine 0.32 ± 0.03 μg/m^3^, 4%; fine 5.90 ± 0.28 μg/m^3^, 73%; coarse 1.82 ± 0.08 μg/m^3^, 23%), and in Pine + PS + LIB (ultrafine 0.22 ± 0.03 μg/m^3^, 3%; fine 5.10 ± 0.28 μg/m^3^, 78%; coarse 1.23 ± 0.08 μg/m^3^, 19%). These increases are consistent with the contribution of trace elements released from NMC811 LIBs during TR, which act as condensation nuclei and favor the formation of fine accumulation-mode particles. These values are consistent with battery-fire studies reporting 5–15 μg/m^3^ total elements (predominantly Ni, Co, Mn, Al, Cu, Fe as oxides or fluorides) with >70–90% in the fine/accumulation mode (29–31, 72). Detailed concentrations and standard deviations for all 44 trace elements from triplicate tests are provided in Supplementary Tables S.3.3, S.3.4. Also, dominant battery-derived elements and their source attribution are provided in Section 3.4.

Under the fixed experimental conditions (50 kW/m^2^ heat flux; 20.95% O₂) employed in this study, total Σ16 EPA PAH concentrations increased from Pine (0.01 μg/m^3^) to Pine + LIB (0.02 μg/m^3^), Pine + PS (0.04 μg/m^3^), and Pine + PS + LIB (0.03 μg/m^3^) (Figure 5c). In the pure Pine case, PAHs were dominated by the ultrafine fraction (53%), followed by the fine (42%) and coarse (5%) fractions, consistent with biomass-derived LMW PAHs condensing primarily onto sub-0.1 μm particles. Incorporation of the NMC811 LIB with Pine redistributed PAHs toward larger sizes, reducing the ultrafine contribution to 41% while increasing the fine fraction to 49% and coarse fraction to 11%, indicating enhanced condensation and growth of PAHs under LIB-driven high-temperature conditions.

The Pine + PS scenario produced the highest Σ16 EPA PAH burden. It exhibited strong fine-mode dominance (68%) and a substantially reduced ultrafine contribution (21%), reflecting the efficient formation and growth of aromatic species during synthetic polymer pyrolysis. In the Pine + PS + LIB case, Σ16 EPA PAH remained elevated relative to Pine, with a more balanced size distribution (ultrafine 32%, fine 48%, coarse 20%), suggesting concurrent PAH formation, metal-assisted condensation, and oxidative fragmentation under extreme mixed-fuel combustion. These results represent a single-condition, exploratory assessment; recent work demonstrates that variations in burn environment alone can drive PAH emission variability of up to ~77%, comparable to or exceeding fuel-type effects (73). Particles ejected from LIB fires can contain PAHs (29), as can those from biomass combustion (70, 74), and PS also serves as a source of PAHs (75). Detailed congener-specific and size-resolved discussion of PAH formation mechanisms and carcinogenicity is provided in Section 3.5.

### 3.4 Dominant battery-derived elements and source attribution

**Figure 6:**
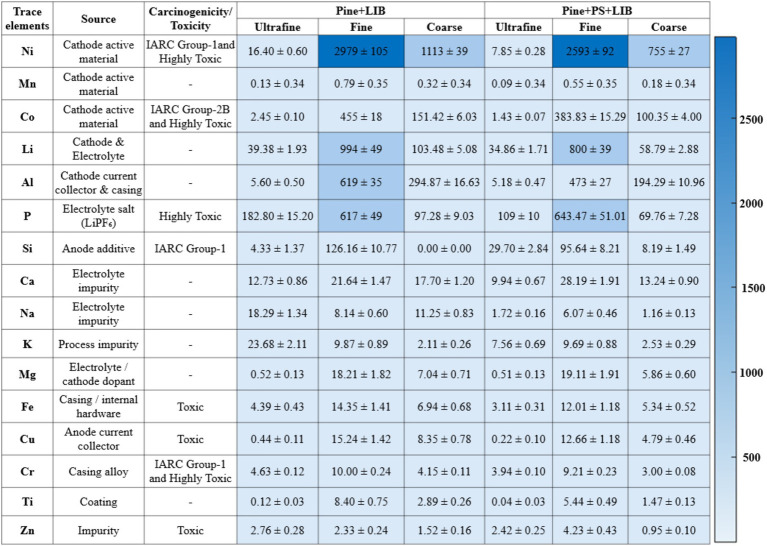
Dominant trace elements carcinogenicity and their concentration (ng/m^3^) in different fire scenarios.

Incorporation of an NMC811 LIB in Pine and Pine + PS markedly increased concentration and altered the distribution of particulate trace elements relative to Pine combustion alone (Figure 5b). Total trace-element concentration rose from 0.41 ± 0.05 μg/m^3^ in Pine to 8.05 ± 0.39 μg/m^3^ in Pine + LIB and 6.54 ± 0.39 μg/m^3^ in Pine + PS + LIB, whereas Pine + PS without battery involvement remained low (0.48 ± 0.05 μg/m^3^). These values represent time-integrated averages from triplicate experiments; individual element concentrations resolved by size fraction are reported in Figure 6 (in ng/m^3^ for individual elements to facilitate more precise visualization of lower-concentration elements without excessive decimal places). Size-resolved fractional analysis of all 44 trace elements in Supplementary Tables S.3.3–S.3.5 provides further quantitative support for battery-dominated source attribution in Pine + LIB and Pine + PS + LIB scenarios.

The primary battery-derived and minor trace elements were Ni, Mn, Co, Li, Al, P, and Si. In Pine + LIB, the ultrafine fraction was chemically distinct and strongly enriched in electrolyte-derived P (182.8 ± 15.2 ng/m^3^, 57.14% of ultrafine trace-element mass), followed by smaller contributions from Ni (16.4 ± 0.6 ng/m^3^, 5.13%) and Al (5.6 ± 0.5 ng/m^3^, 1.7%). In contrast, the fine fraction was overwhelmingly dominated by cathode-derived Ni (2,979 ± 105 ng/m^3^, 50.5%), with substantial contributions from phosphorus (617 ± 49 ng/m^3^, 10.45%), Li (994 ± 49 ng/m^3^, 16.84%), Al (619 ± 35 ng/m^3^, 10.5%), and Co (455 ± 18 ng/m^3^, 7.7%), confirming that the majority of battery-associated trace elements reside in the accumulation mode. A similar pattern persisted in the coarse fraction, where Ni alone accounted for 61.0% of trace-element mass (1,113 ± 39 ng/m^3^), indicating physical ejection and fragmentation of cathode materials (43, 76–78).

In the Pine + PS + LIB scenario, PS co-combustion did not alter the elemental hierarchy but modestly redistributed fractional contributions across size modes. Ni remained dominant in the fine (2,593 ± 92 ng/m^3^, 50.9%) and coarse (755 ± 27 ng/m^3^, 61.6%) fractions, while P continued to dominate the ultrafine fraction (109 ± 10 ng/m^3^, 50%), emphasizing that battery chemistry—not polymer co-combustion—governs trace-element identity (32, 43, 72, 78). Li (800 ± 39 ng/m^3^, 16%) and Al (473 ± 27 ng/m^3^, 9.3%) in the fine fraction reflect the combined effects of electrolyte decomposition and current-collector fragmentation. Mn was consistently detected in all fractions but contributed minimally (~0.07% of the total concentration; e.g., 1.24 ± 1.03 ng/m^3^ in Pine + LIB, the highest among all cases). The mechanisms governing Mn aerosolization remain poorly understood, underscoring the need for future investigations.

Across battery-containing scenarios, trace elements were strongly enriched in the fine fraction, accounting for ~73% (Pine + LIB) and ~78% (Pine + PS + LIB) of total trace-element concentration. Ultrafine particles contributed only ~3–4% of total trace element concentration but exhibited a chemically distinct signature dominated by electrolyte-derived P species. In Pine + LIB, P in the ultrafine fraction reached 182.8 ± 15.2 ng/m^3^ (~57% of ultrafine trace-element mass), with a comparable enrichment (~50%) in Pine + PS + LIB, consistent with thermal decomposition of LiPF₆-based electrolytes and nucleation of phosphate- and fluoride-containing species (29, 31, 43, 78).

The size-resolved trace-element distributions reported here provide the quantitative basis for the metal-anchored soot morphologies observed in Section 3.6 and inform the subsequent toxicological discussion (Sections 3.7–3.8).

### 3.5 PAH profiles and carcinogenicity across fire scenarios

**Figure 7:**
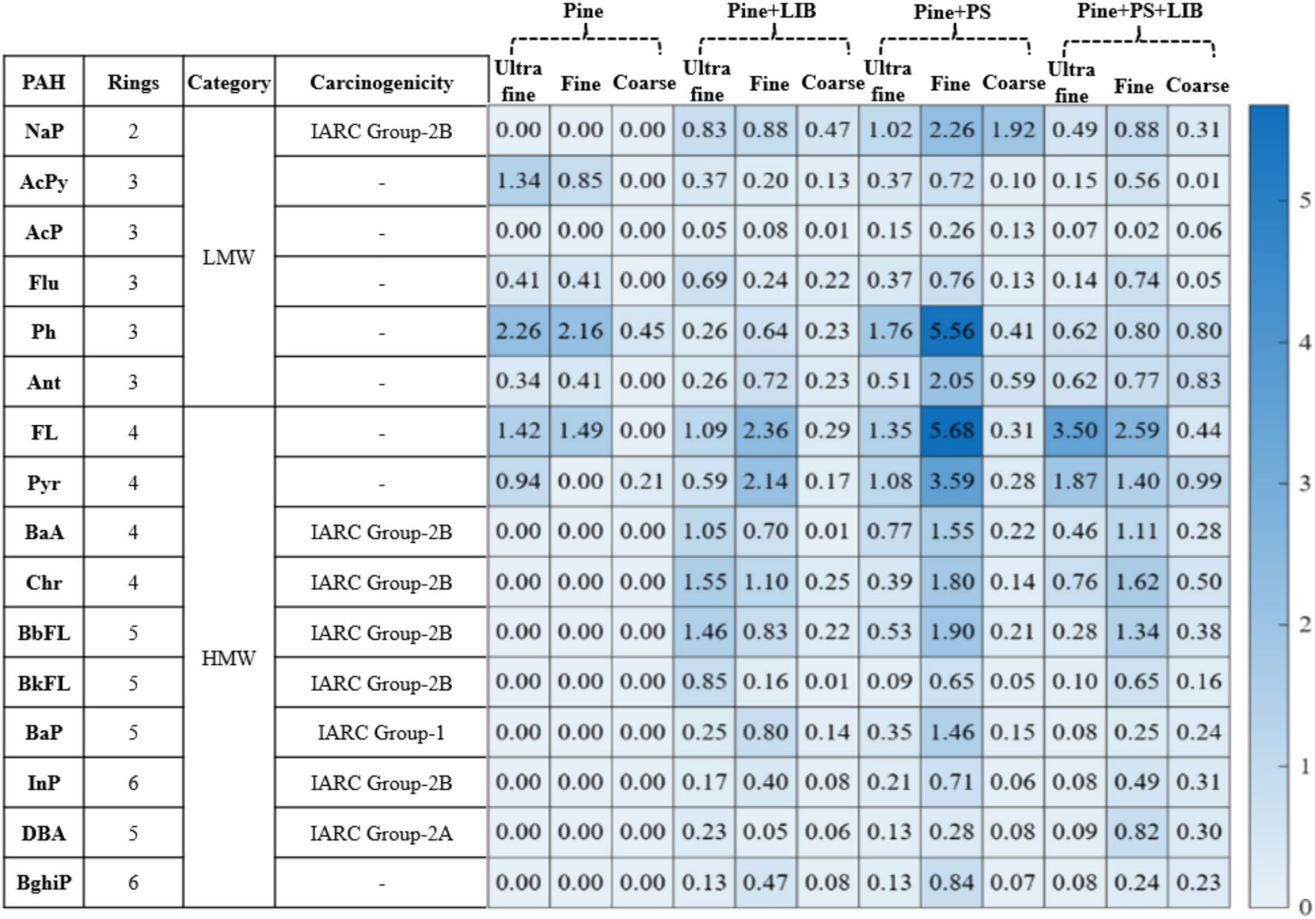
PAH carcinogenicity and their concentration (ng/m^3^) in different fire scenarios.

The size-resolved concentrations of the 16 U.S. EPA priority PAHs across the four experimental fire scenarios are illustrated in Figure 7 and listed in Supplementary Table S.3.6, with values reported in ng/m^3^ to facilitate visualization of both low- and high-abundance compounds in the ultrafine, fine, and coarse fractions. Percentage contributions of individual PAHs to the total summed PAH (ΣPAH) for each particle mode are also provided in Supplementary Table S.3.7.

In the Pine only scenario, ΣPAH reached 12.69 ng/m^3^, with emissions dominated by LMW (2–3 ring) PAHs. Phenanthrene (Ph) was the most abundant compound (2.26 ng/m^3^ in ultrafine, 2.16 ng/m^3^ in fine, and 0.45 ng/m^3^ in coarse particles), contributing 33.7–68.5% of ΣPAH depending on particle size, followed by fluoranthene (FL; 1.42–1.49 ng/m^3^, 21.1–28.1%) and pyrene (Pyr; up to 0.94 ng/m^3^, 14.0–31.5%). Ultrafine particles accounted for 53% of total ΣPAH, and no HMW (5–6 ring) PAHs such as chrysene (Chr), benzo[a]pyrene (BaP), or indeno[1,2,3-cd]pyrene (InP) were detected. This PAH profile is characteristic of well-ventilated softwood combustion, which preferentially forms LMW PAHs while suppressing HMW species (34, 79–81).

The addition of an NMC811 LIB in 50% SOC, along with Pine, elevated ΣPAH to 24.23 ng/m^3^, representing an approximate 2-fold increase compared to the Pine case. Ultrafine particles served as the second reservoir for total ΣPAH (~41%) after fine particles served as the primary reservoir for total ΣPAH (~49%). Ultrafine fraction exhibited enrichment with HMW, International Agency for Research on Cancer (IARC)-classified carcinogenic PAHs, such as chrysene (Chr, 1.55 ng/m^3^, 15.7% of mode-specific ΣPAH), benzo[b]fluoranthene (BbFL, 1.46 ng/m^3^, 14.8%), and benzo[a]pyrene (BaP, 0.25 ng/m^3^, 2.57%). Notably, LMW PAHs in Pine + LIB case showed reduced fractional contributions compared to the Pine case, indicative of enhanced aromatization pathways catalyzed by metal-containing particles; however, it is important to clarify that not all LIB TR result in fires, though in this experiment, the 50% SOC condition of NMC811 led to TR and fire (27, 29, 30, 76). Previous studies have reported metal-soot hybrid particles enriched with carcinogenic metals (e.g., nickel, cobalt) from standalone LIB fires (29, 76, 77). Particles ejected from LIB fires can contain PAHs (29), as can particles from biomass combustion (70, 74).

Incorporating 5 wt.% PS, along with Pine for the Pine + PS case, further increased ΣPAH to 44.12 ng/m^3^, a roughly 3.5-fold rise compared to the pine baseline and the highest among all scenarios. Both ultrafine and fine-mode PAHs showed substantial HMW PAH enhancements, with fine-mode PAHs accounting for 68% of total ΣPAH. Ultrafine particles accounted for 21% of total ΣPAH, including detectable HMW PAHs such as benzo[a]anthracene (BaA), Chr, BbFL, and BaP at levels ranging from 0.39 to 1.35 ng/m^3^. This elevation is consistent with the literature on styrene polymer combustion, which promotes HMW PAH production via depolymerization and oligomerization at 400–700 °C, unlike lignocellulosic fuel profiles [(16, 55, 81, 104, 105).

When NMC811 LIB was co-combusted with both Pine and PS (Pine+PS + LIB), ΣPAH reached 29.51 ng/m^3^, approximately 2.3 times higher than the pine baseline but lower than the Pine+PS case. Fine particles remained the dominant PAH reservoir (14.27 ng/m^3^, ~48%), while ultrafine particles contributed a substantial fraction (9.36 ng/m^3^, ~32%). The ultrafine mode exhibited the most substantial relative enrichment of carcinogenic HMW PAHs, including Chr (0.76 ng/m^3^, 8.08%). Fine-mode PAHs were dominated by fluoranthene, pyrene, and phenanthrene, together contributing approximately 30% of fine-mode ΣPAH. In comparison, the coarse fraction carried a comparatively minor PAH load (20%), indicating limited partitioning onto larger particles.

Across all scenarios, ΣPAH followed the progression of Pine (13 ng/m^3^) < Pine + LIB (24.2 ng/m^3^) < Pine + PS + LIB (29.5 ng/m^3^) < Pine + PS (44.1 ng/m^3^), with ultrafine particle enrichment of HMW PAHs peaking in the Pine + PS + LIB case due to the combined effects of LIB metals and synthetic fuel pyrolysis. Fine particles consistently dominated total ΣPAH, reflecting aerosol aggregation and growth. Töpperwien et al. (73) reported up to 77% variability in PAH phenanthrene/anthracene emissions, as these are highly dependent on combustion conditions, including temperature, oxygen availability, fuel moisture content, and flaming versus smoldering phases. While the PAH analysis in this study was limited to a single test configuration, future research should explore variations in temperature, oxygen availability, fuel moisture content, and flaming versus smoldering phases to quantify PAH variability better and enhance predictive models for real-world wildfire scenarios.

### 3.6 Metal–soot morphologies and implications for particle evolution

**Figure 8:**
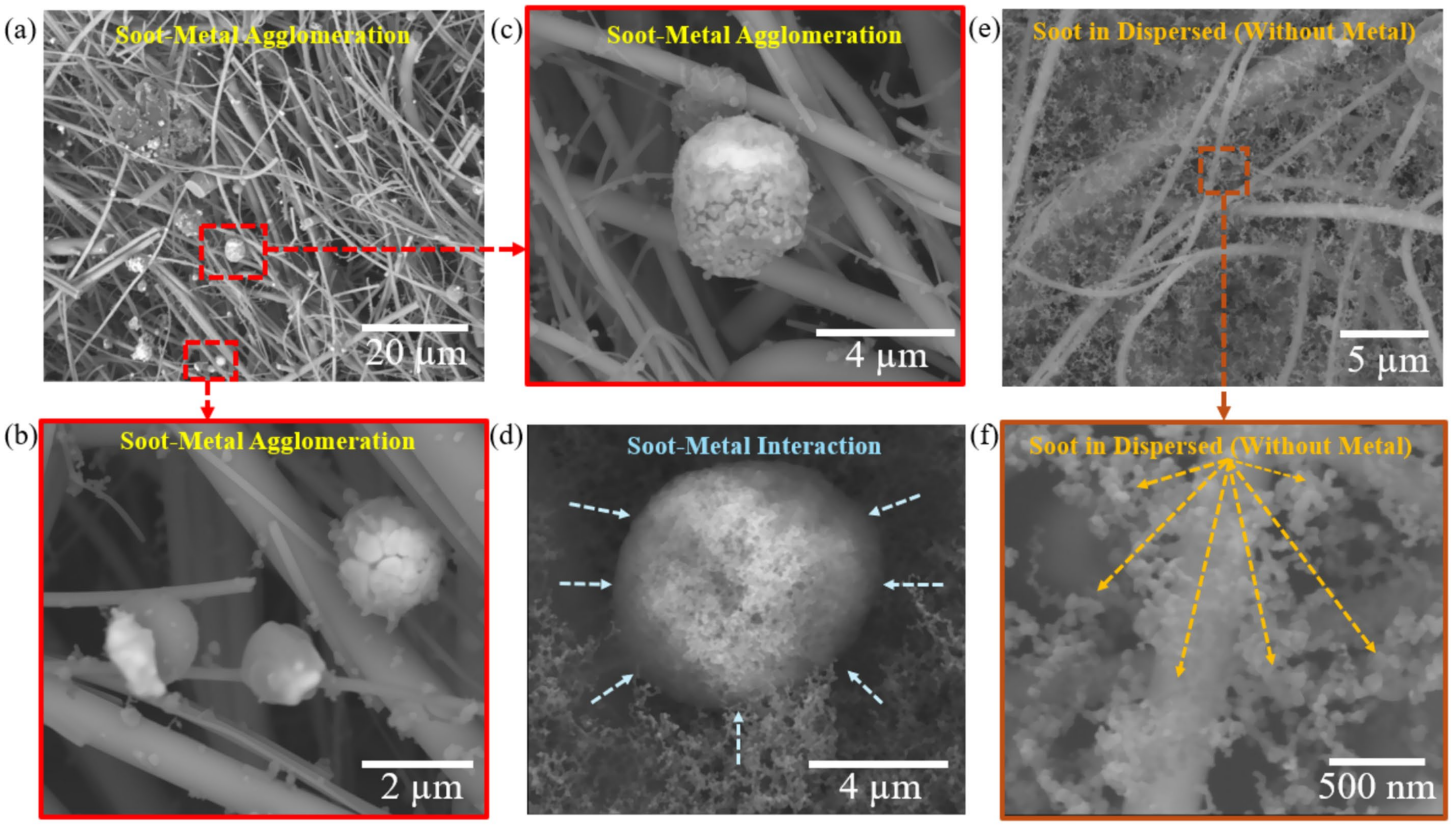
Soot and metal morphologies, interaction, and agglomeration at magnification of **(a)** Metals with soot at 1500×; **(b)** Soot-metal agglomeration at 15000×; **(c)** Soot-metal agglomeration at 10000×; **(d)** Soot and metal interaction at 10000×; **(e)** Soot in dispersed without metal interaction at 5000×; **(f)** Soot in dispersed without metal interaction at 50000×.

SEM and EDS mapping (Figure 8) provide direct mechanistic insight into how battery-derived metals restructure Pine soot, complementing the size-segregated chemical signatures described in Section 3.4. In the Pine + LIB scenario, low magnification (1,500×; Figure 8a) reveals densely packed filter surfaces containing bright, spherical-to-sub-spherical inclusions embedded within fibrous soot networks. At higher magnification (10,000–15,000×; Figures 8b–d), these inclusions appear as consolidated 0.2–0.8 μm agglomerates consisting of metal-rich cores enveloped by carbonaceous shells. EDS elemental mapping (see Supplementary Figures S3.2–S3.5) and ICP-MS analysis confirm substantial enrichment of Ni, Co, P, Al, Fe—precisely the dominant metals identified in Section 3.4 indicating that these species act as nucleation centers around which soot and semi-volatile organics condense and reorganize during plume cooling (29, 31, 82).

Without metal interaction, Pine soot (Figures 8e,f) appears as dispersed primary spherules, 0.02 to 0.04 μm in diameter, forming loose fractal aggregates with minimal compaction. These morphologies match classical descriptions of coniferous flaming soot, in which aggregation proceeds through weak van der Waals forces in the near absence of inorganic inclusions (34, 83–85). The lack of bright nucleation centers or welded clusters in Figures 8e,f underscores the structural transformations in Figures 8a–d is uniquely driven by the presence of battery metals during TR.

The transition from porous, fractal soot to persistent hybrid metal–soot agglomerates arises through three concurrent physicochemical pathways activated during NMC battery failure. First, hetero-coagulation is strongly enhanced by the surface charge characteristics of transition-metal oxides such as NiO and Co₃O₄, which carry zeta (*ζ*)-potentials following cathode degradation (29–31). Although Pine soot is also negatively charged, its surface exhibits patch-wise charge heterogeneity, allowing oppositely charged microdomains to facilitate attachment of metal nanoparticles via electrostatic bridging (34, 84, 85). This mechanism promotes rapid metal–soot association during the early cooling phase of the plume.

Second, the adsorption of metal nanoparticles substantially increases soot surface roughness and asperity density, amplifying van der Waals forces by raising the effective Hamaker constant (84). This enhanced short-range adhesion stabilizes multi-particle junctions, resulting in the compact, grape-like agglomerates observed in Figures 8b,c. These structures resist disintegration during sampling, consistent with other studies that show mixed metal–soot aggregates form within seconds in high-temperature combustion environments (86, 87).

Third, catalytic graphitization and partial sintering occur at metal–carbon interfaces. Transition metals such as Ni and Co are well-established catalysts for dehydrogenation, carbonization, and graphitization at temperatures ranging from 400 to 600 °C (36, 37, 88). These reactions lower activation barriers for C–C bond formation, generating thickened carbon shells and more ordered microstructures (36, 86, 88). The consolidated, layered morphologies observed in Figure 8d are consistent with catalytic restructuring—features absent in soot without metal interaction (Figures 8e,f).

These morphological pathways provide a coherent mechanistic link to the size-segregated metal and PAH results from earlier sections. The dominance of Ni, Co, Al, and Li in the fine mode (Section 3.4) reflects their role as metallic scaffolds that accrete soot and organics into submicron particles rather than remaining as isolated ultrafine nuclei (29, 30). Conversely, the high abundance of P in the ultrafine fraction matches the numerous <0.05 μm embedded nanoparticles visible in Figures 8b–d, consistent with nucleation of LiPF₆-derived phosphate/fluoride clusters described in lithium-ion fire literature (29, 31, 82). The pronounced enrichment of HMW PAHs in the ultrafine and fine modes (Section 3.5) further corresponds with the expanded surface area and strong adsorption affinity of these metal-decorated soot surfaces (86, 106).

Overall, these observations demonstrate that battery involvement does not simply increase metal emissions; it fundamentally reorganizes soot into metal-welded hybrid particles with greatly enhanced capacity to concentrate redox-active metals and carcinogenic PAHs in the respirable size range. These newly formed hybrids underpin the atmospheric persistence (Section 3.7) and the toxicological implications for inhalation exposure (Section 3.8), marking a profound shift from traditional Pine smoke toward a more hazardous emission class unique to battery-involved scenarios.

To concisely illustrate this emergent class distinction, Table 2 summarizes key particle modes, dominant metals, and HMW PAH enrichments across scenarios, highlighting the systematic progression toward metal–PAH hybrids in battery- and polymer-involved fires.

**Table 2:** Summary of particle characteristics defining a new class of Soot–Metal–PAH hybrid particles.

Fuel package representation with weight percentile (%)	Total PNC (#/cm^3^ ± SD)/Ultrafine %	Total PM concentration (μg/m^3^ ± SD)/ Ultrafine%	Total trace element concentration (μg/m^3^ ± SD) / (Total Ultrafine% /Fine%; Dominant elements Ultrafine%/Fine%)	Total PAH concentration (μg/m^3^ ± SD)/(HMW % in Ultrafine/ Fine; Dominant PAH% in Ultrafine)
Pure biomass (Pine 100 wt.%)	1.71 × 10^8^ ± 4.51 × 10^7^/81%	16 ± 0.89/50%	0.41 ± 0.05 μg/m^3^/ (Total Ultrafine 31%/Fine 40%; individual minimal)	ΣPAH 12.69 ng/m^3^, HMW% in Ultrafine/Fine: 35%/28% (None detected in 5–6 ring carcinogens)
LIB involved biomass (Pine 66 wt.% + NMC811 34 wt.%)	1.34 × 10^8^ ± 7.12 × 10^7^/86%	31.39 ± 3.65/19%	8.05 ± 0.39 μg/m^3^/ (Total Ultrafine 4%/Fine 73%; Ni 5.13%/ 50.5%, P 57.14%/ 10.45%, Li 12.31%/ 16.84%)	ΣPAH 24.23 ng/m^3^, HMW % in Ultrafine/Fine: 75%/77% (Chr 1.55 ng/m^3^, 15.7%; BbFL 1.46 ng/m^3^;14.8%; BaP 0.25 ng/m^3^; 2.6% in ultrafine)
Biomass with synthetic material (Pine 95 wt.% + PS 5 wt.%.%)	1.32 × 10^8^ ± 6.71 × 10^6^/52%	49.80 ± 3.09/23%	0.48 ± 0.05 μg/m^3^ (Total Ultrafine 32%/Fine 35%; individual minimal)	ΣPAH 44.12 ng/m^3^, HMW% in Ultrafine/Fine: 55% / 61% (FL 1.35 ng/m^3^, 14.67%; Pyr 1.08 ng/m^3^, 11.92%; BaP 0.35 ng/m^3^, 3.84% in Ultrafine)
LIB involved biomass and synthetic material (Pine 61 wt.% + PS 5 wt.% + NMC811 34 wt.%)	1.20 × 10^8^ ± 3.66 × 10^7^/55%	52.71 ± 0.09/20%	6.54 ± 0.39 μg/m^3^ (Total Ultrafine 3%/Fine 78%; Ni 3.58%/ 50.9%, P 50%/12.62%, Li 15.88/16%)	ΣPAH 29.51 ng/m^3^, HMW% in Ultrafine/Fine: 78% / 74% (FL 3.50 ng/m^3^, 37.39%; Pyr 1.87 ng/m^3^, 19.95%; BaP 0.08 ng/m^3^, 0.81% in Ultrafine)

### 3.7 Environmental hazard perspective of battery- and polymer-involved fire emissions

This study employed a controlled combustion chamber to quantify particulate emissions under reproducible, near-source conditions representative of short-term, high-intensity exposures. Each experimental fire was conducted for a fixed duration of 30 min, allowing for the time-integrated characterization of particle mass, number, size distribution, and chemical composition during active combustion. Real-time particle number and size distributions spanning 0.011–10 μm were measured using a SMPS (0.011–0.3 μm) and an OPS (0.3–10 μm), both operated with a constant 1:100 precision dilution to maintain concentrations within the instrument’s operating limits. PNC reported here were normalized to account for this dilution, whereas gravimetric and chemical analyses were performed on PM collected directly from the raw exhaust stream without dilution. As such, all reported concentrations represent raw duct values under controlled laboratory conditions and should be interpreted as hazard indicators for elevated short-term particulate burdens in near-source plumes, rather than as direct surrogates for ambient air quality metrics.

Although WUI fires typically occur in open environments, this chamber-based configuration is particularly relevant for understanding acute exposure scenarios experienced by firefighters and first responders. These populations frequently operate in proximity to flame fronts, within structural interiors, vehicle compartments, or other partially enclosed microenvironments where smoke concentrations can substantially exceed far-field ambient levels, affecting the general population (2, 8). Accordingly, direct comparisons with regulatory ambient guidelines are not appropriate due to differences in averaging time, dilution, and exposure duration; instead, the data provide insight into the relative hazard potential of different fuel combinations under identical combustion conditions.

Across the four fuel configurations, total PM increased systematically with increasing material complexity, from 16 ± 0.89 μg/m^3^ for pure Pine combustion to 31.39 ± 3.65 μg/m^3^ for Pine + LIB, 49.80 ± 3.08 μg/m^3^ for Pine + PS, and 52.71 ± 0.09 μg/m^3^ for Pine + PS + LIB. These values represent chamber-averaged concentrations integrated over the active combustion and sampling period and demonstrate the strong capacity of battery- and polymer-containing fuels to amplify particulate loading under short-duration, near-source conditions.

Battery involvement produced a particularly pronounced enhancement in particulate trace-element burdens. Total trace-element concentrations increased from 0.41 ± 0.05 μg/m^3^ in Pine to 8.05 ± 0.39 μg/m^3^ in Pine + LIB and 6.54 ± 0.39 μg/m^3^ in Pine + PS + LIB, while remaining comparatively low in Pine + PS (0.48 ± 0.05 μg/m^3^). Emissions were dominated by Ni, Li, P, Co, and Al, with more than 70% of the elemental mass consistently partitioning into the fine (0.1–2.5 μm) fraction. While not intended for regulatory comparison, qualitative benchmarking highlights the potential hazard posed by these emissions, underscoring the severity of short-term, near-field exposure scenarios for firefighters and first responders (2, 8, 89).

Polycyclic aromatic hydrocarbons burdens exhibited a similar progression, with Σ16 EPA PAHs increasing from 0.01 μg/m^3^ in Pine to 0.02 μg/m^3^ in Pine + LIB, 0.04 μg/m^3^ in Pine + PS, and 0.03 μg/m^3^ in Pine + PS + LIB. Although these concentrations are lower than those reported for some large-scale wildfire plumes, battery- and polymer-containing scenarios showed clear enrichment of HMW PAHs within ultrafine and fine particles. BaP, undetectable in pure Pine emissions, was consistently present in LIB- and PS-containing cases at sub- to low-ng/m^3^ levels, approaching or exceeding values commonly used as long-term ambient indicators when considered on a short-term exposure basis **(**89**)**.

Across all scenarios, ultrafine particles accounted for 52–86% of the total PNC, while contributing only 19–23% of the PM mass in battery- and polymer-involved cases. This pronounced number–mass decoupling indicates that chemically enriched, toxicologically relevant constituents are disproportionately associated with the smallest particles. Collectively, these findings suggest that emissions from pure biomass, such as Pine-only combustion, may substantially underestimate the hazard potential of modern WUI and battery-involved fires, particularly with respect to metal-rich and PAH-laden submicron aerosols (National **(**1, 2**)**).

### 3.8 Health hazard perspective: ultrafine carriers, carcinogenicity, and acute toxicological implications

From a public health standpoint, the dominant concern associated with battery- and polymer-involved fire emissions lies not solely in increased PM mass, but in the co-localization of ultrafine particles with carcinogenic metals and PAHs. Ultrafine particles (≤0.1 μm) possess high surface area–to–mass ratios, enabling deep penetration into the alveolar region and, in some cases, translocation beyond the pulmonary system (8, 17, 18, 90). Their toxicity is therefore driven more strongly by surface chemistry and associated constituents than by mass concentration alone.

In this study, ultrafine and fine particles generated during Pine + LIB and Pine + PS + LIB combustion carried concentrated loads of Ni, Co, P-rich species alongside HMW PAHs such as chr and BaP. These constituents are well-established drivers of oxidative stress, inflammatory signaling, mitochondrial dysfunction, and genotoxicity in pulmonary and cardiovascular tissues (8, 91–93). Although the chamber-derived concentrations cannot be directly extrapolated to ambient exposure metrics, their magnitude and chemical complexity are consistent with short-term exposure conditions encountered by firefighters and emergency responders operating near active fire zones.

Evidence from battery-specific toxicological studies further reinforces these concerns (16, 24). Aerosols emitted during LIB failure and fire events—particularly those involving Ni- and Co-rich cathode chemistries—have been shown to elicit stronger oxidative and cytotoxic responses than carbonaceous particles alone, with ultrafine fractions exhibiting disproportionately high toxicity per unit mass (24, 76).

Epidemiological evidence consistently links wildfire smoke exposure to increased respiratory and cardiovascular morbidity, particularly during short-term PM₂.₅ spikes that may exceed 100 μg/m^3^ during severe events (94–96). While most population-level studies do not resolve particle composition at the level reported here, the present results suggest that a mixed fuel package (pure biomass species like Pine, PS as representation of synthetic materials in urban interface and involving commonly used NMC811 batteries in consumer electronics and EVs), may generate aerosols with toxicological characteristics more closely resembling complex industrial combustion mixtures than traditional biomass smoke. This distinction is especially relevant for occupational exposure scenarios involving firefighters, who may experience repeated acute exposures across a fire season.

Current air quality standards emphasize PM₂.₅ and PM_10_ mass and long-term averages and do not explicitly address ultrafine particle number, metal–PAH co-association, or short-duration exposure peaks characteristic of fireground environments ((8, 11, 107). Recent assessments by the National Academies of Sciences, Engineering, and Medicine (2) and 108 have also highlighted these gaps. The findings presented here support the need for composition-aware hazard frameworks that explicitly consider ultrafine particle chemistry and battery-derived constituents when evaluating health risks associated with contemporary fire events.

## 4 Conclusion

This study presents the first size-resolved, chemically integrated characterization of particulate emissions from the co-combustion of biomass, a synthetic polymer, and NMC811 LIB under controlled, WUI-relevant flaming conditions. The results reveal that incorporating modern engineered materials, such as polystyrene and NMC811 LIB, fundamentally transforms wildfire smoke into a chemically and structurally distinct aerosol class that cannot be inferred solely from biomass combustion.

Pure pine combustion generated predominantly organic ultrafine particles accounting for ~81% of particle number, with relatively low particulate mass (16 ± 0.89 μg/m^3^), trace-element concentrations (0.41 ± 0.05 μg/m^3^), and PAHs (13 ng/m^3^, primarily low-molecular-weight species). In contrast, the addition of polystyrene and especially a single NMC811 battery induced a nonlinear restructuring of particle size distributions, shifting particulate mass, metals, and carcinogenic organics into the respirable fine mode (0.1–2.5 μm) and increasing total PM by up to ~3.3-fold (52.7 ± 0.09 μg/m^3^). Battery involvement increased total trace-element concentrations by more than 19-fold (8.05 ± 0.39 μg/m^3^), with Ni, Co, Li, Al, and P dominating.

Microscopic and chemical evidence demonstrates that battery thermal runaway produces phosphorus-rich ultrafine nuclei and Ni- and Co-bearing vapors that act as condensation scaffolds for soot and semi-volatile organics, forming compact metal–soot hybrid particles (0.2–0.8 μm) concurrently enriched in high-molecular-weight PAHs such as benzo[a]pyrene, chrysene, and benzo[b]fluoranthene. PAH concentrations reached 44.1 ng/m^3^ in Pine+PS combustion and 29.5 ng/m^3^ in the combined Pine + PS + LIB scenario, indicating substantial carcinogenic loading.

These findings identify an emergent class of soot–metal–PAH hybrid particles unique to mixed biomass–synthetic–battery combustion. From a scientific perspective, they show that modern wildfire aerosols are governed not only by vegetation chemistry but also by engineered materials that drive metal-catalyzed growth and toxicant enrichment. From an environmental and public-health perspective, they indicate that current air-quality frameworks, which emphasize particle mass alone, fail to capture acute toxicity associated with ultrafine, metal- and PAH-laden particles. As lithium-ion batteries and synthetic polymers increasingly intersect with wildfire regimes, composition-aware emission inventories, exposure models, and monitoring strategies will be required to assess risk and protect exposed populations accurately.

### 4.1 Limitations and future directions

This study employed a controlled-chamber combustion framework with a fixed radiant heat flux (50 kW.m^2^) and normoxic conditions (20.95% O₂) to enable mechanistic, size-resolved comparisons across fuel scenarios. However, real WUI fires involve greater variability in heat flux, oxygen availability, combustion phase, and fuel package composition. Future work will incorporate variable heat flux and ventilation to resolve flaming, mixed, and smoldering regimes. Only one LIB chemistry (NMC811) at 50% SOC was examined; future studies will assess higher SOCs and additional chemistry (e.g., LFP). While PS was used as a representative near-WUI synthetic material, future experiments will expand to PVC and other structural polymers commonly used in residential fuels. PAH analyses were conducted under single test conditions due to resource constraints; replicated testing is planned to quantify variability. Finally, field-scale validation during prescribed burns or real incidents is needed to translate these findings to occupational and public health exposure scenarios.

## Data Availability

The original contributions presented in the study are included in the article/Supplementary material, further inquiries can be directed to the corresponding author/s.

## Glossary

AcPy: Acenaphthylene
AcP: Acenaphthene
Al: Aluminium
Ant: Anthracene
BaA: Benzo[a]anthracene
BaP: Benzo[a]pyrene
BbFL: Benzo[b]fluoranthene
BghiP: Benzo[ghi]perylene
Bi: Bismuth
BkFL: Benzo[k]fluoranthene
Cd: Cadmium
Chr: Chrysene
Co: Cobalt
Cr: Chromium
Cu: Copper
DBA: Dibenz[a,h]anthracene
DLPI+: Dekati Low Pressure Impactor Plus
EC: Ethylene Carbonate
EDS: Energy-Dispersive X-ray Spectroscopy
EPA: U.S. Environmental Protection Agency
Fe: Iron
FE-SEM: Field-Emission Scanning Electron Microscopy
FL: Fluoranthene
Flu: Fluorene
GC–MS/MS: Gas Chromatography–Tandem Mass Spectrometry
HCL: Hydrochloric Acid (HCl)
HMW: High-Molecular-Weight
HNO_3_: Nitric Acid
H_2_O_2_: Hydrogen Peroxide
IARC: International Agency for Research on Cancer
ICP–MS: Inductively Coupled Plasma–Mass Spectrometry
InP: Indeno[1,2,3-cd]pyrene
K: Potassium
Li: Lithium
LIB(s): Lithium-Ion Battery/Batteries
LMW: Low-Molecular-Weight
MCE: Modified Combustion Efficiency
Mn: Manganese
NaP: Naphthalene
Ni: Nickel
NMC: Nickel–Manganese–Cobalt
OPS: Optical Particle Sizer
P: Phosphorus
PAH: Polycyclic Aromatic Hydrocarbon
PCTE: Polycarbonate Track-Etched
Ph: Phenanthrene
PM: Particulate Matter
PM₂.₅: Fine Particulate Matter (≤ 2.5 μm)
PM₁₀: Coarse Particulate Matter (≤ 10 μm)
PNC: Particle Number Concentration
PS: Polystyrene
PSD: Particle (Number) Size Distribution
Pyr: Pyrene
SMPS: Scanning Mobility Particle Sizer
SOC: State of Charge
TR: Thermal Runaway
UFP: Ultrafine Particles (≤ 0.1 μm)
VOC: Volatile Organic Compounds
WHO: World Health Organization
WUI: Wildland–Urban Interface
