# Supplementary material for: Wildland–urban interface co-combustion of biomass, synthetic polymeric materials, and lithium-ion batteries generates a new class of ultrafine soot–metal–PAH hybrid particles
Source: Front Public Health. 2026 Mar 3;14:1768652. doi: 10.3389/fpubh.2026.1768652 (PMC12992334; doi:10.3389/fpubh.2026.1768652)
Supplement: Supplementary file 1 [file Data_Sheet_1.docx]

****Wildland–Urban Interface Co-Combustion of Biomass, Synthetic Polymeric Materials, and Lithium-Ion Batteries Generates a New Class of Ultrafine Soot–Metal–PAH Hybrid Particles****

Md Jalal Uddin Rumi¹, Yulin Wu², Md. Jakir Hossain¹, Mazyar Etemadzadeh², Mengying Zhang², Todd A. Kingston¹, Rui Li², and Guowen Song²*

¹Department of Mechanical Engineering, Iowa State University, Ames, IA, United States
²Department of Apparel, Events, and Hospitality Management, Iowa State University, Ames, IA, United States

* Correspondence: Guowen Song

gwsong@iastate.edu

# Graphical Abstract


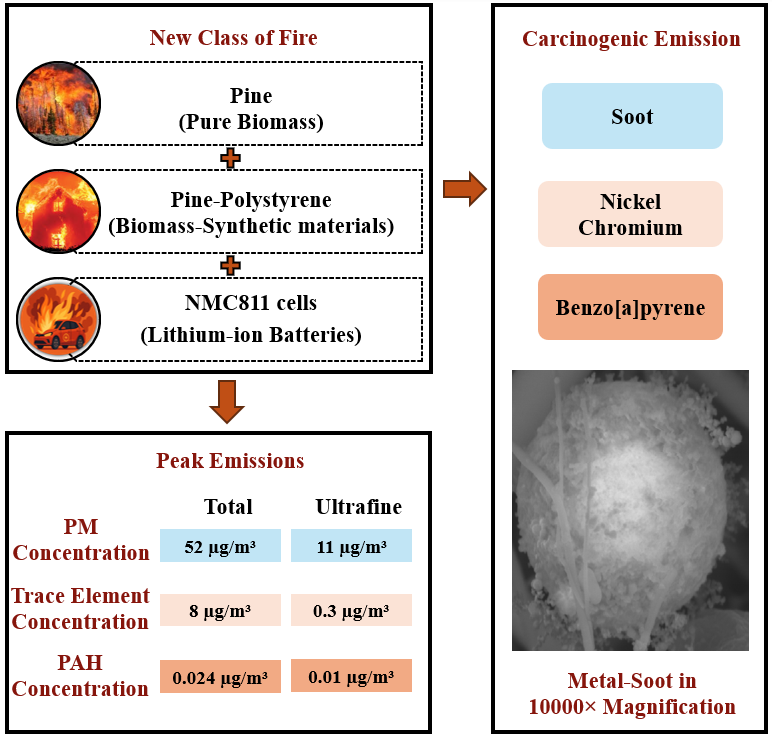


# Abstract

# Wildland–urban interface (WUI) fires increasingly involve the co-combustion of biomass with synthetic polymer materials and lithium-ion batteries (LIBs); yet the resulting particulate emissions and their ultrafine-particles (UFP, <0.1 µm) contribution remain poorly characterized. This study presents a size- and composition-resolved analysis of particulate matter (PM), trace elements, and polycyclic aromatic hydrocarbons (PAHs) generated under controlled, near-source flaming conditions (50 kW/m² radiant heat flux; 20.95% O₂). Four fuel configurations representing contemporary WUI scenarios were examined: pure biomass (Pine), biomass with an NMC811 LIB (Pine + LIB), biomass with a synthetic material (Pine + PS), and biomass with both a synthetic material and an NMC811 LIB (Pine + PS + LIB).

# Pure pine combustion produced UFP-dominated emissions (~81% of particle number concentration, PNC) with relatively low concentration of PM (15.9 ± 0.89 µg/m³), trace elements (0.41 ± 0.05 µg/m³), and Σ16 EPA PAHs (12.7 ng/m³), primarily low-molecular-weight species. In contrast, NMC811 LIB and/or PS involvement induced pronounced number–mass decoupling: UFPs remained dominant by number (52–86%), while PM mass shifted toward the fine fraction (0.1–2.5 µm), increasing total PM by up to 3.3-fold (52.7 ± 0.09 µg/m³). Battery involvement led to a more than 19-fold increase (8.05 ± 0.39 µg/m³) in particulate trace elements, dominated by nickel, lithium, phosphorus, cobalt, and aluminum. Microscopic analysis revealed metal–soot hybrid particles indicative of NMC811 battery thermal runaway at a 50% state of charge condition. PAH concentrations increased concurrently, reaching 44.1 ng/m³ in Pine+PS combustion and 29.5 ng/m³ in the combined Pine+PS+LIB scenario, with preferential enrichment of carcinogenic high-molecular-weight PAHs in ultrafine and fine particles.

Although not intended for direct regulatory comparison, these near-source concentrations indicate elevated short-term hazard potential relevant to firefighters and first responders. Overall, the findings demonstrate that battery- and polymer-involved WUI fires generate compositionally distinct, respirable particles that challenge biomass-only emission frameworks and mass-based air quality metrics.

Keywords: Wildland–Urban Interface Fires; Lithium-Ion Battery; Thermal Runaway; Ultrafine Particles; Metal and PAH Emissions; Public Health Risk.

# 1. Introduction

Wildland and wildland–urban interface (WUI) fires are becoming increasingly frequent, larger, and longer-lasting due to the combined influences of climate change, fuel accumulation, and the continued expansion of housing into fire-prone regions (United Nations Environment Programme, 2022; National Academies of Sciences, Engineering, and Medicine, 2022). Global assessments project that extreme wildfire events will rise by up to 14% by 2030, 30% by 2050, and 50% by the end of the century, making WUI communities and ecosystems particularly vulnerable (United Nations Environment Programme, 2022). Recent events illustrate the scale and rapid escalation of these hazards: the 2023 Lahaina fire in Maui destroyed over 2,200 structures and caused 102 fatalities (US Fire Administration, 2024); the 2023 Canadian fires burned approximately 15 million hectares, the largest area in recorded history (Natural Resources Canada, 2024); and the January 2025 Eaton and Palisades fires near Los Angeles resulted in extensive structural loss, prolonged smoke exposure, and major economic disruption (Kimelman, 2025). These cases underscore the urgent need to understand the evolving chemical nature and toxicity of WUI smoke.

From a public health standpoint, wildfire smoke is primarily evaluated through fine particulate matter (PM₂.₅, 0.1-2.5 µm) and coarse particulate matter (PM_10_, 2.5-10 µm) mass, which forms the basis of air quality advisories and acute exposure guidelines (World Health Organization, 2021; U.S. Environmental Protection Agency, 2024). A large epidemiological literature associates wildfire-derived PM₂.₅ with increased respiratory and cardiovascular morbidity, including asthma exacerbations, chronic obstructive pulmonary disease (COPD) flare-ups, pneumonia, ischemic heart disease, and cardiac arrhythmias, and with disproportionate impacts on children, older adults, and underserved populations (Riss et al., 2025; Zhang et al., 2025; Wei et al., 2025). Meta-analyses indicate that a 1 µg/m³ increase in wildfire PM₂.₅ is associated with measurable rises in respiratory hospital admissions, and health effects can persist for weeks to months following major fire events (Zhang et al., 2025; Wei et al., 2025). However, this regulatory and scientific focus on mass-based PM₂.₅ and PM_10_ metrics inherently obscures the role of ultrafine particles (UFPs, <0.1 µm) a fraction capable of deep lung penetration, translocation into the bloodstream, and efficient transport of redox-active metals and organic toxicants, yet largely absent from wildfire monitoring and risk frameworks (Health Effects Institute, 2013; Kwon et al., 2020; Rumi et al., 2025). As WUI fires intensify and the composition of fuels shifts, the PM₂.₅ and PM_10_ paradigm increasingly fails to capture the true toxicological profile of emerging smoke sources.

The chemical composition of smoke in WUI scenarios differs fundamentally from that of traditional biomass-only wildfires. The burning of vegetation now frequently co-occurs with synthetic polymer materials from the built environment (e.g., polystyrene insulation, plastics, foams) and with lithium-ion batteries (LIBs) embedded in electric vehicles (EVs) and consumer electronics (Castaño-Ortiz et al., 2024; Holder and Sullivan, 2024; National Academies of Sciences, Engineering, and Medicine, 2022; Rumi et al., 2025). Real-world events, such as the Lahaina and Los Angeles fires, demonstrate that mixed-fuel beds comprising wood, plastics, textiles, metals, and electronic components produce highly heterogeneous smoke plumes with complex physicochemical interactions. Despite this rapid shift in fire combustion, little is known about how mixed-fuel combustion alters particle formation pathways, toxic metal emissions, or carcinogenic organic compounds relative to biomass-alone combustion.

This knowledge gap is especially critical, given the accelerating adoption of LIBs in EVs and consumer electronics within WUI zones (Liu et al., 2020; Rumi et al., 2025). Among different LIB cathode chemistries—including lithium cobalt oxide (LCO), lithium manganese oxide (LMO), lithium nickel manganese cobalt oxide (NMC), lithium nickel cobalt aluminum oxide (NCA), lithium iron phosphate (LFP), and lithium titanate (LTO)—Ni-rich NMC variants, particularly NMC811, dominate high-performance applications due to their superior energy density and cost-effectiveness (Liu et al., 2022; Wang et al., 2023). Nonetheless, elevated nickel (Ni) content compromises thermal and structural stability, heightening vulnerability to thermal runaway (TR) under different abusive conditions (Chen et al., 2020; Meister et al., 2025).

TR in LIBs initiates a cascade of exothermic reactions, leading to uncontrolled temperature spikes and potential cell rupture (Feng et al., 2014; Feng et al., 2018; Jiaqiang et al., 2024; Wang et al., 2019; Zhou et al., 2022). TR can be initiated by mechanical (e.g., crushing), electrical (e.g., overcharging, external short circuit), or thermal (e.g., overheating) abuse. Under these different abuse conditions, TR typically commences with the decomposition of the solid electrolyte interphase (SEI), progressing to electrolyte solvent vaporization, cathode oxygen release, and inter-electrode reactions (Feng et al., 2018; Meister et al., 2025). **Importantly, TR is not an inherent outcome for all LIB fires but rather depends on multiple interacting factors, including cathode chemistry, state of charge (SOC), abuse mode, environmental conditions, LIB design, and capacity, among others (Wang et al., 2025).** In general, **higher SOC (e.g., 50–100%) increases the likelihood and severity of TR by elevating stored chemical energy and accelerating exothermic decomposition reactions (Han et. al., 2025; Wang et al., 2025).** During these events, LIBs release metal-rich particulates (Premnath et al., 2022; Claassen et al., 2024a; Claassen et al., 2024b).

Simultaneously, polystyrene (PS) thermally degrades into styrene monomers and aromatic oligomers that polymerize and aromatize into soot, enhancing yields of high-molecular-weight (HMW) polycyclic aromatic hydrocarbons (PAHs) relative to biomass combustion, which primarily generates lower-molecular-weight (LMW) PAHs (Goldsmith et al., 2025; Younis et al., 2025; Vicente & Alves, 2018; Shrivastava et al., 2024; Zhang et al., 2024).

**Under WUI-relevant conditions, the simultaneous release of metal-rich particles from LIB TR and PAH-rich soot from biomass–polymer combustion provides a mechanistically plausible pathway for enhanced particle mass loading and chemical complexity, as observed in the present study.** **Although prior studies have independently characterized LIB-derived metal emissions and PAH formation from biomass–plastic combustion, their combined effects on particle composition and size-resolved toxicant enrichment have not been systematically quantified.** Transition metals and other trace elements associated with LIB cathode materials may further influence soot structure and surface chemistry by catalyzing carbon reorganization or stabilizing PAHs on particle surfaces, processes previously demonstrated in controlled metal–carbon systems (Pudikov et al., 2018; Kim et al., 2020). **These interactions suggest, but do not yet establish, that battery-involved WUI smoke may exhibit greater toxicological potency than biomass-only smoke.**

Despite this mechanistic understanding, substantial research gaps persist. Most LIB TR studies focus on bulk PM₂.₅ and gaseous toxicants such as Hydrogen Fluoride (HF), Carbon Monoxide (CO), and volatile organic compounds (VOCs) while providing limited resolution of the specific hazards of UFPs (Bugryniec et al., 2024; Feng et al., 2018; Larsson et al., 2017; Rumi et al., 2025; Sahil et al., 2023). At the same time, WUI combustion studies rarely investigate realistic mixed-fuel packages that burn biomass, synthetic materials, and LIBs concurrently, despite such conditions being prevalent in real-world fire scenarios (Castaño-Ortiz et al., 2024; Holder and Sullivan, 2024; National Academies of Sciences, Engineering, and Medicine, 2022). Very few experiments offer size-resolved characterization of metals and PAHs under harmonized conditions, leaving major uncertainties regarding how these toxicants partition across ultrafine, fine, and coarse modes or how hybrid metal–soot–PAH particles form and evolve in battery-involved WUI fires (National Academies of Sciences, Engineering, and Medicine, 2022; Rumi et al., 2025). These gaps limit the accuracy of emission inventories, constrain exposure and dispersion modeling, and hinder the development of evidence-based protection strategies for firefighters and nearby communities.

Air-quality regulations compound this challenge. Existing standards emphasize PM₂.₅ and PM₁₀ mass while lacking metrics for UFP number concentrations, compositionally enriched PM, or metal-bearing UFPs despite longstanding evidence of their heightened biological reactivity (World Health Organization, 2021; U.S. Environmental Protection Agency, 2024; Health Effects Institute, 2013). This mismatch between emerging fire chemistry and regulatory indicators underscores the need for controlled scenario-specific studies that integrate physical and chemical particle characterization across size modes.

To address these gaps, the present study systematically investigates emissions from four fuel configurations representing an escalation from biomass alone to starting proximity of WUI conditions: pure biomass (Pine), biomass with NMC811 LIB (Pine + LIB), biomass with a representation of synthetic material (Pine + PS), and biomass with both synthetic material and NMC811 LIB (Pine + PS + LIB). Under a uniform 50 kW/m² radiant heat flux and 20.95% oxygen (O₂), we measured real-time particle number concentration (PNC) across size distributions (0.011–10 µm) using a scanning mobility particle sizer (SMPS, NanoScan 3910, TSI Inc.) and an optical particle sizer (OPS, 3330, TSI Inc.). Also, size-segregated particulate matter was collected (0.016–10 µm) via Dekati Low Pressure Impactor (DLPI+) cascade impactor for multi-element metal quantification via Inductively Coupled Plasma–Mass Spectrometry (ICP–MS), U.S. Environmental Protection Agency (EPA)-priority 16 PAH speciation via Gas Chromatography–Tandem Mass Spectrometry (GC–MS/MS), and microstructural analysis via Field-Emission Scanning Electron Microscopy coupled with Energy-Dispersive X-ray Spectroscopy (FE-SEM/EDS). We hypothesize that mixed-fuel combustion involving LIBs and PS will substantially alter emission profiles relative to biomass alone, producing ultrafine and fine particles enriched in transition metals and carcinogenic PAHs, with potential exceedances of short-term exposure benchmarks.

By integrating particle dynamics, size-resolved chemistry, and microstructural insights, this study aims to elucidate the formation of ultrafine metal–soot–PAH hybrids in battery-involved WUI fires and to provide exposure-relevant metrics for emission inventories, firefighter protection, and public health risk assessment.

# 2. Materials and methods

## 2.1. Selection and preparation of pine and polystyrene materials

To reflect the starting proximity of WUI fires where biomass, synthetic materials, and embedded LIBs burn concurrently, this study selected pine as pure biomass (the representative wildland fuel) and polystyrene (PS) as a starting proxy for common synthetic materials and furnishings present in North American WUI communities (National Academies of Sciences, Engineering, and Medicine, 2022).

Both Pine and PS were processed into standardized geometries to ensure reproducible burning conditions. Bulk material was first cut into 100 × 100 mm pieces and then milled to a nominal particle size of 500 µm using a hammer mill (Schutte-Buffalo, USA). The resulting powders were compressed into cylindrical briquettes (100 mm diameter, 13 mm thickness, bulk density 1.0 g/cm³) using a hydraulic press (MTS, USA). This approach, adapted from prior WUI fuel investigations (Maranghides et al., 2021), minimized heterogeneity in packing, airflow, and heating rate compared with unprocessed boards.

All fuel discs were conditioned in an environmental chamber at 21 ± 2 °C and 65 ± 5% relative humidity for a minimum of 24 h prior to testing to reduce variability in moisture content, in line with established procedures for standardized combustion experiments (Davis et al., 2025; Jen et al., 2019). Elemental composition (C, H, N, S, O) was determined using a CHNS/O analyzer (FlashSmart™, Thermo Scientific, USA); full compositional data are provided in the supplemental section S.2.1 and Table S.2.1.

## 2.2. Lithium-ion battery characteristics

Cylindrical 18650-format LIBs (18 mm diameter × 65 mm length) with NMC cathode (LiNi₀.₈Mn₀.₁Co₀.₁) chemistry were selected due to their prevalence in EVs and consumer electronics (Liu et al., 2020; Wang et al., 2023). Each NMC811 LIB had a nominal capacity of 2500 mAh, a nominal voltage of 3.7 V, an upper cut-off voltage of 4.2 V, a lower cut-off of 2.5 V, a rated maximum continuous discharge current of 20 A, and a typical mass of ~43 g. NMC811 LIB was embedded along with pine and PS to prepare the fuel package (Section 2.4 Test matrices and experimental conditions), reflecting their widespread deployment in consumer electronics, EVs in residential and urban settings within WUI zones (National Academies of Sciences, Engineering, and Medicine, 2022; Rumi et al., 2025).

Proprietary information on the internal electrolyte composition and electrode formulations was not disclosed in the manufacturer's safety data sheet. Therefore, cathode chemistry was independently verified by EDS analysis of pristine electrode samples. The Ni:Mn:Co atomic ratio was approximately 7.8:1:1.4, consistent with an NMC811-type cathode (Yan and Ezekoye, 2023).

## 2.3. Galvanostatic cycling and state-of-charge conditioning

To establish baseline electrochemical performance for accurate SOC determination, pristine batteries were subjected to three galvanostatic cycles at C/10 (0.25 A) between 2.7 and 4.2 V using a Neware BTS-5V12A testing system at room temperature. Charging durations were determined by each LIB’s measured capacity and then charged to achieve 50% SOC. The selected SOC is consistent with prior work linking TR severity and emissions to partial charge levels (Claassen et al., 2024a; Claassen et al., 2024b; Chen et al., 2020). The 100% SOC condition was omitted due to the risk of explosion from excessive gas generation in NMC811 LIB (Feng et al., 2019).

2.4 Test matrices and experimental conditions

Four fuel configurations were evaluated, as listed in Table 1, to represent emissions from wildland, battery-involved wildland, WUI mixed-fuel, and battery-involved WUI. This structured matrix ensures comparability across scenarios and enables direct evaluation of how batteries and synthetic materials modify particle formation, chemical composition, and toxicant emissions. All experiments were conducted under identical conditions (50 kW/m²; 20.95% O₂) for a fixed duration of 30 minutes to isolate the influence of fuel composition, combustion conditions on emission profiles. Smoke emission characterization (including particle number, mass, and size distribution) and trace element analysis by ICP-MS were performed in triplicate (n = 3) per fuel package to ensure statistical robustness (Meister et al., 2025), while PAH analysis by GC-MS/MS was conducted once (n = 1) per fuel package due to resource constraints.

Table 1. Fuel packages and thermal exposure conditions used in combustion experiments.

| Sample ID | Fuel package representation | Fuel materials and weight percentile (%) | Weight (g) | Heat flux (kW/m²) | Oxygen (%) |
| --- | --- | --- | --- | --- | --- |
| Pine | Pure biomass  (Wildland) | Pine 100 wt.% | Pine: 105 ± 1.50 | 50 | 20.95 |
| Pine + LIB | LIB involved biomass  (Hybrid wildland) | Pine (66 wt.%) + NMC811 LIB (34 wt.%) | Pine: 82.32 ± 1.53  NMC811: 42.68 ± 0.08 | 50 | 20.95 |
| Pine + PS | Biomass with synthetic material  (WUI mixed fuel) | Pine (95 wt.%) + PS (5 wt.%) | Pine: 100 ± 1.25  PS: 5 ± 0.25 | 50 | 20.95 |
| Pine + PS + LIB | LIB involved biomass and synthetic material (Hybrid WUI) | Pine (61 wt.%) + PS (5 wt.%) + NMC811 LIB (34 wt.%) | Pine: 75.94 ± 1.52  PS: 6.28 ± 0.25  NMC811: 42.78 ± 0.03 | 50 | 20.95 |

## 2.5. Experimental fire simulation setup

The tests were carried out in a custom-built, atmosphere-controlled combustion chamber designed to meet ISO/TS 5660-5 specifications (International Organization for Standardization, 2020). The configuration was designed to simulate intense, localized heating conditions encountered during structural or compartment fires, while maintaining experimental control (Fig. 1).


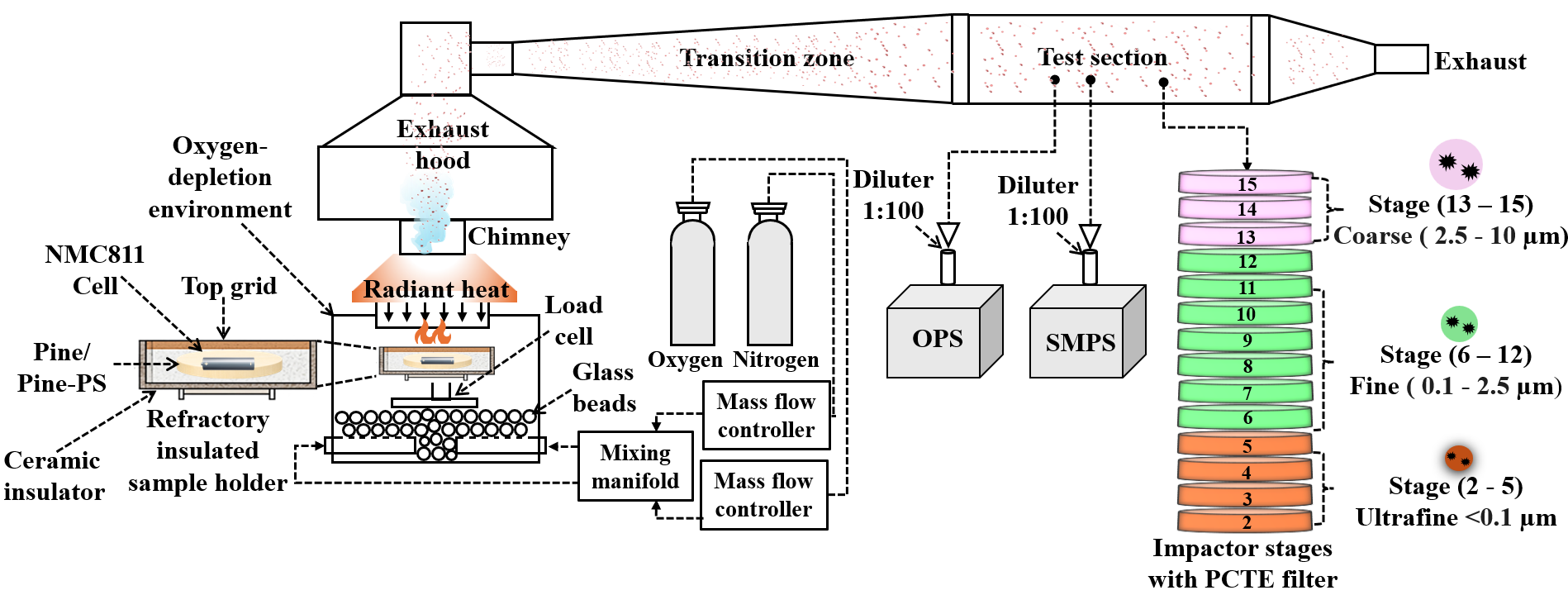


Fig.1. Schematic of the experimental test facility, including the combustion chamber, smoke tunnel, and instrumentation, replicating full-scale smoke exposure pathways during different fire scenarios.

The fuel package was mounted in an ASTM E1354-compliant holder equipped with a stainless-steel mesh restraint to prevent the ejection of fragments during thermal runaway. Heating was provided by a conical radiant heater delivering the prescribed 50 kW/m² heat flux (International Organization for Standardization, 2020).

Gas distribution within the chamber was homogenized using a glass-bead dispersion layer, which promoted uniform temperature and oxidant delivery across the sample region (Reid et al., 2016; Teixeira et al., 2024). Combustion effluents were conveyed through a 265 cm exhaust duct at a bulk flow rate of 42 L/s (test-section air velocity ~0.75 m/s), ensuring sufficient residence time and mixing before sampling (Fig. 2). This configuration enabled integrated collection of particulates representative of near-field exposure conditions in enclosed or semi-enclosed environments. Although WUI fires predominantly occur in open air, the controlled lab-based combustion setup mimics the high-concentration, short-term exposures encountered by firefighters in proximity to WUI fires, where smoke particle levels are significantly elevated compared to far-field exposures affecting the general population (National Academies of Sciences, Engineering, and Medicine, 2022; Rumi et al., 2025). This relevance stems from firefighters' duties in semi-enclosed microenvironments during WUI incidents, enabling insights into acute health risks for first responders.

## 2.6. Particulate sampling, size segregation, and collection for chemical and morphological analyses

A multi-instrument sampling train was installed downstream of the combustion chamber (see Fig. 1) to quantify particle number, mass, and their size distributions, as well as to perform morphological, trace elements, and chemical characterizations, as shown in Fig. 2.

Each experimental fire was conducted for a fixed duration of 30 minutes. Real-time particle number and size distributions (0.011 to 10 µm) were captured using a SMPS (0.011 to 0.3 µm) and an OPS (0.3 to 10 µm), each coupled to precision diluters (1:100) to ensure concentrations remained within instrument-specific limits (Moreno et al., 2020). This dilution for OPS and SMPS was applied throughout the full 30-minute experimental duration.


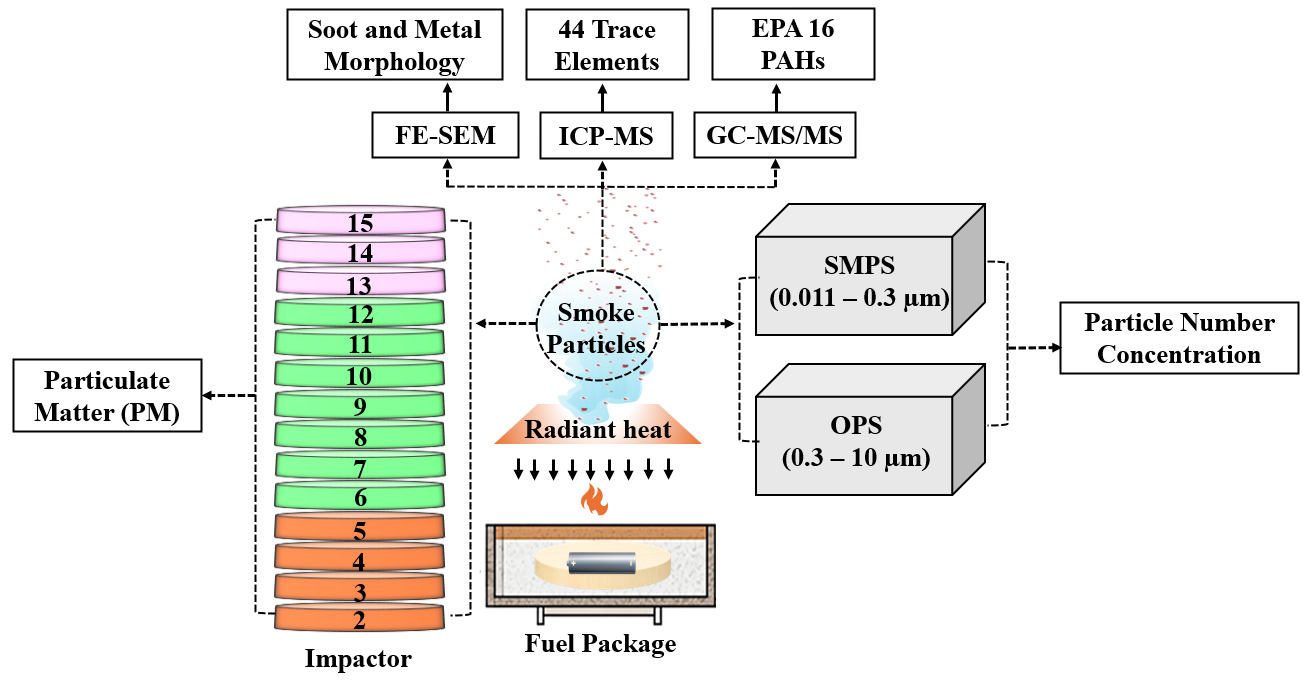


Fig. 2. Multi-instrument workflow illustrates particle collection, size fractionation, and chemical/morphological characterization of emissions from different fire scenarios.

For size-resolved mass, metal, and chemical characterization, particulates were collected using a DLPI+ cascade impactor directly from the raw exhaust stream without dilution at 10 L/min, covering 0.016–10 µm aerodynamic diameters and enabling size separation into ultrafine (stage 2 to 5), fine (stage 6 to 12), and coarse (stage 13 to 15) fractions consistent with previous lithium-ion and combustion and ambient particulate studies (Kontkanen et al., 2020; Claassen et al., 2024a; Claassen et al., 2024b; Fent et al., 2018). See supplemental sections S.2.2 and Table S.2.2 for the detailed methodology for particle collection using DLPI+. Particulates were deposited onto 0.1 µm polycarbonate track-etched (PCTE) membrane filters mounted on DLPI+ stages, which were subsequently used ICP–MS to quantify 44 trace elements (see supplemental section Table S.3.3 for the list of 44 elements), and GC–MS/MS to determine concentrations of the 16 EPA-priority PAHs (see section 3.5).

For SEM–EDS characterization, particulates were collected using a sampling tube equipped with XAD-2 resins connected to Buck Elite pumps (A.P. Buck Inc., FL, USA), operated at 2 L/min (Kesler et al., 2025). The sampler was fitted with 37 mm quartz fiber filters (AQFA03700, MilliporeSigma, USA) to collect particulates for SEM–EDS characterization (Kesler et al., 2025). Sample preparation workflows for each analytical technique are described in sections 2.7–2.9.

## 2.7. Polycyclic aromatic hydrocarbon analysis by Gas Chromatography–Tandem Mass Spectrometry

Size-resolved PAH emissions were quantified to characterize the organic toxicant profile of fuel packages replicating different fire scenarios (Claassen et al., 2024a; Fent et al., 2018; Meister et al., 2025; Teixeira et al., 2024).

Sampling followed U.S. EPA Method TO-13A (U.S. EPA, 1999). Gas- and particle-phase PAHs were collected using XAD-2 cartridges (PUF–XAD-2–PUF, Restek, USA), 0.1 µm PCTE filters, and the DLPI+ impactor for size fractionation. The EPA 16 priority PAHs were analyzed using a GC–MS/MS system (Agilent 7250 Q-TOF) equipped with a DB-5ms column (30 m × 0.25 mm i.d., 0.25 µm film). The oven program ramped from 50 °C (held 1 min) to 320 °C at 10 °C/min, with a final hold of 5 min. Helium was used as the carrier gas at a flow rate of 1 mL/min, and ionization was performed at 70 eV (Pandey et al., 2011).

Sample extraction was carried out using an automated Accelerated Solvent Extractor (ASE 350, Thermo Fisher, MA, USA), typically employing dichloromethane as the extraction solvent. Extracts were subsequently filtered and concentrated under a gentle nitrogen stream prior to injection (Teixeira et al., 2024). Method performance was verified through multi-point calibration (R² > 0.99), with method detection limits ranging from 0.01 to 0.1 ng/m³ and quantification limits from 0.03 to 0.3 ng/m³. Surrogate standard recoveries between 60% and 120% were accepted (Fent et al., 2018). Additional method parameters, recoveries, and Quality Assurance/Quality Control metrics are presented in supplemental section S.2.3 and Tables S.2.3–S.2.4.

## 2.8. Particle morphology and elemental mapping by Field-Emission Scanning Electron Microscopy coupled with Energy-Dispersive X-ray Spectroscopy

To elucidate the microstructural features and elemental composition of particulates, FE-SEM coupled with EDS was employed (Adanouj et al., 2023; Claassen et al., 2024). Particulates collected on a 37 mm quartz filter were examined using an FEI Quanta-FEG 250 microscope equipped with an Oxford AZtec EDS system.

Prior to imaging, samples were coated with a ~5 nm iridium layer using a Quorum Q150T ES sputter coater to improve electrical conductivity and reduce charging artifacts (Goldstein et al., 2018). Imaging was performed under low-vacuum conditions (0.1–0.3 Torr) at an accelerating voltage of 10 kV, a working distance of 10.8–11.3 mm, and a spot size of 3.0–4.0 µm (Adanouj et al., 2023). Both secondary and backscattered electron modes were used at magnifications ranging from 50× to 50,000× to resolve particle morphologies, aggregate structures, and surface features.

EDS mapping was applied to identify carbonaceous matrices and metal-bearing inclusions consistent with NMC LIB components and combustion residues. As is typical for SEM–EDS, detection of low-atomic-number elements (e.g., lithium) was limited by poor X-ray yield and matrix absorption, and quantification of trace elements in dense carbon matrices was constrained by background and overlapping peaks (Goldstein et al., 2018). These limitations motivated the complementary use of ICP–MS for high-sensitivity elemental analysis.

## 2.9. Trace element quantification by Inductively Coupled Plasma–Mass Spectrometry

To provide a comprehensive and sensitive assessment of metal, metalloids, and nonmetal emissions, ICP–MS was employed for light elements and trace elements that are not reliably detected by SEM–EDS (Goldstein et al., 2018; Claassen et al., 2024a). Analyses were conducted using a NexION 2000B ICP–MS (PerkinElmer, Waltham, MA, USA) targeting 44 elements, including key battery-relevant metals such as nickel (Ni), manganese (Mn), cobalt (Co), lithium (Li), aluminum (Al), phosphorus (P), and silicon (Si). See the supplemental section, Table S.3.3, for the full 44-trace element list.

Particles were collected on 0.1 µm PCTE filters, chosen for their high collection efficiency and compatibility with acid digestion. Each filter was placed in a digestion tube containing 7 mL of an extraction mixture comprising 2.1% hydrogen peroxide (H₂O₂), 11% nitric acid (HNO₃), and 28.5% hydrochloric acid (HCl). Samples were digested on a hot block at 80 °C for 2 h to dissolve particulate-bound metals. After cooling, digests were quantitatively transferred and diluted to 25 mL with deionized water, then allowed to equilibrate for 12 h to ensure solution stability.

Aliquots (~2 mL) of the digested samples were introduced into the ICP–MS via a nebulizer, where the particulates were ionized in an argon plasma. The resulting ions were separated by mass-to-charge ratio and quantified against multi-element calibration standards. This method enabled the detection of low limits for a broad suite of elements, including transition metals and rare earths relevant to NMC battery chemistries, providing a robust complement to SEM–EDS for characterizing the metallic component of combustion particulates.

# 3. Results and discussion

## 3.1 Transformation of particle dynamics across fire scenarios

Particle number concentration (PNC) and particulate mass (PM) across the four fuel configurations—Pine (100 wt.%), Pine+LIB (Pine 66 wt.% + LIB 34 wt.%), Pine+PS (Pine 95 wt.% + PS 5 wt.%), and Pine+PS+LIB (Pine 61 wt.% + PS 5 wt.% + LIB 34 wt.%)—reveal a systematic restructuring of the particulate from number-dominated UFPs to mass-dominated fine and coarse aggregates (Fig. 3a–c).

Pine combustion produced the highest total PNC 1.71 × 10⁸ ± 4.51 × 10⁷ #/cm³. The addition of an NMC811 as LIB and/or PS as synthetic material reduced total PNC to 1.34 × 10⁸ ± 7.12 × 10^7^ #/cm³ (−22%) for Pine+LIB, 1.32 × 10⁸ ± 6.71 × 10^6^ #/cm³ (−23%) for Pine+PS, and 1.20 × 10⁸ ± 3.66 × 10⁷ #/cm³ (−30%) for Pine+PS+LIB. In contrast, total collected PM increased from 1.20 ± 0.07 mg in Pine to 2.37 ± 0.28 mg (+98%) in Pine+LIB, 3.77 ± 0.23 mg (+214%) in Pine+PS, and 3.99 ± 0.01 mg (+233%) in Pine+PS+LIB. This inverse behavior of PNC and PM mass reflects accelerated coagulation and condensation driven by higher inorganic/organic loading from the synthetic material components, resulting in a marked shift from nucleation-dominated UFPs toward accumulation- and coarse-mode aggregates (Islam et al., 2022; Wu et al., 2025; Xiao et al., 2025).

Size-resolved contributions (Fig. 3b, c) reinforce this transition. In Pine, UFPs (<0.1 µm) dominated number emissions, contributing ~81% of total PNC (1.38 × 10⁸ ± 4.04 × 10⁷ #/cm³), while fine particles contributed ~19% (3.32 × 10⁷ ± 6.86 × 10⁶ #/cm³) and coarse particles were negligible (<0.01%). By mass, Pine emissions were more evenly distributed, with UFPs contributing ~50% (0.61 ± 0.16 mg), fine particles ~46% (0.55 ± 0.06 mg), and coarse particles ~4% (0.04 ± 0.04 mg). This is consistent with high-MCE flaming combustion of coniferous biomass, where abundant low-volatility VOC oxidation products drive rapid homogeneous nucleation and condensation, producing dominant ultrafine modes (number-weighted particle diameter, Dp ~0.06 –0.07 µm) with limited inorganic seeding or coagulation sinks in fresh plumes (Chen et al., 2023; McClure et al., 2020; Shrivastava et al., 2024).


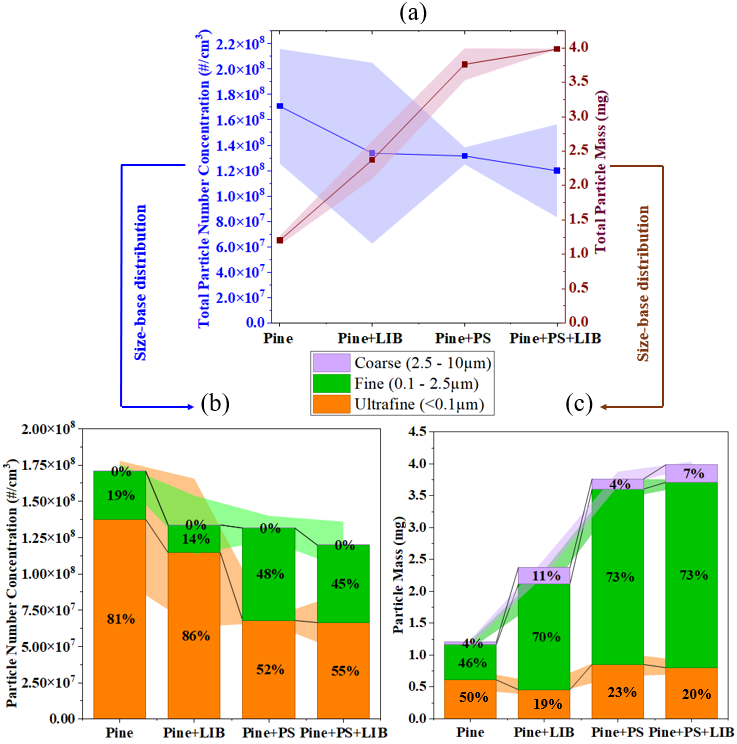


Fig. 3. (a) Total particle number concentration (PNC) and total particle mass (PM); (b) Size-resolved PNC by bin (ultrafine <0.1 µm, fine 0.1–2.5 µm, coarse 2.5–10 µm); (c) Size-resolved PM by bin (ultrafine <0.1 µm, fine 0.1–2.5 µm, coarse 2.5–10 µm) in different fire scenarios.

In the Pine+LIB case, total PNC decreased, yet UFPs remained dominant by number, contributing ~86% of PNC (1.15 × 10⁸ ± 5.10 × 10⁷ #/cm³), while fine particles accounted for ~14% (1.91 × 10⁷ ± 2.02 × 10⁷ #/cm³). However, UFPs represented only approximately 19% of PM (0.46 ± 0.1 mg), whereas fine particles dominated the mass (~70%, 1.66 ± 0.22 mg), and coarse particles contributed approximately 11% (0.26 ± 0.13 mg). This pronounced number–mass decoupling reflects the coupled effects of NMC811 TR at 50% SOC and biomass combustion. During TR in NMC811 LIBs: electrolyte venting and SEI decomposition (SEI ~80–120 °C, anode–electrolyte ~120–180 °C, cathode ~180–250 °C) generate metal-rich vapors and fluorinated/organic gases that initially nucleate as <0.05 µm particles, when co-emitted with biomass-derived VOCs and soot, LIB-derived transition metals act as efficient condensation nuclei and catalytic surfaces, accelerating heterogeneous growth and Brownian coagulation into the accumulation mode (0.1–0.5 µm) within seconds to minutes (Bugryniec et al., 2024; Grossetête et al., 2025; Willstrand et al., 2025). PNC of ~2 × 10^6^ #/cm^3^ and count median diameters shifting from ~.03–.08 µm (early venting) to 0.09–0.18 µm (peak TR) directly matches the observed redistribution (Claassen et al., 2024a; Claassen et al., 2024b; Premnath et al., 2022).

In the Pine+PS case, the particle number distribution shifted substantially from ultrafine to fine modes. UFPs contributed ~52% of PNC (6.80 × 10⁷ ± 1.75 × 10^6^ #/cm³), representing a ~51% reduction relative to Pine UFPs (1.38 × 10⁸ ± 4.04 × 10⁷ #/cm³), while fine particles increased to ~92% (6.36 × 10⁷ ± 8.45 × 10⁶ #/cm³) relative to Pine fine particles (3.32 × 10⁷ ± 6.86 × 10⁶ #/cm³). PM was strongly dominated by fine particles (~73%, 2.76 ± 0.16 mg), with smaller contributions from UFPs (~23%, 0.85 ± 0.18 mg) and coarse particles (~4%, 0.16 ± 0.11 mg). This redistribution reflects aromatic-rich polystyrene pyrolysis (styrene oligomers and PAH precursors), which favors surface growth and coagulation on pre-existing nuclei and soot rather than new particle formation, producing chain-like aggregates predominantly in the 0.15–0.8 µm range even under well-ventilated conditions (Barhoumi et al., 2023; Nobili et al., 2022; Jackson et al., 2025).

In the Pine+PS+LIB configuration, number contributions from UFPs (~55%, 6.65 × 10⁷ ± 2.05 × 10⁷ #/cm³) and fine particles (~45%, 5.34 × 10⁷ ± 1.61 × 10⁷ #/cm³) were more balanced. PM reached its highest level (3.99 ± 0.01 mg), dominated by fine particles (~73%, 2.91 ± 0.04 mg), followed by UFPs (~20%, 0.8 ± 0.1 mg) and coarse particles (~7%, 0.24 ± 0.09 mg). This distribution reflects synergistic effects of LIB vapor/metal nucleation bursts and PS-derived aromatic condensation promoting rapid growth into 0.1–1 µm metal-organic-soot hybrids closely mirroring real-world WUI plumes where biomass, plastics, and engineered materials (including EV batteries) co-burn (Holder et al., 2023; National Academies of Sciences, Engineering, and Medicine, 2022; Willstrand et al., 2025).

## 3.2 Size-resolved particle number and mass distributions

Building on the aggregated PNC and PM behavior, the particle size distributions (PSDs) in Fig. 4 illustrate how the four scenarios differ across the full aerodynamic range (0.0115–9.016 µm for PNC and 0.016–10 µm for PM mass). Fig. 4a shows that all cases exhibit strong nucleation-mode peaks (<0.1 µm) with exponentially decaying PNC toward larger sizes, while Fig. 4b reveals how PM mass shifts from sub-0.1 µm to accumulation-mode diameters (0.1–1 µm) when LIB and PS are present (see supplementary section Table S.3.1 for PM and S.3.2 for PNC detailed data across each size).

In the Pine case, PNC peaks at 2.76 × 10⁷ ± 1.31 × 10⁷ #/cm³ at 0.0154 µm, accounting for ~16% of total PNC, before rapidly declining to <10³ #/cm³ beyond ~1 µm. PM mass reaches its maximum at 0.37 ± 0.12 mg at 0.094 µm (~31% of the total PM), with substantial additional contributions between 0.05 and 0.25 µm (0.205–0.320 mg per stage, collectively accounting for ~40–45% of the total PM). This narrow, nucleation-skewed PSD is consistent with recent wildfire plume characterizations where pine and mixed conifers produce abundant sub-0.1 µm particles via rapid oxidation of biogenic volatiles (including terpenes) to low-volatility vapors, with limited growth into the accumulation mode in the absence of strong inorganic or anthropogenic influences (Ansmann et al., 2021; Shrivastava et al., 2024).


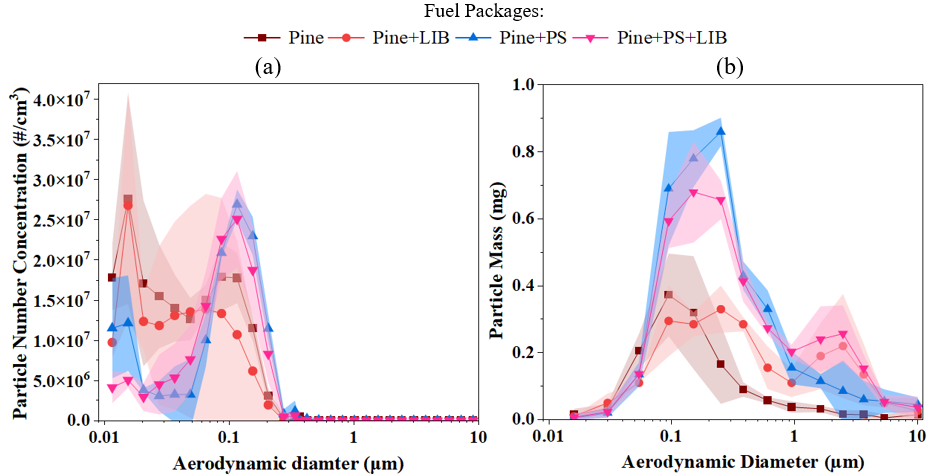


Fig. 4. Particle size distribution by (a) Particle number concentration; (b) Particle mass in different fire scenarios.

In the Pine+LIB case, PNC remains dominated by UFPs, with a nucleation-mode peak of 2.68 × 10⁷ ± 1.42 × 10⁷ #/cm³ at 0.0154 µm, accounting for ~20% of total PNC, followed by a gradual decay toward larger diameters. In contrast to the particle number, PM mass is dominated by accumulation-mode particles, with a maximum of 0.33 ± 0.07 mg at 0.25 µm, collectively accounting for ~15–20% of total PM, while sub-0.1 µm particles contribute minimally. These features arise from TR-induced vapor bursts: fluoride–phosphate clusters from electrolyte decomposition nucleate at <0.05 µm and then grow via condensation of metal oxides (e.g., Co₃O₄, NiO), producing bimodal PSDs (0.02–0.08 µm and 0.2–0.5 µm) similar to those reported in controlled battery fire experiments (Claassen et al., 2024a; Claassen et al., 2024b; Premnath et al., 2022).

In the Pine+PS case, PNC shifts toward larger sizes. The nucleation-mode peak occurs at 0.0866 µm with 2.09 × 10⁷ ± 5.60 × 10^5^ #/cm³, contributing ~16% of total PNC, a decrease of ~24% relative to the Pine only case at nucleation-mode peak from 2.76 × 10⁷ ± 1.31 × 10⁷#/cm³ at 0.0154 µm. In the Pine+PS case, accumulation-mode PNC peaks at 0.1155 µm with 2.69 × 10⁷ ± 1.89 × 10^6^ #/cm³, representing ~20% of total PNC, an increase of ~51% compared to Pine only case from 1.78 × 10⁷ ± 3.14 × 10^6^ #/cm³ at the same size bin. PM mass exhibits a pronounced accumulation-mode maximum at 0.15–0.25 µm, peaking with 0.86 ± 0.04 mg at 0.25 µm, corresponding to ~22% of total PM (3.77 ± 0.23 mg). This broadening and shift in modal diameter reflect PS pyrolysis and soot growth: styrene monomers and oligomers condense on existing cores, then evolve through cluster–cluster aggregation into fractal-like aggregates with mass-median diameters of 0.2–0.5 µm, as observed when synthetic materials (including polystyrene foams and vinyls) are co-combusted with biomass (Goldsmith et al., 2025).

In the Pine+PS+LIB case, nucleation-mode PNC at 0.0154 µm decreases to 5.06 × 10⁶ ± 9.52 × 10^5^ #/cm³, ~4% of total PNC, representing a ~82% reduction relative to the Pine only case (2.76 × 10⁷ ± 1.31 × 10⁷ #/cm³) at the same size bin. Accumulation-mode PNC rises to 2.51 × 10⁷ ± 5.98 × 10⁶ #/cm³ at 0.1155 µm, ~21% of total PNC. PM mass peaks at 0.15 µm with 0.68 ± 0.15 mg, accounting for ~17% of total mass, with a minor coarse-mode contribution between 2.5–10 µm (0.03–0.15 mg, ~7% of total PM). LIB-derived trace elements provide abundant condensation nuclei and catalysts for soot ripening, while PS contributes aromatic precursors and carbonaceous mass, together producing a robust accumulation-mode population highly analogous to laboratory-measured WUI emission profiles where vegetation is co-burned with structural synthetic materials such as plastics, insulation foams under high heat flux (Goldsmith et al., 2025).

## 3.3 Integrated PM, trace element, and PAH concentration across size fractions

The integrated PM, trace elements, and PAHs concentration (Fig. 5) quantify how the physical transformations described above translate into chemical loading across the UFP, fine, and coarse fractions.

Total PM concentration (0–10 µm) increases progressively across fire scenarios, from 15.93 ± 0.89 µg/m³ in Pine to 31.39 ± 3.65 µg/m³ in Pine+LIB, 49.80 ± 3.09 µg/m³ in Pine+PS, and 52.71 ± 0.09 µg/m³ in Pine+PS+LIB case (Fig. 5a). In Pine, PM concentration fractions were dominated by UFP (8.07 ± 2.06 µg/m³, 50% of total), followed by fine fraction (7.28 ± 0.85 µg/m³, 46%) and coarse fraction (0.58 ± 0.55 µg/m³, 4%). NMC811 LIB incorporation redistributed PM concentration toward the fine fraction, with UFP decreasing to 6.04 ± 1.27 µg/m³ (19%), fine fraction increasing to 21.91 ± 2.87 µg/m³ (70%), and coarse fraction at 3.44 ± 1.73 µg/m³ (11%).


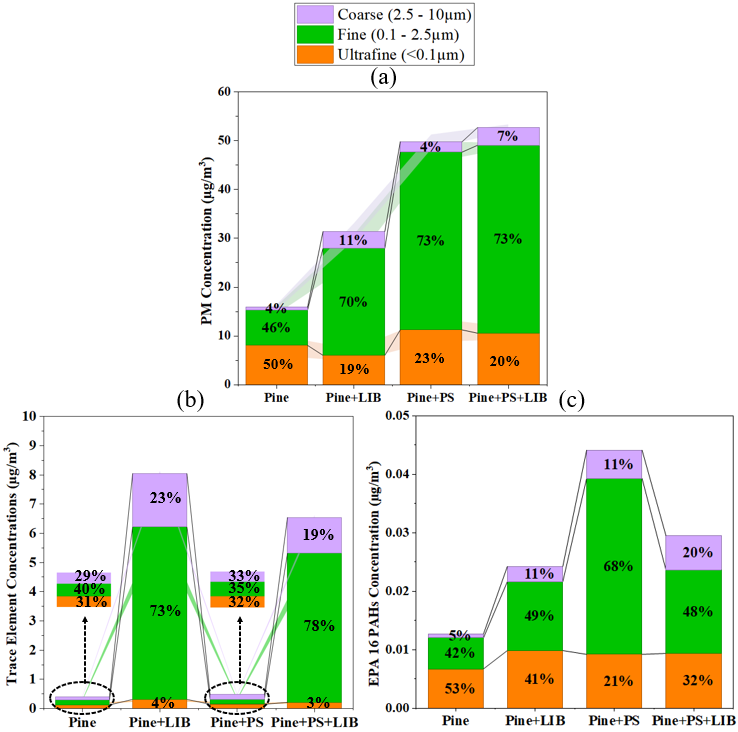


Fig. 5. Total concentration of (a) PM; (b) Metal; and (c) PAH in (ug/m^3^) under different fire scenarios.

Addition of synthetic material PS with Pine produced 11.24 ± 2.43 µg/m³ UFP (23%), 36.44 ± 2.15 µg/m³ fine (73%), and 2.12 ± 1.50 µg/m³ coarse (4%), while the Pine+PS+LIB exhibited 10.58 ± 1.31 µg/m³ UFP (20%), 38.49 ± 0.56 µg/m³ fine (73%), and 3.64 ± 0.65 µg/m³ coarse (7%). These shifts confirm that synthetic PS material converts UFP biomass-derived particles into fine-mode-dominated particulates with larger aerodynamic diameters. Laboratory pine/conifer combustion studies typically report PM₂.₅ concentrations of 10–30 µg/m³ under flaming conditions (Akagi et al., 2011) and wildfire PM_2.5_ exceeds 15 μg/m³ for at least two or three consecutive days from fire (Casey et al., 2024; Jiao et al., 2025), whereas biomass–synthetic material or WUI mixtures yield much higher due to enhanced soot and secondary particulate formation (Goldsmith et al., 2025).

Mechanistically, terpenoid volatilization in Pine drives nucleation of UFP-dominated PM concentration (8.07 ± 2.06 µg/m³, 50% of total), whereas NMC811 LIB TR in Pine+PS moves concentration into the fine fraction (21.91 ± 2.87 µg/m³, +201% vs Pine) via coagulation and condensation of electrolyte-derived vapors (including phosphates and carbonaceous fragments), producing fine-dominated distributions (Claassen et al., 2024a; Claassen et al.,2024b; Premnath et al., 2022). Synthetic material-PS pyrolysis in Pine+PS further increases the fine fraction to 36.44 ± 2.15 µg/m³ (+400% vs Pine) through cyclodehydrogenation and aggregation of aromatics into 0.1 µm aggregates (Eriksson et al., 2014; Li et al., 2021). In Pine+PS+LIB case, fine fraction reaches 38.49 ± 0.56 µg/m³ (+428% vs Pine), reflecting metal–hydrocarbon interactions and rapid accretion of UFP nuclei into the accumulation mode, consistent with enhanced soot yields and partial catalytic oxidation in mixed synthetic material-vegetation burns (Buston et al., 2023; Goldsmith et al., 2025).

Total trace element concentration (Fig. 5b) increased markedly in scenarios involving the NMC811 LIB. Total trace element concentration rose from 0.41 ± 0.05 µg/m³ in Pine to 8.05 ± 0.39 µg/m³ in Pine+LIB, remained low in Pine+PS without NMC811 LIB (0.48 ± 0.05 µg/m³), and reached 6.54 ± 0.39 µg/m³ in Pine+PS+LIB case. In Pine, metals were relatively low across all size fractions (UFP 0.13 ± 0.01 µg/m³, 31%; fine 0.16 ± 0.02 µg/m³, 40%; coarse 0.12 ± 0.01 µg/m³, 29%). Incorporation of NMC811 LIB strongly enhanced fine-mode dominance: in Pine+LIB (UFP 0.32 ± 0.03 µg/m³, 4%; fine 5.90 ± 0.28 µg/m³, 73%; coarse 1.82 ± 0.08 µg/m³, 23%), and in Pine+PS+LIB (UFP 0.22 ± 0.03 µg/m³, 3%; fine 5.10 ± 0.28 µg/m³, 78%; coarse 1.23 ± 0.08 µg/m³, 19%). These increases are consistent with the contribution of trace elements released from NMC811 LIBs during TR, which act as condensation nuclei and favor the formation of fine accumulation-mode particles. These values are consistent with battery-fire studies reporting 5–15 µg/m³ total elements (predominantly Ni, Co, Mn, Al, Cu, Fe as oxides or fluorides) with >70–90% in the fine/accumulation mode (Buston et al., 2023; Claassen et al., 2024a; Claassen et al., 2024b; Premnath et al., 2022). Detailed concentrations and standard deviations for all 44 trace elements from triplicate tests are provided in supplemental Tables S.3.3 and S.3.4. Also, dominant battery-derived elements and their source attribution are provided in Section 3.4.

Under the fixed experimental conditions (50 kW/m² heat flux; 20.95% O₂) employed in this study, total Σ16 EPA PAH concentrations increased from Pine (0.013 µg/m³) to Pine+LIB (0.024 µg/m³), Pine+PS (0.044 µg/m³), and Pine+PS+LIB (0.029 µg/m³ (Fig. 5c). In the pure Pine case, PAHs were dominated by the ultrafine fraction (53%), followed by the fine (42%) and coarse (5%) fractions, consistent with biomass-derived LMW PAHs condensing primarily onto sub-0.1µm particles. Incorporation of the NMC811 LIB with Pine redistributed PAHs toward larger sizes, reducing the UFP contribution to 41% while increasing the fine fraction to 49% and coarse fraction to 11%, indicating enhanced condensation and growth of PAHs under LIB-driven high-temperature conditions.

The Pine+PS scenario produced the highest Σ16 EPA PAH burden and exhibited strong fine-mode dominance (68%), with a substantially reduced UFP contribution (21%), reflecting the efficient formation and growth of aromatic species from synthetic polymer pyrolysis. In the Pine+PS+LIB case, Σ16 EPA PAH remained elevated relative to Pine, with a more balanced size distribution (UFP 32%, fine 48%, coarse 20%), suggesting concurrent PAH formation, metal-assisted condensation, and oxidative fragmentation under extreme mixed-fuel combustion. These results represent a single-condition, exploratory assessment; recent work demonstrates that variations in burn environment alone can drive PAH emission variability of up to ~77%, comparable to or exceeding fuel-type effects (Töpperwien et al., 2025). The observed trends are nonetheless consistent with prior reports showing that LIB fires generate metal-rich soot and ultrafine particles capable of stabilizing PAHs, while polymer-rich fuels promote fine-mode PAH accumulation (Barone et al., 2021; Napolitano et al., 2025). A detailed congener-specific and size-resolved discussion of PAH formation mechanisms and carcinogenicity is provided in Section 3.5.

## 3.4 Dominant battery-derived elements and source attribution

Incorporation of an NMC811 LIB in Pine and Pine+PS markedly increased concentration and altered the distribution of particulate trace elements relative to Pine combustion alone (Fig. 5b). Total trace-element concentration rose from 0.41 ± 0.05 µg/m³ in Pine to 8.05 ± 0.39 µg/m³ in Pine+LIB and 6.54 ± 0.39 µg/m³ in Pine+PS+LIB, whereas Pine+PS without battery involvement remained low (0.48 ± 0.05 µg/m³). These values represent time-integrated averages from triplicate experiments; individual element concentrations resolved by size fraction are reported in Fig. 6 (in ng/m³ for individual elements to facilitate clearer visualization of lower-concentration elements without excessive decimal places). Size-resolved fractional analysis of all 44 trace elements in supplemental Tables S.3.3–S.3.5 provides further quantitative support for battery-dominated source attribution in Pine+LIB and Pine+PS+LIB scenarios.


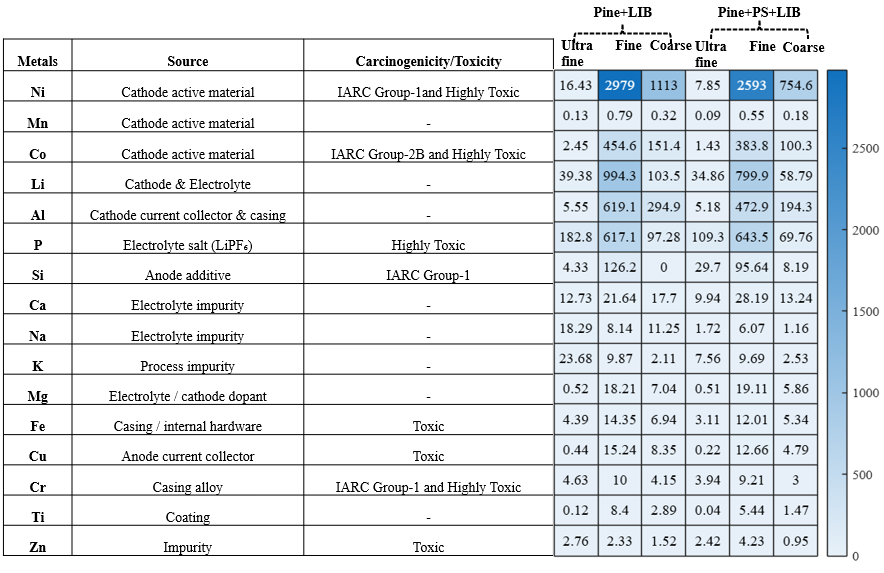


Fig. 6. Dominant trace elements carcinogenicity and their concentration (ng/m^3^) in different fire scenarios.

The primary battery-derived and minor trace elements were Ni, Mn, Co, Li, Al, P, and Si. In Pine+LIB, the ultrafine fraction was chemically distinct and strongly enriched in electrolyte-derived P (182.8 ± 15.2 ng/m³, 57.14 % of UFP trace-element mass), followed by smaller contributions from Ni (16.4 ± 0.6 ng/m³, 5.13 %) and Al (5.6 ± 0.5 ng/m³, 1.7 %). In contrast, the fine fraction was overwhelmingly dominated by cathode-derived Ni (2979 ± 105 ng/m³, 50.5 %), with substantial contributions from phosphorus (617 ± 49 ng/m³, 10.45%), Li (994 ± 49 ng/m³, 16.84 %), Al (619 ± 35 ng/m³, 10.5 %), and Co (455 ± 18 ng/m³, 7.7 %), confirming that the majority of battery-associated trace elements reside in the accumulation mode. A similar pattern persisted in the coarse fraction, where Ni alone accounted for 61.0 % of trace-element mass (1113 ± 39 ng/m³), indicating physical ejection and fragmentation of cathode materials (Yan and Ezekoye, 2023; Yan et al., 2021; Barone et al., 2021; Napolitano et al., 2025).

In the Pine+PS+LIB scenario, PS co-combustion did not alter the elemental hierarchy but modestly redistributed fractional contributions across size modes. Ni remained dominant in the fine (2593 ± 92 ng/m³, 50.9 %) and coarse (755 ± 27 ng/m³, 61.6 %) fractions, while P continued to dominate the ultrafine fraction (109 ± 10 ng/m³, 50 %), emphasizing that battery chemistry—not polymer co-combustion—governs trace-element identity (Yan and Ezekoye, 2023; Yan et al., 2021; Buston et al., 2023; Goldsmith et al., 2025). Li (800 ± 39 ng/m³, 15.9%) and Al (473 ± 27 ng/m³, 9.3%) in the fine fraction reflect the combined effects of electrolyte decomposition and current-collector fragmentation. Mn was consistently detected in all fractions but contributed minimally (~0.07 % of total concentration, e.g., in the range of 0.81-1.24 ng/m³ in Pine+LIB and Pine+PS+LIB), consistent with its lower aerosolization efficiency and stronger lattice retention in NMC811 cathodes (Yan and Ezekoye, 2023; Yan et al., 2021; Barone et al., 2021; Napolitano et al., 2025).

Across battery-containing scenarios, trace elements were strongly enriched in the fine fraction, accounting for ~73 % (Pine+LIB) and ~78 % (Pine+PS+LIB) of total trace-element concentration. Ultrafine particles contributed only ~3–4 % of total trace element concentration but exhibited a chemically distinct signature dominated by electrolyte-derived P species. In Pine+LIB, P in the UFP fraction reached 182.8 ± 15.2 ng/m³ (~57 % of UFP trace-element mass), with a comparable enrichment (~50 %) in Pine+PS+LIB, consistent with thermal decomposition of LiPF₆-based electrolytes and nucleation of phosphate- and fluoride-containing species (Yan and Ezekoye, 2023; Yan et al., 2021; Premnath et al., 2022; Claassen et al., 2024a).

The size-resolved trace-element distributions reported here provide the quantitative basis for the metal-anchored soot morphologies observed in Section 3.6 and inform the subsequent toxicological discussion (Sections 3.7–3.8).

## 3.5 PAH profiles and carcinogenicity across fire scenarios

The size-resolved concentrations of the 16 U.S. EPA priority PAHs across the four experimental fire scenarios are illustrated in Fig.7 and listed in supplemental Table S.3.6, with values reported in ng/m³ to facilitate visualization of both low- and high-abundance compounds in UFPs (<0.1 µm), fine particles (0.1–2.5 µm), and coarse particles (>2.5 µm). Percentage contributions of individual PAHs to the total summed PAH (ΣPAH) for each particle mode are also provided in supplemental Table S3.7.


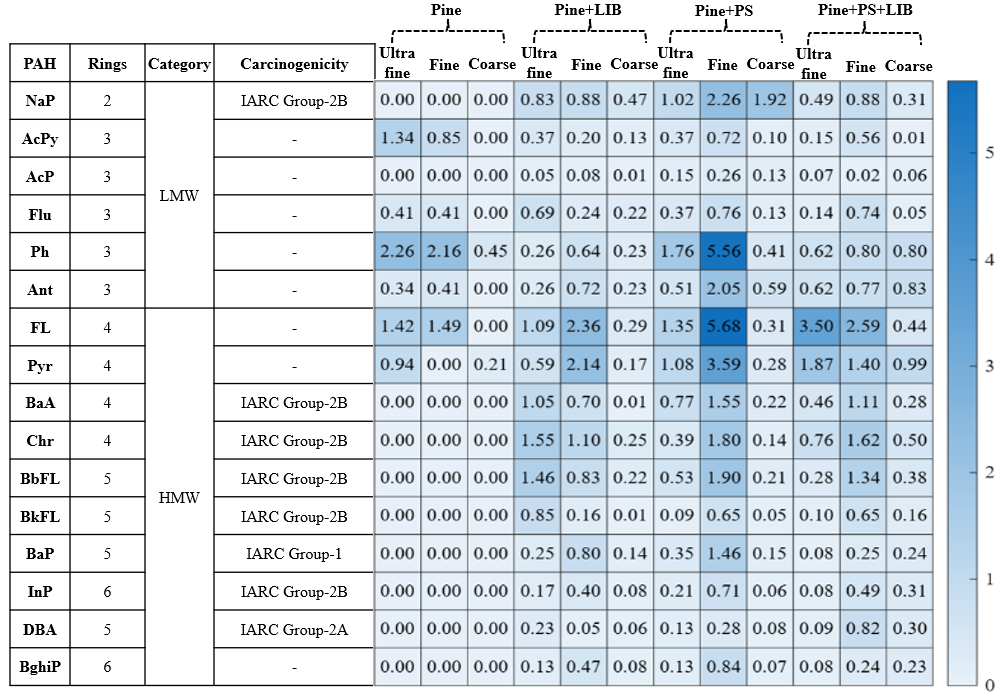


Fig.7. PAH carcinogenicity and their concentration (ng/m^3^) in different fire scenarios.

In the Pine only scenario, ΣPAH reached 12.69 ng/m³, with emissions dominated by LMW (2–3 ring) PAHs. Phenanthrene (Ph) was the most abundant compound (2.26 ng/m³ in ultrafine, 2.16 ng/m³ in fine, and 0.45 ng/m³ in coarse particles), contributing 33.7–68.5% of ΣPAH depending on particle size, followed by fluoranthene (FL; 1.42–1.49 ng/m³, 21.1–28.1%) and pyrene (Pyr; up to 0.94 ng/m³, 14.0–31.5%). Ultrafine particles accounted for 53% of total ΣPAH, and no HMW (5–6 ring) PAHs such as chrysene (Chr), benzo[a]pyrene (BaP), or indeno[1,2,3-cd]pyrene (InP) were detected. This PAH profile is characteristic of well-ventilated softwood combustion, which preferentially forms LMW PAHs while suppressing HMW species (Jenkins et al., 1996; Fine et al., 2001; Vicente and Alves, 2018; Kortelainen et al., 2018).

The addition of an NMC811 LIB in 50% SOC, along with Pine, elevated ΣPAH to 24.23 ng/m³, representing an approximate 2-fold increase compared to the Pine case. UFPs served as the second reservoir for total ΣPAH (~41%) after fine particles served as the primary reservoir for total ΣPAH (~49%). Ultrafine fraction exhibited enrichment with HMW, International Agency for Research on Cancer (IARC)-classified carcinogenic PAHs, such as chrysene (Chr, 1.55 ng/m³, 15.7% of mode-specific ΣPAH), benzo[b]fluoranthene (BbFL, 1.46 ng/m³, 14.8%), and benzo[a]pyrene (BaP, 0.25 ng/m³, 2.57%). Notably, LMW PAHs in Pine+LIB case showed reduced fractional contributions compared to the Pine case, indicative of enhanced aromatization pathways catalyzed by metal-containing particles; however, it is important to clarify that not all LIB TR result in fires, though in this experiment, the 50% SOC condition of NMC811 led to TR and fire (Barone et al. 2021, Claassen et al., 2024a; Claassen et al., 2024b; Wang et al., 2025). Previous studies have reported metal-soot hybrid particles enriched with carcinogenic metals (e.g., nickel, cobalt) from standalone LIB fires (Barone et al. 2021; Claassen et al., 2024a; Napolitano et al. 2025). Particles ejected from LIB fires can have PAHs (Claassen et al., 2024a) in such as well as from biomass combustion (Eriksson et al. 2014; Samburova et al. 2016).

Incorporating 5 wt.% PS along with Pine for the Pine+PS case further increased ΣPAH to 44.12 ng/m³, a roughly 3.5-fold rise compared to the pine baseline and the highest among all scenarios. Both fine- and ultrafine-mode PAHs showed substantial HMW PAH enhancements, with fine-mode PAHs accounting for 68% of total ΣPAH. Ultrafine particles accounted for 21% of total ΣPAH, including detectable HMW PAHs such as benzo[a]anthracene (BaA), Chr, BbFL, and BaP at levels ranging from 0.39 to 1.35 ng/m³. This elevation is consistent with literature on styrene polymer combustion, which promotes HMW PAH production via depolymerization and oligomerization at 400–700 °C, differing from lignocellulosic fuel profiles (Islam et al. 2022; Kortelainen et al. 2018; Sekimoto et al. 2018; Saggese et al. 2013; Zhang et al. 2025).

When NMC811 LIB was co-combusted with both Pine and PS (Pine+PS+LIB), ΣPAH reached 29.51 ng/m³, approximately 2.3 times higher than the pine baseline but lower than the Pine+PS case. Fine particles remained the dominant PAH reservoir (14.27 ng/m³, ~48%), while ultrafine particles contributed a substantial fraction (9.36 ng/m³, ~32%). The ultrafine mode exhibited the strongest relative enrichment of carcinogenic HMW PAHs, including Chr (0.76 ng/m³, 8.08%). Fine-mode PAHs were dominated by fluoranthene, pyrene, and phenanthrene, together contributing approximately 30% of fine-mode ΣPAH, while the coarse fraction carried a comparatively minor PAH load (20%), indicating limited partitioning onto larger particles.

Across all scenarios, ΣPAH followed the progression of Pine (12.7 ng/m³) < Pine+LIB (24.2 ng/m³) < Pine+PS+LIB (29.5 ng/m³) < Pine+PS (44.1 ng/m³), with UFPs enrichment of HMW PAHs peaking in the Pine+PS+LIB case due to the combined effects of LIB metals and synthetic fuel pyrolysis. Fine particles consistently dominated total ΣPAH, reflecting aerosol aggregation and growth. Töpperwien et al. (2025) reported up to 77% variability in PAH phenanthrene/anthracene emissions, as these are highly dependent on combustion conditions, including temperature, oxygen availability, fuel moisture content, and flaming versus smoldering phases. While the PAH analysis in this study was limited to a single test configuration, future research should explore variations in temperature, oxygen availability, fuel moisture content, and flaming versus smoldering phases to better quantify PAH variability and enhance predictive models for real-world wildfire scenarios.

## 3.6 Metal–Soot morphologies and implications for particle evolution

SEM and EDS mapping (Fig. 8) provide direct mechanistic insight into how battery-derived metals restructure Pine soot, complementing the size-segregated chemical signatures described in Section 3.4. In the Pine+LIB scenario, low magnification (1500×; Fig. 8a) reveals densely packed filter surfaces containing bright, spherical-to-sub-spherical inclusions embedded within fibrous soot networks. At higher magnification (10,000–15,000×; Fig. 8b–d), these inclusions appear as consolidated 0.2 – 0.8 µm agglomerates consisting of metal-rich cores enveloped by carbonaceous shells. EDS elemental mapping (see supplemental Figure S.3.2 - 3.5) and ICP-MS analysis confirm substantial enrichment of Ni, Co, P, Al, Fe—precisely the dominant metals identified in Section 3.4 indicating that these species act as nucleation centers around which soot and semi-volatile organics condense and reorganize during plume cooling (Claassen et al., 2024a; Premnath et al., 2022; Tian et al., 2025).


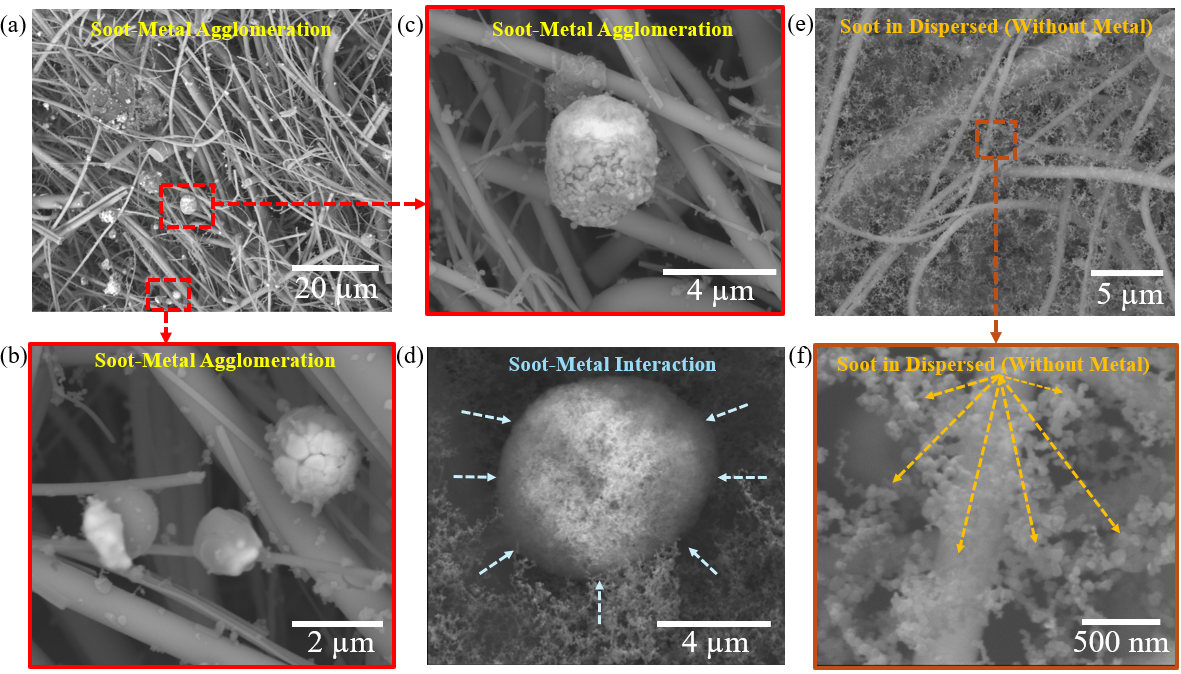


Fig.8. Soot and metal morphologies, interaction, and agglomeration at magnification of (a) Metals with soot at 1500×; (b) Soot-Metal agglomeration at 15000×; (c) Soot-Metal agglomeration at 10000×; (d) Soot and metal interaction at 10000×; (e) Soot in dispersed without metal interaction at 5000×; (f) Soot in dispersed without metal interaction at 50000×.

Without metal interaction, Pine soot (Fig. 8e–f) appears as dispersed primary spherules, 0.02 to 0.04 µm in diameter, forming loose fractal aggregates with minimal compaction. These morphologies match classical descriptions of coniferous flaming soot, where aggregation proceeds through weak van der Waals cohesion in the near absence of inorganic inclusions (Chakrabarty et al., 2014; Elsner et al., 2025; Pang et al., 2023; Vicente & Alves, 2018). The lack of bright nucleation centers or welded clusters in Figs. 8e–f underscores the structural transformations in Figs. 8a–d is uniquely driven by the presence of battery metals during TR.

The transition from porous, fractal soot to persistent hybrid metal–soot agglomerates arises through three concurrent physicochemical pathways activated during NMC battery failure. First, hetero-coagulation is strongly enhanced by the surface charge characteristics of transition-metal oxides such as NiO and Co₃O₄, which carry zeta (ζ)-potentials following cathode degradation (Claassen et al., 2024a; Claassen et al., 2024b; Premnath et al., 2022). Although Pine soot is also negatively charged, its surface exhibits patch-wise charge heterogeneity, allowing oppositely charged microdomains to facilitate attachment of metal nanoparticles via electrostatic bridging (Elsner et al., 2025; Pang et al., 2023; Vicente & Alves, 2018). This mechanism promotes rapid metal–soot association during the early cooling phase of the plume.

Second, the adsorption of metal nanoparticles substantially increases soot surface roughness and asperity density, amplifying van der Waals forces by raising the effective Hamaker constant (Elsner et al., 2025). This enhanced short-range adhesion stabilizes multi-particle junctions, resulting in the compact, grape-like agglomerates observed in Fig. 8b–c. These structures resist disintegration during sampling, consistent with other studies that show mixed metal–soot aggregates form within seconds in high-temperature combustion environments (Jahn et al., 2021; Ma, 2013).

Third, catalytic graphitization and partial sintering occur at metal–carbon interfaces. Transition metals such as Ni and Co are well-established catalysts for dehydrogenation, carbonization, and graphitization at temperatures ranging from 400 to 600 °C (Chen et al., 2018; Kim et al., 2020; Pudikov et al., 2018). These reactions lower activation barriers for C–C bond formation, generating thickened carbon shells and more ordered microstructures (Chen et al., 2018; Kim et al., 2020; Jahn et al., 2021). The consolidated, layered morphologies observed in Fig. 8d are consistent with such catalytic restructuring—features that are completely absent in soot without metal interaction (Fig. 8e and f).

These morphological pathways provide a coherent mechanistic link to the size-segregated metal and PAH results from earlier sections. The dominance of Ni, Co, Al, and Li in the fine mode (Section 3.4) reflects their role as metallic scaffolds that accrete soot and organics into submicron particles rather than remaining as isolated UFP nuclei (Claassen et al., 2024a; Claassen et al., 2024b). Conversely, the high abundance of P in the UFP fraction matches the numerous <.05 µm embedded nanoparticles visible in Figs. 8b–d, consistent with nucleation of LiPF₆-derived phosphate/fluoride clusters described in lithium-ion fire literature (Claassen et al., 2024a; Premnath et al., 2022; Tian et al., 2025). The pronounced enrichment of HMW PAHs in the UFP and fine modes (Section 3.5) further corresponds with the expanded surface area and strong adsorption affinity of these metal-decorated soot surfaces (Jahn et al., 2021; Ma, 2013).

Overall, these observations demonstrate that battery involvement does not simply increase metal emissions; it fundamentally reorganizes soot into metal-welded hybrid particles with greatly enhanced capacity to concentrate redox-active metals and carcinogenic PAHs in the respirable size range. These newly formed hybrids underpin the atmospheric persistence (Section 3.7) and the toxicological implications for inhalation exposure (Section 3.8), marking a profound shift from traditional Pine smoke toward a more hazardous emission class unique to battery-involved scenarios.

To concisely illustrate this emergent class distinction, Table 2 summarizes key particle modes, dominant metals, and HMW PAH enrichments across scenarios, highlighting the systematic progression toward metal–PAH hybrids in battery- and polymer-involved fires

Table 2. Summary of particle characteristics defining a new class of Soot–Metal–PAH hybrid particles

| Fuel package representation with weight percentile (%) | Total PNC (#/cm³ ± SD)/UFP % | Total PM concentration (µg/m³ ± SD)/ UFP % | Total trace element concentration (µg/m³ ± SD) / (Total UFP% / Fine%; Dominant elements UFP%/ Fine%) | Total PAH concentration (µg/m³ ± SD)/(HMW % in UFP/ Fine; Dominant PAH% in UFP) |
| --- | --- | --- | --- | --- |
| Pure biomass  (Pine 100 wt.%) | 1.71 × 10⁸ ± 4.51 × 10⁷/81% | 15.93 ± 0.89/ 50% | 0.41 ± 0.05 µg/m³/ (Total UFP 31%/Fine 40%; individual minimal) | ΣPAH 12.69 ng/m³, HMW% in UFP/Fine: 35%/28% (None detected in 5-6 ring carcinogens) |
| LIB involved biomass  (Pine 66 wt.% + NMC811 34 wt.%) | 1.34 × 10⁸ ± 7.12 × 10^7^/86% | 31.39 ± 3.65/ 19% | 8.05 ± 0.39 µg/m³/ (Total UFP 4%/Fine 73%; Ni 5.13%/ 50.5%, P 57.14%/ 10.45%, Li 12.31%/ 16.84%) | ΣPAH 24.23 ng/m³, HMW % in UFP/Fine: 75% / 77% (Chr 1.55 ng/m³, 15.7%; BbFL 1.46 ng/m³;14.8%; BaP 0.25 ng/m³; 2.6% in UFP) |
| Biomass with synthetic material  (Pine 95 wt.% + PS 5 wt.%) | 1.32 × 10⁸ ± 6.71 × 10^6^/52% | 49.80 ± 3.09/ 23% | 0.48 ± 0.05 µg/m³ (Total UFP 32%/Fine 35%; individual minimal) | ΣPAH 44.12 ng/m³, HMW% in UFP/Fine: 55% / 61% (FL 1.35 ng/m³, 14.67%; Pyr 1.08 ng/m³, 11.92%; BaP 0.35 ng/m³, 3.84% in UFP) |
| LIB involved biomass and synthetic material  (Pine 61 wt.% + PS 5 wt.% + NMC811 34 wt.%) | 1.20 × 10⁸ ± 3.66 × 10⁷/55% | 52.71 ± 0.09/ 20% | 6.54 ± 0.39 µg/m³ (Total UFP 3%/Fine 78%; Ni 3.58%/ 50.9%, P 50%/12.62%, Li 15.88/15.9%) | ΣPAH 29.51 ng/m³, HMW% in UFP/Fine: 78% / 74% (FL 3.50 ng/m³, 37.39%; Pyr 1.87 ng/m³, 19.95%; BaP 0.08 ng/m³, 0.81% in UFP) |

3.7 Environmental hazard perspective of battery- and polymer-involved fire emissions

This study employed a controlled combustion chamber to quantify particulate emissions under reproducible, near-source conditions representative of short-term, high-intensity exposures. Each experimental fire was conducted for a fixed duration of 30 minutes, allowing for the time-integrated characterization of particle mass, number, size distribution, and chemical composition during active combustion. Real-time particle number and size distributions spanning 0.011–10 µm were measured using a SMPS (0.011–0.3 µm) and an OPS (0.3–10 µm), both operated with a constant 1:100 precision dilution to maintain concentrations within the instrument's operating limits. PNC reported here were normalized to account for this dilution, whereas gravimetric and chemical analyses were performed on PM collected directly from the raw exhaust stream without dilution. As such, all reported concentrations represent raw duct values under controlled laboratory conditions and should be interpreted as hazard indicators for elevated short-term particulate burdens in near-source plumes, rather than as direct surrogates for ambient air quality metrics.

Although WUI fires typically occur in open environments, this chamber-based configuration is particularly relevant for understanding acute exposure scenarios experienced by firefighters and first responders. These populations frequently operate in close proximity to flame fronts, within structural interiors, vehicle compartments, or other partially enclosed microenvironments where smoke concentrations can substantially exceed far-field ambient levels, affecting the general population (National Academies of Sciences, Engineering, and Medicine, 2022; Rumi et al., 2025). Accordingly, direct comparisons with regulatory ambient guidelines are not appropriate due to differences in averaging time, dilution, and exposure duration; instead, the data provide insight into the relative hazard potential of different fuel combinations under identical combustion conditions.

Across the four fuel configurations, total PM increased systematically with increasing material complexity, from 15.93 ± 0.89 µg/m³ for pure Pine combustion to 31.39 ± 3.65 µg/m³ for Pine+LIB, 49.80 ± 3.08 µg/m³ for Pine+PS, and 52.71 ± 0.09 µg/m³ for Pine+PS+LIB. These values represent chamber-averaged concentrations integrated over the active combustion and sampling period and demonstrate the strong capacity of battery- and polymer-containing fuels to amplify particulate loading under short-duration, near-source conditions.

Battery involvement produced a particularly pronounced enhancement in particulate trace-element burdens. Total trace-element concentrations increased from 0.41 ± 0.05 µg/m³ in Pine to 8.05 ± 0.39 µg/m³ in Pine+LIB and 6.54 ± 0.39 µg/m³ in Pine+PS+LIB, while remaining comparatively low in Pine+PS (0.48 ± 0.05 µg/m³). Emissions were dominated by Ni, Li, P, Co, and Al, with more than 70% of the elemental mass consistently partitioning into the fine (0.1–2.5 µm) fraction. While not intended for regulatory comparison, qualitative benchmarking highlights the potential hazard posed by these emissions, underscoring the severity of short-term, near-field exposure scenarios for firefighters and first responders (European Environment Agency, 2020; National Academies of Sciences, Engineering, and Medicine, 2022; Rumi et al., 2025).

PAH burdens exhibited a similar progression, with Σ16 EPA PAHs increasing from 0.013 µg/m³ in Pine to 0.024 µg/m³ in Pine+LIB, 0.044 µg/m³ in Pine+PS, and 0.029 µg/m³ in Pine+PS+LIB. Although these concentrations are lower than those reported for some large-scale wildfire plumes, battery- and polymer-containing scenarios showed clear enrichment of HMW PAHs within ultrafine and fine particles. BaP, undetectable in pure Pine emissions, was consistently present in LIB- and PS-containing cases at sub- to low-ng/m³ levels, approaching or exceeding values commonly used as long-term ambient indicators when considered on a short-term exposure basis (European Environment Agency, 2020).

Across all scenarios, UFPs accounted for 52–86% of the total PNC, while contributing only 19–23% of the PM mass in battery- and polymer-involved cases. This pronounced number–mass decoupling indicates that chemically enriched, toxicologically relevant constituents are disproportionately associated with the smallest particles. Collectively, these findings suggest that emissions from pure biomass, such as Pine-only combustion, may substantially underestimate the hazard potential of modern WUI and battery-involved fires, particularly with respect to metal-rich and PAH-laden submicron aerosols (National Academies of Sciences, Engineering, and Medicine, 2022; UNEP, 2022).

## 3.8 Health hazard perspective: ultrafine carriers, carcinogenicity, and acute toxicological implications

From a public health standpoint, the dominant concern associated with battery- and polymer-involved fire emissions lies not solely in increased PM mass, but in the co-localization of UFPs with carcinogenic metals and PAHs. UFPs (<0.1 µm) possess high surface area–to–mass ratios, enabling deep penetration into the alveolar region and, in some cases, translocation beyond the pulmonary system (Corsini et al., 2019; Health Effects Institute, 2013; Kwon et al., 2020; Rumi et al., 2025). Their toxicity is therefore driven more strongly by surface chemistry and associated constituents than by mass concentration alone.

In this study, ultrafine and fine particles generated during Pine+LIB and Pine+PS+LIB combustion carried concentrated loads of Ni, Co, P-rich species alongside HMW PAHs such as chr and BaP. These constituents are well-established drivers of oxidative stress, inflammatory signaling, mitochondrial dysfunction, and genotoxicity in pulmonary and cardiovascular tissues (Bukowska et al., 2022; Cassera et al., 2025; IARC, 2023; Rumi et al., 2025). Although the chamber-derived concentrations cannot be directly extrapolated to ambient exposure metrics, their magnitude and chemical complexity are consistent with short-term exposure conditions encountered by firefighters and emergency responders operating near active fire zones.

Evidence from battery-specific toxicological studies further reinforces these concerns (Meister et al.,2025; Zhang et al., 2025). Aerosols emitted during LIB failure and fire events—particularly those involving Ni- and Co-rich cathode chemistries—have been shown to elicit stronger oxidative and cytotoxic responses than carbonaceous particles alone, with ultrafine fractions exhibiting disproportionately high toxicity per unit mass (Barone et al., 2021; Meister et al., 2025).

Epidemiological evidence consistently links wildfire smoke exposure to increased respiratory and cardiovascular morbidity, particularly during short-term PM₂.₅ spikes that may exceed 100 µg/m³ during severe events (Rappold et al., 2012; Downward et al., 2018; Su et al., 2024). While most population-level studies do not resolve particle composition at the level reported here, the present results suggest that a mixed fuel package (pure biomass species like Pine, PS as representation of synthetic materials in urban interface and involving commonly used NMC811 batteries in consumer electronics and EVs), may generate aerosols with toxicological characteristics more closely resembling complex industrial combustion mixtures than traditional biomass smoke. This distinction is especially relevant for occupational exposure scenarios involving firefighters, who may experience repeated acute exposures across a fire season.

Current air quality standards emphasize PM₂.₅ and PM_10_ mass and long-term averages and do not explicitly address ultrafine particle number, metal–PAH co-association, or short-duration exposure peaks characteristic of fireground environments (EPA, 2024; WHO, 2021; Rumi et al., 2025). Recent assessments by the National Academies of Sciences, Engineering, and Medicine (2022) and UNEP (2022) have also highlighted these gaps. The findings presented here support the need for composition-aware hazard frameworks that explicitly consider ultrafine particle chemistry and battery-derived constituents when evaluating health risks associated with contemporary fire events.

# Conclusion

# This study presents the first integrated, size- and composition-resolved assessment of particulate emissions from the co-combustion of biomass, synthetic polymer, and NMC-811 LIB under controlled, WUI-relevant flaming conditions. The results demonstrate a nonlinear and synergistic restructuring of particulate emissions that far exceeds the additive contributions from individual fuels. Whereas pure pine combustion produced predominantly organic UFPs with low PM mass, minimal transition metals, and PAHs dominated by LMW congeners, the inclusion of synthetic polymers, especially a single LIB, fundamentally altered particle size distributions, chemistry, and morphology.

NMC811 LIB involvement induced pronounced number–mass decoupling, with UFPs continuing to dominate particle number while PM mass, trace elements, and PAHs shifted decisively into the fine fraction. Total trace element concentration increased by more than an order of magnitude, dominated by Ni, Li, P, Co, and Al. Microscopic and chemical evidence confirms that NMC811 LIB thermal runaway generates P-rich ultrafine nuclei and Ni/Co-bearing vapors that rapidly coagulate into compact 0.2–0.8 µm metal–soot hybrid particles, concurrently enriching ultrafine and fine fractions with carcinogenic 4–6-ring PAHs (e.g., benzo[a]pyrene, chrysene, benzo[b]fluoranthene).

Although chamber-derived concentrations are not intended for direct regulatory comparison, the observed PM, Ni, and PAH burdens indicate substantially elevated short-term hazard potential under near-source exposure conditions, particularly relevant for firefighters and first responders operating in enclosed or semi-enclosed environments. Mass-based PM₂.₅ metrics and biomass-only emission inventories, therefore, systematically underrepresent the health-relevant toxicity of contemporary WUI and battery-involved fire smoke.

# The broader implications are significant. Current air-quality frameworks do not adequately capture acute toxicity driven by ultrafine, metal- and PAH-laden hybrid particles. Effective public health protection during WUI fires will require particle number measurements, size-resolved chemical speciation, and the explicit inclusion of battery-derived constituents in emission and exposure models. As residential structures, synthetic polymer materials, and LIBs increasingly intersect with wildfire regimes, failure to modernize monitoring and risk-assessment paradigms will perpetuate systematic underestimation of exposure and health risk, leaving vulnerable populations and emergency responders insufficiently protected.

Limitations and Future Directions

This study employed a controlled-chamber combustion framework with a fixed radiant heat flux (50 kW.m²) and normoxic conditions (20.95% O₂) to enable mechanistic, size-resolved comparisons across fuel scenarios. However, real WUI fires involve greater variability in heat flux, oxygen availability, combustion phase, and fuel package composition. Future work will incorporate variable heat flux and ventilation to resolve flaming, mixed, and smoldering regimes. Only one LIB chemistry (NMC811) at 50% SOC was examined; future studies will assess higher SOCs and additional chemistry (e.g., LFP). While PS was used as a representative near-WUI synthetic material, future experiments will expand to PVC and other structural polymers commonly used in residential fuels. PAH analyses were conducted under single test conditions due to resource constraints; replicated testing is planned to quantify variability. Finally, field-scale validation during prescribed burns or real incidents is needed to translate these findings to occupational and public health exposure scenarios.

# Data availability statement

# The raw data supporting the conclusions of this article will be made available by the authors, without undue reservation.

Author contributions

Md Jalal Uddin Rumi: Conceptualization, Methodology, Investigation, Formal analysis, Data curation, Visualization, Writing – original draft.

Yulin Wu: Methodology, Investigation, Data curation, Validation.

Md. Jakir Hossain: Methodology, Investigation.

Mazyar Etemadzadeh: Methodology, Investigation.

Mengying Zhang: Writing – review & editing.

Todd A. Kingston: Conceptualization, Writing – review & editing, Resources, Supervision.

Rui Li: Conceptualization, Writing – review & editing, Resources, Supervision.

Guowen Song: Conceptualization, Resources, Supervision, Funding acquisition, Project administration, Writing – review & editing.

Declaration of Generative AI and AI-assisted Technologies in the Writing Process
During the preparation of this work, the authors used AI-assisted technologies and grammar-checking tools to improve the language and readability of the manuscript. After using these tools/services, the authors reviewed and edited the content as needed, taking full responsibility for the content of the publication.

Funding

The Fire Prevention and Safety (FP&S) Research and Development (R&D) Grant, which is part of the Assistance to Firefighters Grants (AFG) and funded by the U.S. Department of Homeland Security (DHS) through the Federal Emergency Management Agency (FEMA), is acknowledged for its financial support (project numbers EMW-2021-FP-00088, EMW-2023-FP-00242, EMW-2024-FP-00732). FP&S aims to reduce firefighter fatalities and nonfatal injuries and improve the safety, health, and wellness of firefighters.

Abbreviations

AcPy Acenaphthylene

AcP Acenaphthene

Al Aluminium

Ant Anthracene

BaA Benzo[a]anthracene

BaP Benzo[a]pyrene

BbFL Benzo[b]fluoranthene

BghiP Benzo[ghi]perylene

Bi Bismuth

BkFL Benzo[k]fluoranthene

Cd Cadmium

Chr Chrysene

Co Cobalt

Cr Chromium

Cu Copper

DBA Dibenz[a,h]anthracene

DLPI+ Dekati Low Pressure Impactor Plus

EC Ethylene Carbonate

EDS Energy-Dispersive X-ray Spectroscopy

EPA U.S. Environmental Protection Agency

Fe Iron

FE-SEM Field-Emission Scanning Electron Microscopy

FL Fluoranthene

Flu Fluorene

GC–MS/MS Gas Chromatography–Tandem Mass Spectrometry

HCL Hydrochloric Acid (HCl)

HMW High-Molecular-Weight

HNO_3_ Nitric Acid

H_2_O_2_ Hydrogen Peroxide

IARC International Agency for Research on Cancer

ICP–MS Inductively Coupled Plasma–Mass Spectrometry

InP Indeno[1,2,3-cd]pyrene

K Potassium

Li Lithium

LIB(s) Lithium-Ion Battery/Batteries

LMW Low-Molecular-Weight

MCE Modified Combustion Efficiency

Mn Manganese

NaP Naphthalene

Ni Nickel

NMC Nickel–Manganese–Cobalt

OPS Optical Particle Sizer

P Phosphorus

PAH Polycyclic Aromatic Hydrocarbon

PCTE Polycarbonate Track-Etched

Ph Phenanthrene

PM Particulate Matter

PM₂.₅ Fine Particulate Matter (≤ 2.5 µm)

PM₁₀ Coarse Particulate Matter (≤ 10 µm)

PNC Particle Number Concentration

PS Polystyrene

PSD Particle (Number) Size Distribution

Pyr Pyrene

SMPS Scanning Mobility Particle Sizer

SOC State of Charge

TR Thermal Runaway

UFP Ultrafine Particles (< 0.1 µm)

VOC Volatile Organic Compounds

WHO World Health Organization

WUI Wildland–Urban Interface

Acknowledgements

The authors gratefully acknowledge the use of facilities at the Materials Analysis Research Laboratory of Iowa State University for field emission scanning electron microscopy coupled with energy-dispersive X-ray spectroscopy analyses; the Division of Atmospheric Sciences, Desert Research Institute, Reno, Nevada, for inductively coupled plasma-mass spectrometry; and the W.M. Keck Metabolomics Research Lab of Iowa State University for gas chromatography-tandem mass spectrometry. The authors also thank Qusai Alahmad, Ph.D. student in the Department of Mechanical Engineering at Iowa State University, for technical assistance with battery cycling experiments.

Conflict of interest

The authors declare that they have no known competing financial interests or personal relationships that could have appeared to influence the work reported in this paper.

# References

Adanouj, I., Napolitano, E., Sommariva, M., Enzo, S. and Lebedeva, N., 2023. Characterisation of smoke particles from lithium-ion battery fire: Morphology, size and composition. ECS Meeting Abstracts, MA2023-02(2), p.431. <https://doi.org/10.1149/MA2023-022431mtgabs>

Akagi, S.K., Yokelson, R.J., Wiedinmyer, C., Alvarado, M.J., Reid, J.S., Karl, T., Crounse, J.D. and Wennberg, P.O., 2011. Emission factors for open and domestic biomass burning for use in atmospheric models. Atmospheric Chemistry and Physics, 11(9), pp.4039–4072. <https://doi.org/10.5194/acp-11-4039-2011>

Ansmann, A., Ohneiser, K., Mamouri, R.-E., Knopf, D.A., Veselovskii, I., Baars, H., Engelmann, R., Foth, A., Jimenez, C., Seifert, P. and Barja, B., 2021. Tropospheric and stratospheric wildfire smoke profiling with lidar: Mass, surface area, CCN and INP retrieval. Atmospheric Chemistry and Physics, 21(13), pp.9779–9807. <https://doi.org/10.5194/acp-21-9779-2021>

Barhoumi, B., Metian, M., Oberhaensli, F., Mourgkogiannis, N., Karapanagioti, H.K., Bersuder, P. and Tolosa, I., 2023. Extruded polystyrene microplastics as a source of brominated flame retardant additives in the marine environment: Long-term field and laboratory experiments. Environment International, 172, 107797. <https://doi.org/10.1016/j.envint.2023.107797>

Barone, T.L., Dubaniewicz, T.H., Friend, S.A., Zlochower, I.A., Bugarski, A.D. and Rayyan, N.S., 2021. Lithium-ion battery explosion aerosols: Morphology and elemental composition. Aerosol Science and Technology, 55(10), pp.1183–1201. <https://doi.org/10.1080/02786826.2021.1938966>

Buston, J. Gill, R. Lisseman, J. Morton, D. Musgrove and R.C.E. Williams, Experimental determination of metals generated during the thermal failure of lithium ion batteries, Energy Adv., 2023,2, 170-179.

<https://doi.org/10.1039/D2YA00279E>

Bugryniec, P.J., Resendiz, E.G., Nwophoke, S.M., Khanna, S., James, C. and Brown, S.F., 2024. Review of gas emissions from lithium-ion battery thermal runaway failure in enclosures. Journal of Energy Storage, 87, 111288. <https://doi.org/10.1016/j.est.2024.111288>

Bukowska, B., Mokra, K. and Michałowicz, J., 2022. Benzo[a]pyrene – Environmental occurrence, human exposure, and mechanisms of toxicity. International Journal of Molecular Sciences, 23(11), 6348. <https://doi.org/10.3390/ijms23116348>

Cassera, E., Ferrari, E., Vignati, D.A.L. and Capucciati, A., 2025. The interaction between metals and catecholamines: Oxidative stress, DNA damage, and implications for human health. Brain Research Bulletin, 226, 111366. https://doi.org/10.1016/j.brainresbull.2025.111366

Castaño-Ortiz, J.M., Romero, F., Cojoc, L., Barceló, D., Balcázar, J.L., Rodríguez-Mozaz, S. and Santos, L.H.M.L.M., 2024. Accumulation of polyethylene microplastics in river biofilms and effect on the uptake, biotransformation and toxicity of the antimicrobial triclosan. Environmental Pollution, 344, 123369. <https://doi.org/10.1016/j.envpol.2024.123369>

Casey, J.A., Kioumourtzoglou, M.-A., Padula, A., González, D.J.X., Elser, H., Aguilera, R., Northrop, A.J., Tartof, S.Y., Mayeda, E.R., Braun, D., Dominici, F., Eisen, E.A., Morello-Frosch, R. and Benmarhnia, T., 2024. Measuring long-term exposure to wildfire PM₂.₅ in California: Time-varying inequities in environmental burden. Proceedings of the National Academy of Sciences, 121(8), e2306729121. <https://doi.org/10.1073/pnas.2306729121>

Chakrabarty, R.K., Beres, N. and Moosmüller, H., et al., 2014. Soot superaggregates from flaming wildfires and their direct radiative forcing. Scientific Reports, 4, 5508. <https://doi.org/10.1038/srep05508>

Chen, C., Sun, K., Wang, A., Wang, S. and Jiang, J., 2018. Catalytic graphitization of cellulose using nickel as catalyst. BioResources, 13(2), pp.3165–3176. <https://doi.org/10.15376/biores.13.2.3165-3176>

Chen, G., Guo, Y., Yue, X., Tong, S., Gasparrini, A. and Bell, M.L., et al., 2021. Mortality risk attributable to wildfire-related PM₂.₅ pollution: A global time series study in 749 locations. The Lancet Planetary Health, 5(9), pp.e579–e587. https://doi.org/10.1016/S2542-5196(21)00200-X

Chen, H., Buston, J., Gill, J., Howard, D., Williams, R., Vendra, C., Shelke, A. and Wen, J., 2020. An experimental study on thermal runaway characteristics of lithium-ion batteries with high specific energy and prediction of heat release rate. Journal of Power Sources, 472, 228585. <https://doi.org/10.1016/j.jpowsour.2020.228585>

Chen, J., Zhang, Y. and Liu, W., 2023. A review of the physicochemical characteristics of ultrafine particle emissions from domestic solid fuel combustion during cooking and heating. Science of the Total Environment, 879, 163747. <https://doi.org/10.1016/j.scitotenv.2023.163747>

Claassen, M., Bingham, B., Chow, J.C., Watson, J.G., Chu, P., Wang, Y. and Wang, X., 2024a. Characterization of lithium-ion battery fire emissions – Part 1: Chemical composition of fine particles (PM₂.₅). Batteries, 10(9), 301. <https://doi.org/10.3390/batteries10090301>

Claassen, M., Bingham, B., Chow, J.C., Watson, J.G., Wang, Y., Sambamurti, K. and Olatunji, O.T., 2024b. Characterization of lithium-ion battery fire emissions – Part 2: Particle size distributions and emission factors. Batteries, 10(10), 366. <https://doi.org/10.3390/batteries10100366>

Corsini, E., Marinovich, M. and Vecchi, R., 2019. Ultrafine particles from residential biomass combustion: A review on experimental data and toxicological response. International Journal of Molecular Sciences, 20(20), 4992. <https://doi.org/10.3390/ijms20204992>

Davis, A., Cleary, T., Falkenstein-Smith, R. and Bryant, R., 2025. Burning characteristics and smoke emission from mixed fuel cribs. ACS ES&T Air, 2(4), pp.540–547. <https://doi.org/10.1021/acsestair.4c00275>

Downward, G.S., van Nunen, E.J.H.M., Kerckhoffs, J., Vineis, P., Brunekreef, B., Boer, J.M.A., Messier, K.P., Roy, A., Verschuren, W.M.M., van der Schouw, Y.T., Sluijs, I., Gulliver, J., Hoek, G. and Vermeulen, R., 2018. Long-term exposure to ultrafine particles and incidence of cardiovascular and cerebrovascular disease in a prospective study of a Dutch cohort. Environmental Health Perspectives, 126(12), 127007. <https://doi.org/10.1289/EHP3047>

Elsner, F., Gerhards, P., Berrier, G., Vincent, R., Dubourg, S. and Pischinger, S., 2025. Detailed characterization of thermal runaway particle emissions from a prismatic NMC622 lithium-ion battery. Batteries, 11(6), 225. <https://doi.org/10.3390/batteries11060225>

Eriksson, A.C., Nordin, E.Z., Nyström, R., Pettersson, E., Swietlicki, E., Bergvall, C., Westerholm, R., Boman, C. and Pagels, J.H., 2014. Particulate PAH emissions from residential biomass combustion: Time-resolved analysis with aerosol mass spectrometry. Environmental Science & Technology, 48(12), pp.7143–7150. <https://doi.org/10.1021/es500486j>

European Environment Agency, 2020. Air quality in Europe – 2020 report (EEA Report No. 09/2020). Luxembourg: Publications Office of the European Union. <https://doi.org/10.2800/786656>

Feng, X., Ouyang, M., Liu, X., Lu, L., Xia, Y. and He, X., 2018. Thermal runaway mechanism of lithium-ion battery for electric vehicles: A review. Energy Storage Materials, 10, pp.246–267. <https://doi.org/10.1016/j.ensm.2017.05.013>

Fent, K.W., Evans, D.E., Babik, K., Striley, C., Bertke, S., Kerber, S., Smith, D. and Horn, G.P., 2018. Airborne contaminants during controlled residential fires. Journal of Occupational and Environmental Hygiene, 15(5), pp.399–412. <https://doi.org/10.1080/15459624.2018.1445260>

Fine, P.M., Cass, G.R. & Simoneit, B.R. (2001) 'Chemical characterization of fine particle emissions from fireplace combustion of woods grown in the northeastern United States', Environmental Science & Technology, 35(13), pp. 2665-2675. doi: 10.1021/es001466k.

Goldstein, J.I., Newbury, D.E., Michael, J.R., Ritchie, N.W.M., Scott, J.H.J. and Joy, D.C., 2018. Scanning electron microscopy and X-ray microanalysis. 4th ed. New York: Springer. <https://doi.org/10.1007/978-1-4939-6676-9>

Goldsmith, W.T., Ranpara, A., Batchelor, T.P., Wang, J., Burns, R. and Harper, D.F., 2025. Laboratory-scale study to model wildland–urban interface combustion emissions (Report R440). Underwriters Laboratories Inc. Available at: <https://chemicalinsights.ul.org/wp-content/uploads/2025/02/R440_Lab-Scale_WUI_Emissions.pdf>

Grossetête, T., Dubourg, S., Rochard, T., Marteau, D. and Bengaouer, A., 2025. Detailed characterization of solid particles emitted during the thermal runaway of large-scale lithium-ion cells. Journal of Energy Storage, 124, 116666. <https://doi.org/10.1016/j.est.2025.116666>

Han, L., Zhang, M., Hu, X., Yang, X., Li, J., Wei, X., Han, G., Jiang, L., Deng, Y. and Cheng, Y. (2025) Multimodal synchronous monitoring platform for state of charge stratified thermal runaway in lithium iron phosphate batteries. Next Energy, 9, 100398. <https://doi.org/10.1016/j.nxener.2025.100398>

Health Effects Institute, 2013. Understanding the health effects of ambient ultrafine particles (HEI Perspectives 3). Boston, MA: HEI.

Holder, A.L., Ahmed, A., Vukovich, J.M. and Rao, V., 2023. Hazardous air pollutant emissions estimates from wildfires in the wildland–urban interface. PNAS Nexus, 2(6), pgad186. <https://doi.org/10.1093/pnasnexus/pgad186>

Holder, A.L. and Sullivan, A.P., 2024. Emissions, chemistry, and the environmental impacts of wildland fire. Environmental Science & Technology, 58(34), pp.14963–14967. <https://doi.org/10.1021/acs.est.4c07631>

IARC Working Group on the Identification of Carcinogenic Hazards to Humans, 2023. Occupational exposure as a firefighter. IARC Monographs on the Identification of Carcinogenic Hazards to Humans, vol.132. Lyon: International Agency for Research on Cancer.

International Organization for Standardization, 2020. Reaction-to-fire tests – Heat release, smoke production and mass loss rate – Part 5: Heat release rate (cone calorimeter method) for controlled atmospheres (ISO/TS 5660-5:2020). Geneva: ISO.

Islam, M.R., Welker, J., Salam, A. and Stone, E.A., 2022. Plastic burning impacts on atmospheric fine particulate matter at urban and rural sites in the USA and Bangladesh. ACS Environmental Au, 2(5), pp.397–407. <https://doi.org/10.1021/acsenvironau.1c00054>

Jackson, O.M., Voliotis, A., Bannan, T.J., O’Meara, S.P., McFiggans, G., Johnson, D. and Coe, H., 2025. Determination of the atmospheric volatility of pesticides using FIGAERO–chemical ionisation mass spectrometry. Atmospheric Chemistry and Physics, 25(12), pp.6257–6272. <https://doi.org/10.5194/acp-25-6257-2025>

Jahn, L.G., Bland, G.D., Bowers, B.B., Monroe, L.W. and Sullivan, R.C., 2021. Metallic and crustal elements in biomass-burning aerosol and ash: Prevalence, significance and similarity to soil particles. ACS Earth and Space Chemistry, 5(1), pp.136–148. <https://doi.org/10.1021/acsearthspacechem.0c00191>

Jen, C.N., Hatch, L.E., Selimovic, V., Yokelson, R.J., Weber, R., Fernandez, A.E., Kreisberg, N.M., Barsanti, K.C. and Goldstein, A.H., 2019. Speciated and total emission factors of particulate organics from burning western US wildland fuels and their dependence on combustion efficiency. Atmospheric Chemistry and Physics, 19(2), pp.1013–1026. <https://doi.org/10.5194/acp-19-1013-2019>

Jenkins, B.M., Jones, A.D., Turn, S.Q. & Williams, R.B. (1996) 'Emission factors for polycyclic aromatic hydrocarbons from biomass burning', Environmental Science & Technology, 30(8), pp. 2462-2469. doi: 10.1021/es950699m.

Jiao, A., Zhu, S., MacKinnon, M., Wu, K., Samuelsen, S., Hopfer, S. and Wu, J., 2025. Examining the health impacts of short-term repeated exposure to wildfire smoke (Report No. 21RD003). California Air Resources Board, Research Division, University of California, Irvine.

Kesler, R.M., Sauer, N., Barowy, A., Probert, C., Neumann, D.L., Mayer, A.C., Niemeier-Walsh, M., Fent, K.W., Stapleton, H.M. and Horn, G.P., 2025. Evaluation of combustion products in air from electric and internal combustion engine vehicles during full-scale fire experiments. Fire Safety Journal, 158, 104558. <https://doi.org/10.1016/j.firesaf.2025.104558>

Kirchsteiger, B., 2022. Polycyclic aromatic hydrocarbons in different environmentally important matrices (Doctoral dissertation). Technische Universität Wien.

Kim, H., Nakamura, H., Suda, T., Tamura, K., Ogawa, J., Osaka, K., Oshima, H. and Hasaka, M., 2020. Room-temperature graphitization in a solid-phase reaction. RSC Advances, 10(3), pp.1381–1387. <https://doi.org/10.1039/C9RA09038J>

Kimelman, J. (2025) The LA County fires devastated homes in the wildland urban interface. Here’s what that is. CalMatters. Available at: https://calmatters.org/environment/wildfires/2025/01/la-county-fires-wildland-urban-interface/

Kontkanen, J., Deng, C., Fu, Y., Dada, L., Zhou, Y., Cai, J., Daellenbach, K.R., Hakala, S., Kokkonen, T.V., Lin, Z., Liu, Y., Wang, Y., Yan, C., Petäjä, T., Jiang, J., Kulmala, M. and Paasonen, P., 2020. Size-resolved particle number emissions in Beijing determined from measured particle size distributions. Atmospheric Chemistry and Physics, 20(19), pp.11329–11348. <https://doi.org/10.5194/acp-20-11329-2020>

Kortelainen, M., Jokiniemi, J., Tiitta, P., Tissari, J., Lamberg, H., Leskinen, J., Grigonyte-Lopez Rodriguez, J., Koponen, H., Antikainen, S., Nuutinen, I. & Zimmermann, R. (2018) 'Time-resolved chemical composition of small-scale batch combustion emissions from various wood species', Fuel, 233, pp. 224-236. <http://dx.doi.org/10.1016/j.fuel.2018.06.056>

Kwon, H.S., Ryu, M.H. and Carlsten, C., 2020. Ultrafine particles: Unique physicochemical properties relevant to health and disease. Experimental & Molecular Medicine, 52(3), pp.318–328. <https://doi.org/10.1038/s12276-020-0405-1>

Larsson, F., Andersson, P., Blomqvist, P. and Lorén, A., 2017. Toxic fluoride gas emissions from lithium-ion battery fires. Scientific Reports, 7, 10018. <https://doi.org/10.1038/s41598-017-09784->z

Legutko, P., Stelmachowski, P., Yu, X., Zhao, Z., Sojka, Z. and Kotarba, A., 2023. Catalytic soot combustion – General concepts and alkali promotion. ACS Catalysis, 13(5), pp.3395–3418. <https://doi.org/10.1021/acscatal.2c05994>

Li, X., Wang, Z. and Guo, T., 2021. Emission of PM₂.₅-bound polycyclic aromatic hydrocarbons from biomass and coal combustion in China. Atmosphere, 12(9), 1129. <https://doi.org/10.3390/atmos12091129>

Liu, P., Liu, C., Yang, K., Zhang, M., Gao, F., Mao, B., Li, H., Duan, Q. and Wang, Q., 2020. Thermal runaway and fire behaviors of lithium iron phosphate battery induced by over heating. Journal of Energy Storage, 31, 101714. https://doi.org/10.1016/j.est.2020.101714

Luo, J., Zhu, X., Zhong, Z., Chen, G., Hong, Y. and Zhou, Z., 2024. Enhanced catalytic soot oxidation over Co-based metal oxides: Effects of transition metal doping. Molecules, 29(1), 41. <https://doi.org/10.3390/molecules29010041>

Ma, X., Zangmeister, C.D. and Zachariah, M.R., 2013. Soot oxidation kinetics: A comparison study of two tandem ion-mobility methods. Journal of Physical Chemistry C, 117(21), pp.10723–10729. <https://doi.org/10.1021/jp400477v>

Maranghides, A., Link, E. and Brown, R., 2021. A case study of a community affected by the Camp Fire: Preliminary findings on fire behavior and structural ignition (NIST Technical Note 2135). National Institute of Standards and Technology. <https://doi.org/10.6028/NIST.TN.2135>

McClure, C.D., Lim, C.Y., Hagan, D.H., Kroll, J.H. and Cappa, C.D., 2020. Biomass-burning-derived particles from a wide variety of fuels – Part 1: Properties of primary particles. Atmospheric Chemistry and Physics, 20(3), pp.1531–1556. <https://doi.org/10.5194/acp-20-1531-2020>

Meister, M., Sharma, S., He, X., Chepaitis, P.S., Waddey, T., Wilson, M., Premnath, V., Jeevarajan, J., Black, M. and Wright, C., 2025. Evaluating inhalation risks and toxicological impacts of lithium-ion battery thermal runaway emissions. Environment International, 199, 109466. <https://doi.org/10.1016/j.envint.2025.109466>

Moreno, T., Reche, C., Ahn, K.-H., Eun, H.-R., Kim, W.Y., Kim, H.-S., Fernández-Iriarte, A., Amato, F. and Querol, X., 2020. Using miniaturised scanning mobility particle sizers to observe size distribution patterns of quasi-ultrafine aerosols inhaled during city commuting. Environmental Research, 191, 109978. <https://doi.org/10.1016/j.envres.2020.109978>

Napolitano, E., Adanouj, I., Sommariva, M., Enzo, S. and Lebedeva, N. (2025) Characterisation of smoke particles from lithium-ion battery fire: morphology, size and composition. Luxembourg: Publications Office of the European Union (JRC136854). doi:10.2760/4170802

National Academies of Sciences, Engineering, and Medicine, 2022. The chemistry of fires at the wildland–urban interface. Washington, DC: The National Academies Press. <https://doi.org/10.17226/26460>

Natural Resources Canada (2024) Canada’s record-breaking wildfires in 2023: A fiery wake-up call. Available at: https://natural-resources.canada.ca/stories/simply-science/canada-s-record-breaking-wildfires-2023-fiery-wake-call

Nobili, A., Cuoci, A., Pejpichestakul, W., Pelucchi, M., Cavallotti, C. and Faravelli, T., 2022. Modeling soot particles as stable radicals: A chemical kinetic study on formation and oxidation. Part I. Soot formation in ethylene laminar premixed and counterflow diffusion flames. Combustion and Flame, 243, 112073. https://doi.org/10.1016/j.combustflame.2022.112073

Pandey, S.K., Kim, K.-H. and Brown, R.J.C., 2011. A review of techniques for the determination of polycyclic aromatic hydrocarbons in air. TrAC Trends in Analytical Chemistry, 30(11), pp.1716–1739. https://doi.org/10.1016/j.trac.2011.06.017

Pang, Y., Chen, M., Wang, Y., Chen, X., Teng, X., Kong, S., Zheng, Z. and Li, W., 2023. Morphology and fractal dimension of size-resolved soot particles emitted from combustion sources. Journal of Geophysical Research: Atmospheres, 128(6), e2022JD037711. <https://doi.org/10.1029/2022JD037711>

Portugal, J., Bedia, C., Amato, F., Juárez-Facio, A.T., Stamatiou, R., Lazou, A., Campiglio, C.E., Elihn, K. and Piña, B., 2024. Toxicity of airborne nanoparticles: Facts and challenges. Environment International, 190, 108889. <https://doi.org/10.1016/j.envint.2024.108889>

Premnath, V., Wang, Y., Wright, N., Khalek, I. and Uribe, S., 2022. Detailed characterization of particle emissions from battery fires. Aerosol Science and Technology, 56(4), pp.337–354. <https://doi.org/10.1080/02786826.2021.2018399>

Pudikov, D.A., Zhizhin, E.V., Rybkin, A.G. and Shikin, A.M., 2018. Graphene fabrication via carbon segregation through transition metal films. Thin Solid Films, 648, pp.120–127. https://doi.org/10.1016/j.tsf.2018.01.009

Rappold, A.G., Cascio, W.E., Kilaru, V.J., Stone, S.L., Neas, L.M., Devlin, R.B. and Diaz-Sanchez, D., 2012. Cardio-respiratory outcomes associated with exposure to wildfire smoke are modified by measures of community health. Environmental Health, 11, 71. https://doi.org/10.1186/1476-069X-11-71

Reid, C.E., et al., 2016. Critical review of health impacts of wildfire smoke exposure. Environmental Health Perspectives, 124(9), pp.1334–1343. <https://doi.org/10.1289/ehp.1409277>

Riss, C.S., Faulstich, S.D., Reuther, P.S., Metcalf, W.J., Darrow, L.A., Holmes, H.A. and Strickland, M.J. (2025) 'Influence of fire characteristics on the associations between smoke PM2.5 exposure and acute cardiorespiratory health events', Environment International. doi: 10.1016/j.envint.2025.109577.

Rumi, M.J.U., et al., 2025. Invisible hazards of ultrafine particles (UFPs) from Wildland–Urban Interface (WUI) fire smoke as emerging public health risks: A critical review of transformation dynamics from emission sources, exposure pathways and vulnerable population to WUI fire toxicants. Science of the Total Environment. Advance online publication. <https://doi.org/10.1016/j.scitotenv.2025.180656>

Shrivastava, M., Fan, J., Zhang, Y., Rasool, Q., Zhao, B., Shen, J., Pierce, J.R., Jathar, S.H., Akherati, A., Zhang, J., Zaveri, R.A., Gaudet, B., Liu, Y., Andreae, M.O., Pöhlker, M.L., Donahue, N.M., Wang, Y. and Seinfeld, J.H., 2024. Intense formation of secondary ultrafine particles from Amazonian vegetation fires and their invigoration of deep clouds and precipitation. One Earth, 7(6), pp.1029–1043. <https://doi.org/10.1016/j.oneear.2024.05.015>

Su, W.-C., Lee, J., Afshar, M., Zhang, K. and Han, I., 2024. Assessing community health risks from exposure to ultrafine particles containing transition metals in the Greater Houston area. Science of the Total Environment, 912, 169067. <https://doi.org/10.1016/j.scitotenv.2023.169067>

Teixeira, J., Sousa, G., Azevedo, R., Almeida, A., Delerue-Matos, C., Wang, X., Santos-Silva, A., Rodrigues, F. and Oliveira, M., 2024. Characterization of wildland firefighters’ exposure to coarse, fine and ultrafine particles, polycyclic aromatic hydrocarbons and metal(loid)s and estimation of associated health risks. Toxics, 12(6), 422. <https://doi.org/10.3390/toxics12060422>

Texas A&M Engineering Extension Service, 2024. Lithium-ion battery fires and emissions characterization report. College Station, TX: Texas A&M Engineering Extension Service.

Tian, Y., Zhan, X., Zhang, Y., Qiao, Z., Lu, Y., Xia, Q., Lu, J., Zhang, X. and Chen, Z., 2025. Thermal decomposition mechanism of PF₅ and POF₃ with carbonate-based electrolytes during lithium-ion batteries’ thermal runaway. Fire, 8(9), 370. <https://doi.org/10.3390/fire8090370>

Töpperwien, J., Vignat, G., Feinberg, A.J., Daube, C., Alton, M.W., Fortner, E.C., Canagaratna, M.R., Kling, M.F., Johnson, M., Nadeau, K., Herndon, S., Jayne, J.T. and Ihme, M., 2025. Burn parameters affect PAH emissions at conditions relevant for prescribed fires. Atmospheric Pollution Research, 16(5), 102438. Available at: <https://doi.org/10.1016/j.apr.2025.102438>

United Nations Environment Programme, 2022. Spreading like wildfire: The rising threat of extraordinary landscape fires. Nairobi: UNEP.

U.S. Environmental Protection Agency (U.S. EPA), 2024. National ambient air quality standards (NAAQS) for PM. Available at: <https://www.epa.gov/pm-pollution/national-ambient-air-quality-standards-naaqs-pm>

US Fire Administration (2025) Phase 3 report on Lahaina fire released. Available at: https://www.usfa.fema.gov/blog/phase-3-report-on-lahaina-fire-released/ (Accessed: 9 December 2025).

Vicente, E.D. and Alves, C.A., 2018. An overview of particulate emissions from residential biomass combustion. Atmospheric Research, 199, pp.159–185. <https://doi.org/10.1016/j.atmosres.2017.08.027>

Wang, Y., Yu, T., Chen, J., Gao, B., Yu, M. and Zhu, J. (2025) Advances in safety of lithium-ion batteries for energy storage: Hazard characteristics and active suppression techniques. Energy Reviews, 4(1), 100117. <https://doi.org/10.1016/j.enrev.2024.100117>

Wang, H., Wang, Q., Jin, C., Xu, C., Zhao, Y., Li, Y., Zhong, C. and Feng, X., 2023. Detailed characterization of particle emissions due to thermal failure of batteries with different cathodes. Journal of Hazardous Materials, 458, 131646. <https://doi.org/10.1016/j.jhazmat.2023.131646>

Wang, Q., Mao, B., Stoliarov, S.I. and Sun, J., 2019. A review of lithium ion battery failure mechanisms and fire prevention strategies. Progress in Energy and Combustion Science, 73, pp.95–131. <https://doi.org/10.1016/j.pecs.2019.03.002>

Wei, Y., Castro, E., Yin, K., Shetin, A., Vu, B.N., Yazdi, M.D., Li, L., Liu, Y., Peralta, A.A. and Schwartz, J.D. (2025) 'Medium-term exposure to wildfire smoke PM2.5 and cardiorespiratory hospitalization risks', Epidemiology, 36(5). doi: 10.1097/EDE.0000000000001881.

Willstrand, O., Quant, M. and Hynynen, J., 2025. Contaminations from lithium-ion battery fires – Per- and polyfluoroalkyl substances (PFAS) in soot. Fire Technology, 61, pp.2889–2899. <https://doi.org/10.1007/s10694-025-01708-y>

World Health Organization, 2021. WHO global air quality guidelines: Particulate matter (PM₂.₅ and PM₁₀), ozone, nitrogen dioxide, sulfur dioxide and carbon monoxide. Geneva: WHO.

Wu, C., Liu, B., Wu, D., Yang, S., Li, X., Liu, J., Wu, Y., Mao, J., Zhang, Y., Wang, Y. and Wu, Z., 2025. Distinct bimodal size distribution in number concentration and light absorption of sub-500 nm brown carbon particles. npj Climate and Atmospheric Science, 8, 120. <https://doi.org/10.1038/s41612-025-01120-x>

Xiao, Q., Guo, H., Tan, J., Wang, Z., Xie, Y., Jin, H., Yang, M., Wang, X., Cheng, C., Huang, B. and Li, M., 2025. The significant impact of biomass-burning-emitted particles on typical haze pollution in Changsha, China. Toxics, 13(8), 691. <https://doi.org/10.3390/toxics13080691>

Yan, H. and Ezekoye, O.A., 2023. State of charge effects on active material elemental composition changes between pre-thermal-runaway and post-failure states for 8-1-1 nickel–manganese–cobalt 18650 cells. Journal of Energy Storage, 63, 106974. <https://doi.org/10.1016/j.est.2023.106974>

Yan, H., Marr, K.C. and Ezekoye, O.A. (2021) 'Towards fire forensic characteristics of failed cylindrical format lithium–ion cells and batteries', Fire Technology, 57, pp. 1723–1752. doi:10.1007/s10694-020-01079-6

Yan, C., Wu, X., Yuan, Y., Xie, Y., Wang, J., Gao, G. and Fan, Y., 2024. Experimental data simulating lithium battery charging and discharging tests under different external constraint pressure conditions. Data in Brief, 55, 110616. <https://doi.org/10.1016/j.dib.2024.110616>

Younis, M.T., Alzehery, F.O., Moussa, J., et al., 2025. A review of polycyclic aromatic hydrocarbon-induced microbial deterioration of Mediterranean heritage and conservation strategies. npj Heritage Science, 13, 414. https://doi.org/10.1038/s40494-025-01833-5

Zhang, H., et al., 2025. Long-range PM₂.₅ pollution and health impacts from the 2023 Canadian wildfires. Nature, 629, pp.798–804. <https://doi.org/10.1038/s41586-025-09482-1>

Zhang, X., Ye, X., Sun, Q., Liu, Q., Deng, F., Gu, W., Chen, Y., Wang, Y., Zhan, X., Jin, Y., Zhang, R. & Dai, Y. (2025) 'Respiratory Exposure to Lithium Nickel Manganese Cobalt Oxide Particles: Evidence for Toxicity and Disruption of Metal Homeostasis in Mice', Environment & Health, XXXX(XXX), pp. XXX-XXX. doi: 10.1021/envhealth.5c00419.

Zhang, X., Zhang, Y., Zhang, S., Yao, L. & Hao, Y. (2025) 'Lignocellulosic biomass pyrolysis: A review on the pretreatment and catalysts', Fuel Processing Technology, 279, 108352. doi: 10.1016/j.fuproc.2025.108352

Zhang, Y., Xu, R., Huang, W., Ye, T., Yu, P., Yu, W., Wu, Y., Liu, Y., Yang, Z., Wen, B., Ju, K., Song, J., Abramson, M.J., Johnson, A., Capon, A., Jalaludin, B., Green, D., Lavigne, E., Johnston, F.H., Morgan, G.G., Knibbs, L.D., Zhang, Y., Marks, G., Heyworth, J., Arblaster, J., Guo, Y.L., Morawska, L., Coelho, M.S.Z.S., Saldiva, P.H.N., Matus, P., Bi, P., Hales, S., Hu, W., Phung, D., Guo, Y. and Li, S. (2025) 'Respiratory risks from wildfire-specific PM2.5 across multiple countries and territories', Nature Sustainability, 8(5). doi: 10.1038/s41893-025-01533-9.

Zuhra, Z., Li, S., Xie, G. and Wang, X., 2023. Soot erased: Catalysts and their mechanistic chemistry. Molecules, 28(19), 6884. <https://doi.org/10.3390/molecules28196884>
