# Supplementary material for: Wildland–urban interface co-combustion of biomass, synthetic polymeric materials, and lithium-ion batteries generates a new class of ultrafine soot–metal–PAH hybrid particles
Source: Front Public Health. 2026 Mar 3;14:1768652. doi: 10.3389/fpubh.2026.1768652 (PMC12992334; doi:10.3389/fpubh.2026.1768652)

**Supplemental information**

**For**

**Wildland–Urban Interface Co-Combustion of Biomass, Synthetic Polymeric Materials, and Lithium-Ion Batteries Generates a New Class of Ultrafine Soot–Metal–PAH Hybrid Particles**

Md Jalal Uddin Rumi¹, Yulin Wu², Md. Jakir Hossain¹, Mazyar Etemadzadeh², Mengying Zhang², Todd A. Kingston¹, Rui Li², and Guowen Song²*

¹Department of Mechanical Engineering, Iowa State University, Ames, IA, United States
²Department of Apparel, Events, and Hospitality Management, Iowa State University, Ames, IA, United States

* Correspondence: Guowen Song

gwsong@iastate.edu

**Summary of contents**

**Section 2. Materials and methods**

S.2.1 Elemental composition and physical properties of pine and polystyrene materials

S.2.2 Detailed methodology for particle collection using cascade impactor

S.2.3 Gas chromatography-tandem mass spectrometry (GC-MS/MS) acquisition method for polycyclic aromatic hydrocarbons (PAHs)

S.2.3.1 Sample collection and preparation

S 2.3.2 Instrumental analysis

S 2.3.3 Peak identification and quantification

S.2.3.4 Quality assurance and control

**Tables:**

Table S.2.1 Elemental composition of pine and polystyrene materials

Table S.2.2 DLPI+ impactor stage cut points (D50) for particle size classification

Table S.2.3 Calibration curve parameters and limits of quantification (LOQ)

Table S.2.4 EPA quality criteria for relative response factors (RRF) and percentile (%) relative standard deviations (RSD)

**Section 3. Results and discussion**

**Tables:**

Table S.3.1 Particle mass (PM) and standard deviation (SD) across aerodynamic diameter of four fire scenarios in ultrafine-fine-coarse ranges

Table S.3.2 Particle number concentration (PNC) and standard deviation (SD) across aerodynamic diameter of four fire scenarios in ultrafine-fine-coarse ranges

Table S.3.3 Trace element concentration (ng/m^3^) in four fire scenarios across ultrafine-fine-coarse ranges

Table S.3.4 Standard deviation of trace element concentration (ng/m^3^) in four fire scenarios across ultrafine-fine-coarse ranges

Table S.3.5 Trace elements concentration percentile (%) contribution of four fire scenarios across individual ultrafine-fine-coarse ranges

Table S.3.6 EPA 16 PAH concentration (ng/m^3^) of four fire scenarios across individual ultrafine-fine-coarse ranges

Table S.3.7 EPA 16 PAH percentile (%) contribution of four fire scenarios across individual ultrafine-fine-coarse ranges

**Figures:**

Figure S.3.1 Blank quartz filter at 500× magnification. (a) SEM image, (b) EDS spectrum

Figure S.3.2 Particulates (area 1) from Pine+LIB fire in quartz filter at 1500× magnification. (a) SEM image, (b) EDS elemental mapping

Figure S.3.3 Particulates (zoom in on area 1) from Pine+LIB fire in quartz filter at 5000× magnification. (a) SEM image, (b) EDS elemental mapping

Figure S.3.4 Particulates (area 2) from Pine+LIB fire in quartz filter at 5000× magnification. (a) SEM image, (b) EDS elemental mapping

Figure S.3.5 Particulates (area 3) from Pine+LIB fire in quartz filter at 1500× magnification. (a) SEM image, (b) EDS elemental mapping

**Section 2. Materials and methods**

**S.2.1 Elemental composition and physical properties of pine and polystyrene materials**

To reflect the defining combustion in wildland-urban interface (WUI) fires, where vegetation, synthetic materials, and embedded lithium-ion batteries (LIB) burn concurrently, this study selected pine as pure biomass, a representative wildland fuel, and polystyrene (PS) as a starting proxy for common synthetic materials prevalent in North American residential settings. Pine and PS were processed into 100 mm diameter discs at the specified weight percentages (wt.%) in manuscript Table 1 to ensure consistent combustion of the fuel package. Elemental compositions of these materials, determined by using a CHNS/O elemental analyzer (FlashSmart^TM^), are detailed below in Table S.2.1.

**Table S.2.1 Elemental composition of pine and polystyrene materials**

| **Material** | **Elemental composition (%)** | | | | |
| --- | --- | --- | --- | --- | --- |
|  | **C** | **H** | **O** | **N** | **S** |
| Pine | 48.80 | 6.08 | 26.31 | 0.02 | NA |
| PS | 90.57 | 7.78 | NA | NA | NA |

**S.2.2 Detailed methodology for particle collection using cascade impactor**

The Dekati Low Pressure Impactor (DLPI+) was used to measure the mass size distribution of particulate emissions from various fire scenarios, as listed in Table 1. This 14-stage DLPI+ classifies particles into 14 size fractions ranging from 0.016 to 10 µm. The DLPI+ operates at a nominal sample flow rate of 10 liters per min (lpm) and maintains a low pressure of 40 mbar, integrated within the system to eliminate the need for external flow control. The experimental setup, as depicted in Fig. 1, positioned the DLPI+ downstream of a lab-based combustion chamber equipped with a smoke tunnel, where controlled thermal abuse conditions (uniform radiative heat flux of 50 kW/m² with normoxic atmospheric conditions of 20.95% O₂) were applied to all fuel package controlled thermal abuse conditions to replicate different fire scenarios.

Prior to sampling, 0.1 μm polycarbonate track-etched (PCTE) membrane filters (25 mm diameter) were pre-weighed using a high-precision microbalance (Model UMT2) in a temperature- and humidity-controlled environment (22 ± 1°C, 45 ± 5% RH) to ensure measurement accuracy. The DLPI+ sample flow rate was verified before each experiment using a calibrated flow meter to confirm a consistent 10 lpm. During sampling, particles from the combustion chamber were drawn through the impactor, where they were aerodynamically classified based on their inertial properties and collected onto substrates corresponding to the D50 cut points of each stage (Table S.2.2). The cut points, provided by the manufacturer, ensure precise size fractionation across the 14 stages.

After sampling, the substrates were carefully removed using clean forceps and reweighed on the microbalance to determine the mass of collected particles per stage. The mass size distribution was calculated by subtracting the pre-weighed filter mass from the post-weighed mass for each stage. Collected particles were stored in airtight containers at 4°C to preserve their integrity for subsequent analyses with gas chromatography-mass spectrometry (GC-MS) and inductively coupled plasma mass spectrometry (ICP-MS).

**Table S.2.2 DLPI+ impactor stage cut points (D50) for particle size classification**

| **Stage** | **2** | **3** | **4** | **5** | **6** | **7** | **8** | **9** | **10** | **11** | **12** | **13** | **14** | **15** |
| --- | --- | --- | --- | --- | --- | --- | --- | --- | --- | --- | --- | --- | --- | --- |
| Size (μm) | 0.016 | 0.030 | 0.054 | 0.094 | 0.15 | 0.25 | 0.38 | 0.6 | 0.94 | 1.6 | 2.5 | 3.6 | 5.3 | 10 |

**S.2.3 Gas chromatography-tandem mass spectrometry (GC-MS/MS) acquisition method for polycyclic aromatic hydrocarbons (PAHs)**

#### **S.2.3.1 Sample collection and preparation**

#### Polycyclic aromatic hydrocarbons (PAHs) were collected using XAD-2 resin tubes (polyurethane foam [PUF]-XAD-2-PUF configuration) paired with 0.1 μm PCTE membrane filters to capture both gas- and particle-phase fractions, in accordance with U.S. Environmental Protection Agency (EPA) Method TO-13A. Size-segregated particle-bound PAHs were obtained using a 14-stage DLPI+, allowing for detailed mass distribution analysis across particle sizes ranging from 0.016 to 10 µm. Preparation of the 16 U.S. EPA priority PAHs was conducted according to established EPA protocols. Extraction was conducted via accelerated solvent extraction (Dionex ASE 350) for 20 min. The extract was filtered through a 0.2 μm PTFE syringe filter and concentrated to a final volume of 1 mL using a nitrogen evaporator (N-EVAP).

#### **S.2.3.2 Instrumental analysis**

#### PAH quantification was performed using GC-MS/MS with an Agilent 7250 Accurate-Mass Quadrupole Time-of-Flight (Q-TOF) system, hosted at the W.M. Keck Metabolomics Research Laboratory, Iowa State University. Chromatographic separation was achieved on a DB-5MS column (30 m × 250 μm × 0.25 μm). The temperature protocol commenced at 50 °C for 2 min, increased to 150 °C at 25 °C/min (held for 3 min), to 165 °C at 5 °C/min (held for 3 min), to 175 °C at 10 °C/min (held for 5 min), to 225 °C at 25°C/min (held for 5 min), to 265 °C at 20 °C/min (held for 10 min), to 300 °C at 5 °C/min, and finally to 320 °C at 10 °C/min (held for 5 min). Helium was used as the carrier gas at a constant flow rate of 1 mL/min. Samples were injected in splitless mode with a volume of 2 μL. The GC-MS/MS operated in electron impact ionization mode at 70 eV, with ion source, injector, and transfer line temperatures set at 340 °C, 300 °C, and 280 °C, respectively.

#### **S.2.3.3 Peak identification and quantification**

#### Peak identification was confirmed by comparing retention times from extracted-ion chromatograms (EIC) with external standards and matching fragment ions to the NIST mass spectral database for the 16 PAHs. Quantification relied on a 16-PAH calibration mix (610 PAH Mix A) and an internal standard mix (EPA 8270 Semi-Volatile Internal Standard Mix). Data analysis was conducted using Agilent MassHunter Quantitative Analysis software.

#### **S.2.3.4 Quality assurance and control**

Analytical reliability was evaluated through the limits of detection (LOD) and limits of quantification (LOQ), and linearity was assessed via a 9-point calibration curve (0.025–10 μg/mL), with R² values exceeding 0.99 for all target analytes (Table S.2.3). Relative response factors (RRF) and relative standard deviations (%RSD) complied with EPA quality criteria (Table S2.4), though elevated RRF values were noted, potentially due to detector saturation in the new instrument under high analyte concentrations. Procedural and field blanks, spiked with a combination semi-volatile surrogate standard (SPEX CertiPrep™), showed no detectable PAHs, with recovery rates ranging from 60% to 120%. Five deuterated internal standards (acenaphthene-d10, chrysene-d12, naphthalene-d8, perylene-d12, phenanthrene-d10; EPA 8270 Mix) were employed to mitigate instrumental variability. Sample preparation used glassware to prevent cross-contamination, with analytical-grade dichloromethane (DCM) used for extraction and washing.

**Table S.2.3 Calibration curve parameters and limits of quantification (LOQ)**

| **PAH** | **RT (min)** | **Quantitation ion** | **R^2^** | **LOQ (µg/mL)** |
| --- | --- | --- | --- | --- |
| **Naphthalene** | 7 | 128 | 0.99 | 0.13 |
| **NaP-d8** | 7 | 136 | NA | ND |
| **Acenaphthylene** | 10 | 152 | 0.99 | 0.09 |
| **Acenaphthene** | 11 | 153 | 0.99 | 0.13 |
| **AcP-d10** | 11 | 164 | NA | ND |
| **Fluorene** | 13 | 166 | 0.99 | 0.10 |
| **Phenanthrene** | 18 | 178 | 0.99 | 0.04 |
| **Phe-d10** | 18 | 188 | NA | ND |
| **Anthracene** | 18 | 178 | 0.99 | 0.09 |
| **Fluoranthene** | 24 | 202 | 0.99 | 0.12 |
| **Pyrene** | 25 | 202 | 0.99 | 0.09 |
| **Benzo[a]anthracene** | 30 | 228 | 0.99 | 0.05 |
| **Chrysene** | 30 | 228 | 0.99 | 0.07 |
| **Chr-d12** | 30 | 240 | NA | ND |
| **Benzo[b]fluoranthene** | 34 | 252 | 0.99 | 0.08 |
| **Benzo[k]fluoranthene** | 34 | 252 | 0.99 | 0.08 |
| **Benzo[a]pyrene** | 35.82 | 252 | 0.9995 | 0.07 |
| **Perylene-d12** | 36.16 | 264 | NA | ND |
| **Indeno[1,2,3-cd]pyrene** | 42.79 | 276 | 0.9980 | 0.1 |
| **Dibenzo[a,h]anthracene** | 43.14 | 278 | 0.9996 | 0.1 |
| **Benzo[ghi]perylene** | 44.13 | 276 | 0.9998 | 0.06 |

**Table S.2.4 EPA quality criteria for relative response factors (RRF) and percentile (%) relative standard deviations (RSD)**

| **Compound** | **RRF minimum** | **RRF_ave_** | **%RSD** |
| --- | --- | --- | --- |
| **Naphthalene** | 0.7 | 4 | 11 |
| **Acenaphthylene** | 1.3 | 5 | 17 |
| **Acenaphthene** | 0.8 | 3 | 18 |
| **Fluorene** | 0.9 | 4 | 18 |
| **Phenanthrene** | 0.7 | 3 | 19 |
| **Anthracene** | 0.7 | 2 | 19 |
| **Fluoranthene** | 0.6 | 3 | 14 |
| **Pyrene** | 0.6 | 8 | 18 |
| **Benzo(a)anthracene** | 0.8 | 5 | 16 |
| **Chrysene** | 0.7 | 2 | 19 |
| **Benzo(b)fluoranthene** | 0.7 | 4 | 10 |
| **Benzo(k)fluoranthene** | 0.7 | 4 | 17 |
| **Benzo(a)pyrene** | 0.7 | 4 | 6 |
| **Indeno(1,2,3-cd)pyrene** | 0.5 | 2 | 18 |
| **Dibenz(a,h)anthracene** | 0.4 | 2 | 18 |
| **Benzo(g,h,i)perylene** | 0.5 | 2 | 7 |

**Supplemental Section 3. Results and discussion**

**Table S.3.1 Particle mass (PM) and standard deviation (SD) across aerodynamic diameter of four fire scenarios in ultrafine-fine-coarse ranges**

| **Particle Mass (mg)** | | | | | | | | |
| --- | --- | --- | --- | --- | --- | --- | --- | --- |
| **Aerodynamic diameter (µm)** | **Pine** | | **Pine+LIB** | | **Pine+PS** | | **Pine+PS+LIB** | |
|  | **PM** | **SD** | **PM** | **SD** | **PM** | **SD** | **PM** | **SD** |
| **0.016** | 0.02 | 0.02 | 0.01 | 0.00 | 0.01 | 0.01 | 0.01 | 0.01 |
| **0.03** | 0.03 | 0.02 | 0.05 | 0.03 | 0.02 | 0.01 | 0.02 | 0.02 |
| **0.054** | 0.21 | 0.05 | 0.11 | 0.00 | 0.14 | 0.04 | 0.14 | 0.03 |
| **0.094** | 0.37 | 0.12 | 0.30 | 0.11 | 0.69 | 0.17 | 0.59 | 0.08 |
| **0.15** | 0.32 | 0.17 | 0.29 | 0.04 | 0.78 | 0.08 | 0.68 | 0.15 |
| **0.25** | 0.17 | 0.12 | 0.33 | 0.07 | 0.86 | 0.04 | 0.66 | 0.06 |
| **0.38** | 0.09 | 0.02 | 0.29 | 0.02 | 0.43 | 0.04 | 0.41 | 0.06 |
| **0.6** | 0.06 | 0.01 | 0.16 | 0.06 | 0.33 | 0.06 | 0.27 | 0.02 |
| **0.94** | 0.04 | 0.02 | 0.11 | 0.04 | 0.16 | 0.05 | 0.20 | 0.02 |
| **1.62** | 0.03 | 0.01 | 0.19 | 0.10 | 0.12 | 0.02 | 0.24 | 0.10 |
| **2.46** | 0.02 | 0.01 | 0.22 | 0.16 | 0.09 | 0.09 | 0.26 | 0.09 |
| **3.63** | 0.02 | 0.01 | 0.14 | 0.09 | 0.06 | 0.06 | 0.15 | 0.04 |
| **5.34** | 0.01 | 0.01 | 0.05 | 0.01 | 0.06 | 0.04 | 0.05 | 0.02 |
| **10** | 0.02 | 0.02 | 0.03 | 0.04 | 0.05 | 0.02 | 0.04 | 0.03 |

**Table S.3.2 Particle number concentration (PNC) and standard deviation (SD) across aerodynamic diameter of four fire scenarios in ultrafine-fine-coarse ranges**

| **Particle number concentration (#/cm^3^)** | | | | | | | | |
| --- | --- | --- | --- | --- | --- | --- | --- | --- |
| **Aerodynamic diameter (µm)** | **Pine** | | **Pine+LIB** | | **Pine+PS** | | **Pine+PS+LIB** | |
|  | **PNC** | **SD** | **PNC** | **SD** | **PNC** | **SD** | **PNC** | **SD** |
| **0.0115** | 1.78 × 10⁷ | 4.04 × 10⁶ | 9.75 × 10⁶ | 1.92 × 10⁶ | 1.15 × 10⁷ | 6.24 × 10⁶ | 4.14 × 10⁶ | 1.99 × 10⁶ |
| **0.0154** | 2.76 × 10⁷ | 1.31 × 10⁷ | 2.68 × 10⁷ | 1.42 × 10⁷ | 1.21 × 10⁷ | 5.96 × 10⁶ | 5.06 × 10⁶ | 9.52 × 10⁵ |
| **0.0205** | 1.71 × 10⁷ | 1.03 × 10⁷ | 1.24 × 10⁷ | 3.54 × 10⁶ | 3.81 × 10⁶ | 2.73 × 10⁵ | 2.95 × 10⁶ | 1.75 × 10⁶ |
| **0.0274** | 1.55 × 10⁷ | 6.46 × 10⁶ | 1.19 × 10⁷ | 9.91 × 10⁶ | 3.08 × 10⁶ | 1.89 × 10⁶ | 4.51 × 10⁶ | 3.74 × 10⁶ |
| **0.0365** | 1.40 × 10⁷ | 4.19 × 10⁶ | 1.31 × 10⁷ | 1.17 × 10⁷ | 3.26 × 10⁶ | 3.52 × 10⁶ | 5.36 × 10⁶ | 4.23 × 10⁶ |
| **0.0487** | 1.27 × 10⁷ | 2.63 × 10⁶ | 1.36 × 10⁷ | 1.32 × 10⁷ | 3.26 × 10⁶ | 4.44 × 10⁶ | 7.62 × 10⁶ | 4.28 × 10⁶ |
| **0.0649** | 1.50 × 10⁷ | 1.91 × 10⁶ | 1.39 × 10⁷ | 1.44 × 10⁷ | 1.00 × 10⁷ | 3.27 × 10⁶ | 1.43 × 10⁷ | 4.35 × 10⁶ |
| **0.0866** | 1.79 × 10⁷ | 4.24 × 10⁶ | 1.33 × 10⁷ | 1.44 × 10⁷ | 2.09 × 10⁷ | 5.60 × 10⁵ | 2.26 × 10⁷ | 5.13 × 10⁶ |
| **0.1155** | 1.78 × 10⁷ | 3.14 × 10⁶ | 1.07 × 10⁷ | 1.18 × 10⁷ | 2.69 × 10⁷ | 1.89 × 10⁶ | 2.51 × 10⁷ | 5.98 × 10⁶ |
| **0.154** | 1.15 × 10⁷ | 1.61 × 10⁶ | 6.19 × 10⁶ | 6.29 × 10⁶ | 2.30 × 10⁷ | 2.40 × 10⁶ | 1.87 × 10⁷ | 5.57 × 10⁶ |
| **0.2054** | 3.09 × 10⁶ | 8.18 × 10⁵ | 1.96 × 10⁶ | 2.14 × 10⁶ | 1.15 × 10⁷ | 1.72 × 10⁶ | 8.31 × 10⁶ | 3.59 × 10⁶ |
| **0.2738** | 2.60 × 10⁵ | 4.51 × 10⁵ | 0 | 0 | 7.96 × 10⁵ | 6.59 × 10⁵ | 4.82 × 10⁵ | 6.55 × 10⁵ |
| **0.337** | 7.04 × 10⁴ | 1.81 × 10⁴ | 1.12 × 10⁵ | 7.36 × 10⁴ | 1.16 × 10⁶ | 1.35 × 10⁶ | 6.45 × 10⁵ | 2.28 × 10⁵ |
| **0.3652** | 4.73 × 10⁵ | 8.20 × 10⁵ | 6.91 × 10⁴ | 9.77 × 10⁴ | 0 | 0 | 0 | 0 |
| **0.4195** | 3.82 × 10³ | 1.87 × 10³ | 2.76 × 10⁴ | 2.24 × 10⁴ | 2.20 × 10⁵ | 3.11 × 10⁵ | 8.59 × 10⁴ | 3.47 × 10⁴ |
| **0.522** | 8.99 × 10² | 5.61 × 10² | 1.24 × 10⁴ | 9.09 × 10³ | 5.07 × 10⁴ | 7.17 × 10⁴ | 2.45 × 10⁴ | 8.75 × 10³ |
| **0.65** | 3.61 × 10² | 2.50 × 10² | 4.82 × 10³ | 3.79 × 10³ | 1.05 × 10⁴ | 1.49 × 10⁴ | 7.93 × 10³ | 2.48 × 10³ |
| **0.809** | 2.14 × 10² | 1.61 × 10² | 2.70 × 10³ | 1.96 × 10³ | 5.94 × 10³ | 8.39 × 10³ | 4.59 × 10³ | 1.36 × 10³ |
| **1.007** | 1.50 × 10² | 1.05 × 10² | 3.33 × 10³ | 5.71 × 10² | 6.00 × 10³ | 8.48 × 10³ | 5.07 × 10³ | 2.20 × 10³ |
| **1.254** | 6.63 × 10¹ | 5.07 × 10¹ | 1.48 × 10³ | 2.37 × 10² | 2.24 × 10³ | 3.17 × 10³ | 2.09 × 10³ | 9.84 × 10² |
| **1.5615** | 4.85 × 10¹ | 3.54 × 10¹ | 1.21 × 10³ | 5.84 × 10¹ | 1.96 × 10³ | 2.77 × 10³ | 1.77 × 10³ | 9.02 × 10² |
| **1.944** | 2.16 × 10¹ | 1.60 × 10¹ | 7.88 × 10² | 2.61 × 10² | 1.79 × 10³ | 2.53 × 10³ | 1.32 × 10³ | 6.79 × 10² |
| **2.4205** | 8.62 | 8.01 | 3.67 × 10² | 1.85 × 10² | 1.13 × 10³ | 1.59 × 10³ | 6.88 × 10² | 4.00 × 10² |
| **3.014** | 4.41 | 4.07 | 1.73 × 10² | 8.82 × 10¹ | 6.43 × 10² | 9.09 × 10² | 3.48 × 10² | 2.00 × 10² |
| **3.7525** | 2.25 | 1.94 | 9.12 × 10¹ | 5.45 × 10¹ | 4.32 × 10² | 6.11 × 10² | 2.02 × 10² | 1.29 × 10² |
| **4.672** | 1.5 | 1.29 | 4.66 × 10¹ | 2.70 × 10¹ | 2.90 × 10² | 4.10 × 10² | 1.16 × 10² | 7.65 × 10¹ |
| **5.8165** | 8.14 × 10⁻¹ | 3.53 × 10⁻¹ | 2.61 × 10¹ | 1.99 × 10¹ | 1.61 × 10² | 2.27 × 10² | 6.11 × 10¹ | 3.55 × 10¹ |
| **7.2415** | 5.71 × 10⁻¹ | 2.12 × 10⁻¹ | 1.70 × 10¹ | 1.31 × 10¹ | 1.09 × 10² | 1.54 × 10² | 3.48 × 10¹ | 1.86 × 10¹ |
| **9.016** | 3.34 × 10⁻¹ | 3.22 × 10⁻¹ | 1.16 × 10¹ | 8.29 | 6.79 × 10¹ | 9.60 × 10¹ | 1.70 × 10¹ | 1.13 × 10¹ |

**Table S.3.3 Trace element concentration (ng/m^3^) in four fire scenarios across ultrafine-fine-coarse ranges**

| **Trace element** | **Blank filter** | **Trace element concentration (ng/m^3^) after blank filter subtraction** | | | | | | | | | | | |
| --- | --- | --- | --- | --- | --- | --- | --- | --- | --- | --- | --- | --- | --- |
|  |  | **Pine** | | | **Pine+LIB** | | | **Pine+PS** | | | **Pine+PS+LIB** | | |
|  |  | **Ultrafine** | **Fine** | **Coarse** | **Ultrafine** | **Fine** | **Coarse** | **Ultrafine** | **Fine** | **Coarse** | **Ultrafine** | **Fine** | **Coarse** |
| **Li** | 0.37 | 1.83 | 2.54 | 1.16 | 39.38 | 994.28 | 103.48 | 3.27 | 2.82 | 4.89 | 34.86 | 799.88 | 58.79 |
| **Be** | 0.00 | 0.00 | 0.00 | 0.00 | 0.00 | 0.00 | 0.00 | 0.00 | 0.00 | 0.00 | 0.00 | 0.00 | 0.00 |
| **Na** | 9.07 | 23.63 | 28.05 | 18.51 | 18.29 | 8.14 | 11.25 | 18.71 | 3.57 | 1.40 | 1.72 | 6.07 | 1.16 |
| **Mg** | 0.66 | 1.00 | 1.33 | 0.81 | 0.52 | 18.21 | 7.04 | 0.62 | 0.93 | 0.71 | 0.51 | 19.11 | 5.86 |
| **Al** | 3.65 | 4.60 | 6.66 | 8.38 | 5.55 | 619.08 | 294.87 | 4.08 | 6.27 | 19.56 | 5.18 | 472.86 | 194.29 |
| **Si** | 99.88 | 0.00 | 15.32 | 0.00 | 4.33 | 126.16 | 0.00 | 13.74 | 58.18 | 0.00 | 29.70 | 95.64 | 8.19 |
| **P** | 15.03 | 56.05 | 56.66 | 56.51 | 182.81 | 617.10 | 97.28 | 57.86 | 45.35 | 66.18 | 109.29 | 643.47 | 69.76 |
| **K** | 0.00 | 10.90 | 10.52 | 2.97 | 23.68 | 9.87 | 2.11 | 15.17 | 5.38 | 2.42 | 7.56 | 9.69 | 2.53 |
| **Ca** | 9.58 | 11.63 | 15.36 | 11.29 | 12.73 | 21.64 | 17.70 | 13.48 | 9.31 | 6.44 | 9.94 | 28.19 | 13.24 |
| **Ti** | 0.06 | 0.16 | 0.22 | 0.09 | 0.12 | 8.40 | 2.89 | 0.11 | 0.22 | 0.25 | 0.04 | 5.44 | 1.47 |
| **V** | 0.00 | 0.00 | 0.00 | 0.00 | 0.00 | 0.00 | 0.00 | 0.00 | 0.00 | 0.00 | 0.00 | 0.00 | 0.04 |
| **Cr** | 1.49 | 4.39 | 8.20 | 3.29 | 4.63 | 10.00 | 4.15 | 5.94 | 8.59 | 3.32 | 3.94 | 9.21 | 3.00 |
| **Mn** | 0.06 | 0.17 | 0.25 | 0.11 | 0.13 | 0.79 | 0.32 | 0.21 | 0.22 | 0.07 | 0.09 | 0.55 | 0.18 |
| **Fe** | 6.51 | 3.10 | 3.97 | 1.78 | 4.39 | 14.35 | 6.94 | 3.71 | 3.79 | 1.71 | 3.11 | 12.01 | 5.34 |
| **Co** | 0.29 | 0.66 | 1.09 | 1.35 | 2.45 | 454.65 | 151.42 | 1.13 | 2.51 | 5.89 | 1.43 | 383.83 | 100.35 |
| **Ni** | 2.59 | 5.50 | 8.53 | 10.19 | 16.43 | 2978.97 | 1113.30 | 9.18 | 19.17 | 44.82 | 7.85 | 2592.53 | 754.59 |
| **Cu** | 0.20 | 0.27 | 0.44 | 0.37 | 0.44 | 15.24 | 8.35 | 0.83 | 0.53 | 1.19 | 0.22 | 12.66 | 4.79 |
| **Zn** | 0.49 | 3.48 | 2.84 | 1.17 | 2.76 | 2.33 | 1.52 | 4.47 | 2.17 | 0.11 | 2.42 | 4.23 | 0.95 |
| **As** | 0.00 | 0.59 | 0.57 | 0.50 | 0.62 | 0.74 | 0.63 | 0.55 | 0.56 | 0.57 | 0.64 | 0.76 | 0.61 |
| **Se** | 0.05 | 0.00 | 0.00 | 0.00 | 0.00 | 0.00 | 0.00 | 0.00 | 0.00 | 0.00 | 0.00 | 0.00 | 0.00 |
| **Rb** | 0.00 | 0.02 | 0.02 | 0.00 | 0.06 | 0.03 | 0.02 | 0.02 | 0.01 | 0.00 | 0.02 | 0.02 | 0.00 |
| **Sr** | 0.02 | 0.02 | 0.04 | 0.02 | 0.02 | 0.04 | 0.03 | 0.03 | 0.03 | 0.02 | 0.01 | 0.04 | 0.01 |
| **Nb** | 0.03 | 0.03 | 0.03 | 0.02 | 0.03 | 0.01 | 0.04 | 0.03 | 0.04 | 0.03 | 0.05 | 0.01 | 0.02 |
| **Mo** | 0.01 | 0.28 | 0.25 | 0.04 | 0.15 | 1.21 | 0.37 | 0.60 | 0.38 | 0.06 | 0.32 | 1.41 | 0.27 |
| **Pd** | 0.01 | 0.00 | 0.00 | 0.01 | 0.01 | 0.43 | 0.12 | 0.01 | 0.01 | 0.01 | 0.01 | 0.32 | 0.08 |
| **Ag** | 0.02 | 0.03 | 0.03 | 0.00 | 0.02 | 0.08 | 0.03 | 0.02 | 0.01 | 0.01 | 0.02 | 0.06 | 0.02 |
| **Cd** | 0.02 | 0.06 | 0.06 | 0.00 | 0.05 | 0.09 | 0.01 | 0.08 | 0.05 | 0.00 | 0.05 | 0.10 | 0.00 |
| **Sn** | 0.01 | 0.02 | 0.02 | 0.01 | 0.05 | 0.27 | 0.08 | 0.07 | 0.05 | 0.01 | 0.04 | 0.14 | 0.02 |
| **Sb** | 0.00 | 0.00 | 0.00 | 0.00 | 0.01 | 0.03 | 0.01 | 0.01 | 0.00 | 0.00 | 0.01 | 0.02 | 0.00 |
| **Cs** | 0.00 | 0.00 | 0.00 | 0.00 | 0.00 | 0.00 | 0.01 | 0.00 | 0.00 | 0.00 | 0.00 | 0.00 | 0.00 |
| **Ba** | 0.03 | 0.01 | 0.04 | 0.01 | 0.02 | 1.16 | 0.44 | 0.01 | 0.02 | 0.01 | 0.01 | 0.12 | 0.04 |
| **La** | 0.00 | 0.00 | 0.00 | 0.00 | 0.00 | 0.01 | 0.01 | 0.00 | 0.00 | 0.00 | 0.00 | 0.00 | 0.00 |
| **Ce** | 0.00 | 0.00 | 0.00 | 0.00 | 0.00 | 0.01 | 0.01 | 0.00 | 0.00 | 0.00 | 0.00 | 0.01 | 0.00 |
| **Pr** | 0.00 | 0.00 | 0.00 | 0.00 | 0.00 | 0.00 | 0.01 | 0.00 | 0.00 | 0.00 | 0.00 | 0.00 | 0.00 |
| **Nd** | 0.00 | 0.00 | 0.00 | 0.00 | 0.00 | 0.00 | 0.01 | 0.00 | 0.00 | 0.00 | 0.00 | 0.00 | 0.00 |
| **Sm** | 0.00 | 0.00 | 0.00 | 0.00 | 0.00 | 0.00 | 0.01 | 0.00 | 0.00 | 0.00 | 0.00 | 0.00 | 0.00 |
| **Ta** | 0.02 | 0.15 | 0.12 | 0.13 | 0.12 | 0.03 | 0.18 | 0.13 | 0.14 | 0.13 | 0.28 | 0.04 | 0.06 |
| **W** | 0.03 | 0.03 | 0.02 | 0.02 | 0.02 | 0.02 | 0.14 | 0.04 | 0.08 | 0.05 | 0.05 | 0.03 | 0.02 |
| **Pt** | 0.01 | 0.05 | 0.05 | 0.05 | 0.05 | 0.02 | 0.04 | 0.06 | 0.06 | 0.05 | 0.08 | 0.04 | 0.03 |
| **Tl** | 0.00 | 0.00 | 0.00 | 0.00 | 0.00 | 0.01 | 0.04 | 0.00 | 0.01 | 0.01 | 0.00 | 0.01 | 0.00 |
| **Pb** | 0.01 | 0.05 | 0.04 | 0.01 | 0.05 | 0.07 | 0.02 | 0.09 | 0.04 | 0.01 | 0.03 | 0.06 | 0.01 |
| **Bi** | 0.00 | 0.00 | 0.00 | 0.00 | 0.00 | 0.01 | 0.02 | 0.00 | 0.01 | 0.00 | 0.00 | 0.01 | 0.00 |
| **Th** | 0.00 | 0.00 | 0.00 | 0.00 | 0.00 | 0.00 | 0.02 | 0.00 | 0.01 | 0.00 | 0.00 | 0.01 | 0.00 |
| **Ur** | 0.00 | 0.00 | 0.00 | 0.00 | 0.00 | 0.00 | 0.01 | 0.00 | 0.00 | 0.00 | 0.00 | 0.00 | 0.00 |

**Table S.3.4 Standard deviation of trace element concentration (ng/m^3^) in four fire scenarios across ultrafine-fine-coarse ranges**

| **Trace element** | **Blank filter** | **Standard deviation of trace element concentration (ng/m^3^)** | | | | | | | | | | | |
| --- | --- | --- | --- | --- | --- | --- | --- | --- | --- | --- | --- | --- | --- |
|  |  | **Pine** | | | **Pine+LIB** | | | **Pine+PS** | | | **Pine+PS+LIB** | | |
|  |  | **Ultrafine** | **Fine** | **Coarse** | **Ultrafine** | **Fine** | **Coarse** | **Ultrafine** | **Fine** | **Coarse** | **Ultrafine** | **Fine** | **Coarse** |
| **Li** | 0.03 | 0.09 | 0.13 | 0.06 | 1.93 | 48.77 | 5.08 | 0.16 | 0.14 | 0.24 | 1.71 | 39.23 | 2.88 |
| **Be** | 0.00 | 0.04 | 0.04 | 0.04 | 0.04 | 0.04 | 0.04 | 0.04 | 0.04 | 0.04 | 0.04 | 0.04 | 0.04 |
| **Na** | 0.81 | 1.73 | 2.05 | 1.36 | 1.34 | 0.60 | 0.83 | 1.37 | 0.28 | 0.14 | 0.16 | 0.46 | 0.13 |
| **Mg** | 0.08 | 0.16 | 0.18 | 0.14 | 0.13 | 1.82 | 0.71 | 0.14 | 0.15 | 0.14 | 0.13 | 1.91 | 0.60 |
| **Al** | 0.45 | 0.45 | 0.53 | 0.60 | 0.48 | 34.90 | 16.63 | 0.43 | 0.51 | 1.16 | 0.47 | 26.66 | 10.96 |
| **Si** | 12.75 | 1.32 | 1.85 | 1.32 | 1.37 | 10.77 | 1.32 | 1.76 | 5.10 | 1.32 | 2.84 | 8.21 | 1.49 |
| **P** | 1.35 | 6.50 | 6.53 | 6.52 | 15.19 | 48.94 | 9.03 | 6.60 | 5.96 | 7.07 | 9.85 | 51.01 | 7.28 |
| **K** | 0.04 | 0.98 | 0.95 | 0.32 | 2.11 | 0.89 | 0.26 | 1.36 | 0.51 | 0.28 | 0.69 | 0.88 | 0.29 |
| **Ca** | 1.68 | 0.79 | 1.04 | 0.76 | 0.86 | 1.47 | 1.20 | 0.91 | 0.63 | 0.44 | 0.67 | 1.91 | 0.90 |
| **Ti** | 0.01 | 0.03 | 0.03 | 0.03 | 0.03 | 0.75 | 0.26 | 0.03 | 0.03 | 0.04 | 0.03 | 0.49 | 0.13 |
| **V** | 0.00 | 0.04 | 0.04 | 0.04 | 0.04 | 0.04 | 0.04 | 0.04 | 0.04 | 0.04 | 0.04 | 0.04 | 0.04 |
| **Cr** | 0.14 | 0.11 | 0.20 | 0.09 | 0.12 | 0.24 | 0.11 | 0.15 | 0.21 | 0.09 | 0.10 | 0.23 | 0.08 |
| **Mn** | 0.01 | 0.34 | 0.34 | 0.34 | 0.34 | 0.35 | 0.34 | 0.34 | 0.34 | 0.34 | 0.34 | 0.35 | 0.34 |
| **Fe** | 0.59 | 0.30 | 0.39 | 0.17 | 0.43 | 1.41 | 0.68 | 0.36 | 0.37 | 0.17 | 0.31 | 1.18 | 0.52 |
| **Co** | 0.03 | 0.04 | 0.05 | 0.06 | 0.10 | 18.11 | 6.03 | 0.06 | 0.11 | 0.24 | 0.07 | 15.29 | 4.00 |
| **Ni** | 0.22 | 0.20 | 0.30 | 0.36 | 0.58 | 105.29 | 39.35 | 0.33 | 0.68 | 1.58 | 0.28 | 91.63 | 26.67 |
| **Cu** | 0.02 | 0.10 | 0.11 | 0.11 | 0.11 | 1.42 | 0.78 | 0.13 | 0.11 | 0.15 | 0.10 | 1.18 | 0.46 |
| **Zn** | 0.05 | 0.36 | 0.29 | 0.12 | 0.28 | 0.24 | 0.16 | 0.46 | 0.22 | 0.04 | 0.25 | 0.43 | 0.10 |
| **As** | 0.00 | 0.11 | 0.10 | 0.10 | 0.11 | 0.11 | 0.11 | 0.10 | 0.10 | 0.10 | 0.11 | 0.12 | 0.11 |
| **Se** | 0.01 | 0.05 | 0.05 | 0.05 | 0.05 | 0.05 | 0.05 | 0.05 | 0.05 | 0.05 | 0.05 | 0.05 | 0.05 |
| **Rb** | 0.00 | 0.05 | 0.05 | 0.05 | 0.05 | 0.05 | 0.05 | 0.05 | 0.05 | 0.05 | 0.05 | 0.05 | 0.05 |
| **Sr** | 0.00 | 0.01 | 0.01 | 0.01 | 0.01 | 0.01 | 0.01 | 0.01 | 0.01 | 0.01 | 0.01 | 0.01 | 0.01 |
| **Nb** | 0.00 | 0.02 | 0.02 | 0.02 | 0.02 | 0.02 | 0.02 | 0.02 | 0.02 | 0.02 | 0.02 | 0.02 | 0.02 |
| **Mo** | 0.00 | 0.04 | 0.04 | 0.03 | 0.03 | 0.12 | 0.05 | 0.07 | 0.05 | 0.03 | 0.04 | 0.14 | 0.04 |
| **Pd** | 0.00 | 0.02 | 0.02 | 0.02 | 0.02 | 0.05 | 0.02 | 0.02 | 0.02 | 0.02 | 0.02 | 0.04 | 0.02 |
| **Ag** | 0.00 | 0.06 | 0.06 | 0.06 | 0.06 | 0.06 | 0.06 | 0.06 | 0.06 | 0.06 | 0.06 | 0.06 | 0.06 |
| **Cd** | 0.00 | 0.03 | 0.03 | 0.03 | 0.03 | 0.03 | 0.03 | 0.03 | 0.03 | 0.03 | 0.03 | 0.03 | 0.03 |
| **Sn** | 0.00 | 0.02 | 0.02 | 0.02 | 0.02 | 0.03 | 0.02 | 0.02 | 0.02 | 0.02 | 0.02 | 0.03 | 0.02 |
| **Sb** | 0.00 | 0.03 | 0.03 | 0.03 | 0.03 | 0.03 | 0.03 | 0.03 | 0.03 | 0.03 | 0.03 | 0.03 | 0.03 |
| **Cs** | 0.00 | 0.04 | 0.04 | 0.04 | 0.04 | 0.04 | 0.04 | 0.04 | 0.04 | 0.04 | 0.04 | 0.04 | 0.04 |
| **Ba** | 0.01 | 0.02 | 0.02 | 0.02 | 0.02 | 0.12 | 0.05 | 0.02 | 0.02 | 0.02 | 0.02 | 0.02 | 0.02 |
| **La** | 0.00 | 0.02 | 0.02 | 0.02 | 0.02 | 0.02 | 0.02 | 0.02 | 0.02 | 0.02 | 0.02 | 0.02 | 0.02 |
| **Ce** | 0.00 | 0.02 | 0.02 | 0.02 | 0.02 | 0.02 | 0.02 | 0.02 | 0.02 | 0.02 | 0.02 | 0.02 | 0.02 |
| **Pr** | 0.00 | 0.03 | 0.03 | 0.03 | 0.03 | 0.03 | 0.03 | 0.03 | 0.03 | 0.03 | 0.03 | 0.03 | 0.03 |
| **Nd** | 0.00 | 0.02 | 0.02 | 0.02 | 0.02 | 0.02 | 0.02 | 0.02 | 0.02 | 0.02 | 0.02 | 0.02 | 0.02 |
| **Sm** | 0.00 | 0.03 | 0.03 | 0.03 | 0.03 | 0.03 | 0.03 | 0.03 | 0.03 | 0.03 | 0.03 | 0.03 | 0.03 |
| **Ta** | 0.00 | 0.04 | 0.04 | 0.04 | 0.04 | 0.04 | 0.04 | 0.04 | 0.04 | 0.04 | 0.05 | 0.04 | 0.04 |
| **W** | 0.01 | 0.11 | 0.11 | 0.11 | 0.11 | 0.11 | 0.12 | 0.11 | 0.11 | 0.11 | 0.11 | 0.11 | 0.11 |
| **Pt** | 0.00 | 0.11 | 0.11 | 0.11 | 0.11 | 0.11 | 0.11 | 0.11 | 0.11 | 0.11 | 0.11 | 0.11 | 0.11 |
| **Tl** | 0.00 | 0.11 | 0.11 | 0.11 | 0.11 | 0.11 | 0.11 | 0.11 | 0.11 | 0.11 | 0.11 | 0.11 | 0.11 |
| **Pb** | 0.00 | 0.11 | 0.11 | 0.11 | 0.11 | 0.11 | 0.11 | 0.11 | 0.11 | 0.11 | 0.11 | 0.11 | 0.11 |
| **Bi** | 0.00 | 0.11 | 0.11 | 0.11 | 0.11 | 0.11 | 0.11 | 0.11 | 0.11 | 0.11 | 0.11 | 0.11 | 0.11 |
| **Th** | 0.00 | 0.11 | 0.11 | 0.11 | 0.11 | 0.11 | 0.11 | 0.11 | 0.11 | 0.11 | 0.11 | 0.11 | 0.11 |
| **Ur** | 0.00 | 0.00 | 0.00 | 0.00 | 0.00 | 0.00 | 0.00 | 0.00 | 0.00 | 0.00 | 0.00 | 0.00 | 0.00 |

**Table S.3.5 Trace elements concentration percentile (%) contribution of four fire scenarios across individual ultrafine-fine-coarse ranges**

| **Trace Element** | **Blank Filter** | **Trace elements concentration percentile (%) contribution across individual ultrafine-fine-coarse ranges** | | | | | | | | | | | |
| --- | --- | --- | --- | --- | --- | --- | --- | --- | --- | --- | --- | --- | --- |
|  |  | **Pine** | | | **Pine+LIB** | | | **Pine+PS** | | | **Pine+PS+LIB** | | |
|  |  | **Ultrafine** | **Fine** | **Coarse** | **Ultrafine** | **Fine** | **Coarse** | **Ultrafine** | **Fine** | **Coarse** | **Ultrafine** | **Fine** | **Coarse** |
| **Li** | 0.25 | 1.42 | 1.55 | 0.98 | 12.31 | 16.84 | 5.67 | 2.12 | 1.66 | 3.05 | 15.88 | 15.69 | 4.80 |
| **Be** | 0.00 | 0.00 | 0.00 | 0.00 | 0.00 | 0.00 | 0.00 | 0.00 | 0.00 | 0.00 | 0.00 | 0.00 | 0.00 |
| **Na** | 6.04 | 18.36 | 17.18 | 15.58 | 5.72 | 0.14 | 0.62 | 12.13 | 2.09 | 0.88 | 0.78 | 0.12 | 0.09 |
| **Mg** | 0.44 | 0.78 | 0.81 | 0.69 | 0.16 | 0.31 | 0.39 | 0.40 | 0.54 | 0.44 | 0.23 | 0.37 | 0.48 |
| **Al** | 2.43 | 3.58 | 4.08 | 7.05 | 1.74 | 10.49 | 16.16 | 2.65 | 3.68 | 12.23 | 2.36 | 9.27 | 15.85 |
| **Si** | 66.49 | 0.00 | 9.39 | 0.00 | 1.35 | 2.14 | 0.00 | 8.91 | 34.12 | 0.00 | 13.53 | 1.88 | 0.67 |
| **P** | 10.00 | 43.54 | 34.70 | 47.57 | 57.14 | 10.45 | 5.33 | 37.51 | 26.60 | 41.38 | 49.80 | 12.62 | 5.69 |
| **K** | 0.00 | 8.47 | 6.44 | 2.50 | 7.40 | 0.17 | 0.12 | 9.83 | 3.16 | 1.52 | 3.45 | 0.19 | 0.21 |
| **Ca** | 6.38 | 9.03 | 9.41 | 9.51 | 3.98 | 0.37 | 0.97 | 8.74 | 5.46 | 4.02 | 4.53 | 0.55 | 1.08 |
| **Ti** | 0.04 | 0.13 | 0.13 | 0.07 | 0.04 | 0.14 | 0.16 | 0.07 | 0.13 | 0.16 | 0.02 | 0.11 | 0.12 |
| **V** | 0.00 | 0.00 | 0.00 | 0.00 | 0.00 | 0.00 | 0.00 | 0.00 | 0.00 | 0.00 | 0.00 | 0.00 | 0.00 |
| **Cr** | 0.99 | 3.41 | 5.02 | 2.77 | 1.45 | 0.17 | 0.23 | 3.85 | 5.04 | 2.07 | 1.79 | 0.18 | 0.24 |
| **Mn** | 0.04 | 0.13 | 0.15 | 0.09 | 0.04 | 0.01 | 0.02 | 0.14 | 0.13 | 0.05 | 0.04 | 0.01 | 0.01 |
| **Fe** | 4.33 | 2.41 | 2.43 | 1.49 | 1.37 | 0.24 | 0.38 | 2.40 | 2.22 | 1.07 | 1.42 | 0.24 | 0.44 |
| **Co** | 0.19 | 0.51 | 0.67 | 1.14 | 0.77 | 7.70 | 8.30 | 0.73 | 1.47 | 3.68 | 0.65 | 7.53 | 8.19 |
| **Ni** | 1.73 | 4.27 | 5.22 | 8.58 | 5.13 | 50.46 | 61.01 | 5.95 | 11.24 | 28.02 | 3.58 | 50.85 | 61.56 |
| **Cu** | 0.13 | 0.21 | 0.27 | 0.31 | 0.14 | 0.26 | 0.46 | 0.54 | 0.31 | 0.74 | 0.10 | 0.25 | 0.39 |
| **Zn** | 0.33 | 2.70 | 1.74 | 0.99 | 0.86 | 0.04 | 0.08 | 2.90 | 1.27 | 0.07 | 1.10 | 0.08 | 0.08 |
| **As** | 0.00 | 0.46 | 0.35 | 0.42 | 0.19 | 0.01 | 0.03 | 0.35 | 0.33 | 0.36 | 0.29 | 0.01 | 0.05 |
| **Se** | 0.03 | 0.00 | 0.00 | 0.00 | 0.00 | 0.00 | 0.00 | 0.00 | 0.00 | 0.00 | 0.00 | 0.00 | 0.00 |
| **Rb** | 0.00 | 0.01 | 0.01 | 0.00 | 0.02 | 0.00 | 0.00 | 0.02 | 0.01 | 0.00 | 0.01 | 0.00 | 0.00 |
| **Sr** | 0.01 | 0.02 | 0.02 | 0.02 | 0.01 | 0.00 | 0.00 | 0.02 | 0.02 | 0.01 | 0.01 | 0.00 | 0.00 |
| **Nb** | 0.02 | 0.02 | 0.02 | 0.02 | 0.01 | 0.00 | 0.00 | 0.02 | 0.02 | 0.0 | 0.02 | 0.00 | 0.00 |
| **Mo** | 0.01 | 0.22 | 0.15 | 0.03 | 0.05 | 0.02 | 0.02 | 0.39 | 0.22 | 0.04 | 0.14 | 0.03 | 0.02 |
| **Pd** | 0.01 | 0.00 | 0.00 | 0.00 | 0.00 | 0.01 | 0.01 | 0.00 | 0.00 | 0.01 | 0.00 | 0.01 | 0.01 |
| **Ag** | 0.01 | 0.02 | 0.02 | 0.00 | 0.01 | 0.00 | 0.00 | 0.01 | 0.01 | 0.01 | 0.01 | 0.00 | 0.00 |
| **Cd** | 0.01 | 0.05 | 0.03 | 0.00 | 0.02 | 0.00 | 0.00 | 0.05 | 0.03 | 0.00 | 0.02 | 0.00 | 0.00 |
| **Sn** | 0.01 | 0.01 | 0.01 | 0.01 | 0.02 | 0.00 | 0.00 | 0.04 | 0.03 | 0.01 | 0.02 | 0.00 | 0.00 |
| **Sb** | 0.00 | 0.00 | 0.00 | 0.00 | 0.00 | 0.00 | 0.00 | 0.01 | 0.00 | 0.00 | 0.00 | 0.00 | 0.00 |
| **Cs** | 0.00 | 0.00 | 0.00 | 0.00 | 0.00 | 0.00 | 0.00 | 0.00 | 0.00 | 0.00 | 0.00 | 0.00 | 0.00 |
| **Ba** | 0.02 | 0.01 | 0.03 | 0.01 | 0.01 | 0.02 | 0.02 | 0.01 | 0.01 | 0.01 | 0.00 | 0.00 | 0.00 |
| **La** | 0.00 | 0.00 | 0.00 | 0.00 | 0.00 | 0.00 | 0.00 | 0.00 | 0.00 | 0.00 | 0.00 | 0.00 | 0.00 |
| **Ce** | 0.00 | 0.00 | 0.00 | 0.00 | 0.00 | 0.00 | 0.00 | 0.00 | 0.00 | 0.00 | 0.00 | 0.00 | 0.00 |
| **Pr** | 0.00 | 0.00 | 0.00 | 0.00 | 0.00 | 0.00 | 0.00 | 0.00 | 0.00 | 0.00 | 0.00 | 0.00 | 0.00 |
| **Nd** | 0.00 | 0.00 | 0.00 | 0.00 | 0.00 | 0.00 | 0.00 | 0.00 | 0.00 | 0.00 | 0.00 | 0.00 | 0.00 |
| **Sm** | 0.00 | 0.00 | 0.00 | 0.00 | 0.00 | 0.00 | 0.00 | 0.00 | 0.00 | 0.00 | 0.00 | 0.00 | 0.00 |
| **Ta** | 0.01 | 0.11 | 0.07 | 0.11 | 0.04 | 0.00 | 0.01 | 0.09 | 0.09 | 0.08 | 0.13 | 0.00 | 0.00 |
| **W** | 0.02 | 0.02 | 0.01 | 0.02 | 0.01 | 0.00 | 0.01 | 0.02 | 0.05 | 0.03 | 0.02 | 0.00 | 0.00 |
| **Pt** | 0.01 | 0.04 | 0.03 | 0.04 | 0.02 | 0.00 | 0.00 | 0.04 | 0.03 | 0.03 | 0.04 | 0.00 | 0.00 |
| **Tl** | 0.00 | 0.00 | 0.00 | 0.00 | 0.00 | 0.00 | 0.00 | 0.00 | 0.01 | 0.00 | 0.00 | 0.00 | 0.00 |
| **Pb** | 0.00 | 0.04 | 0.03 | 0.01 | 0.01 | 0.00 | 0.00 | 0.06 | 0.02 | 0.00 | 0.01 | 0.00 | 0.00 |
| **Bi** | 0.00 | 0.00 | 0.00 | 0.00 | 0.00 | 0.00 | 0.00 | 0.00 | 0.00 | 0.00 | 0.00 | 0.00 | 0.00 |
| **Th** | 0.00 | 0.00 | 0.00 | 0.00 | 0.00 | 0.00 | 0.00 | 0.00 | 0.00 | 0.00 | 0.00 | 0.00 | 0.00 |
| **Ur** | 0.00 | 0.00 | 0.00 | 0.00 | 0.00 | 0.00 | 0.00 | 0.00 | 0.00 | 0.00 | 0.00 | 0.00 | 0.00 |

**Table S.3.6 EPA 16 PAH concentration (ng/m^3^) of four fine scenarios across individual ultrafine-fine-coarse ranges**

| **PAH concentration (ng/m^3^)** | | | | | | | | | | | | |
| --- | --- | --- | --- | --- | --- | --- | --- | --- | --- | --- | --- | --- |
| **EPA 16 PAHs** | **Pine** | | | **Pine+LIB** | | | **Pine+PS** | | | **Pine+PS+LIB** | | |
|  | **Ultrafine** | **Fine** | **Coarse** | **Ultrafine** | **Fine** | **Coarse** | **Ultrafine** | **Fine** | **Coarse** | **Ultrafine** | **Fine** | **Coarse** |
| **NaP** | 0.00 | 0.00 | 0.00 | 0.83 | 0.88 | 0.47 | 1.02 | 2.26 | 1.92 | 0.49 | 0.88 | 0.31 |
| **AcPy** | 1.34 | 0.85 | 0.00 | 0.37 | 0.20 | 0.13 | 0.37 | 0.72 | 0.10 | 0.15 | 0.56 | 0.01 |
| **AcP** | 0.00 | 0.00 | 0.00 | 0.05 | 0.08 | 0.01 | 0.15 | 0.26 | 0.13 | 0.07 | 0.02 | 0.06 |
| **Flu** | 0.41 | 0.41 | 0.00 | 0.69 | 0.24 | 0.22 | 0.37 | 0.76 | 0.13 | 0.14 | 0.74 | 0.05 |
| **Ph** | 2.26 | 2.16 | 0.45 | 0.26 | 0.64 | 0.23 | 1.76 | 5.56 | 0.41 | 0.62 | 0.80 | 0.80 |
| **Ant** | 0.34 | 0.41 | 0.00 | 0.26 | 0.72 | 0.23 | 0.51 | 2.05 | 0.59 | 0.62 | 0.77 | 0.83 |
| **FL** | 1.42 | 1.49 | 0.00 | 1.09 | 2.36 | 0.29 | 1.35 | 5.68 | 0.31 | 3.50 | 2.59 | 0.44 |
| **Pyr** | 0.94 | 0.00 | 0.21 | 0.59 | 2.14 | 0.17 | 1.08 | 3.59 | 0.28 | 1.87 | 1.40 | 0.99 |
| **BaA** | 0.00 | 0.00 | 0.00 | 1.05 | 0.70 | 0.01 | 0.77 | 1.55 | 0.22 | 0.46 | 1.11 | 0.28 |
| **Chr** | 0.00 | 0.00 | 0.00 | 1.55 | 1.10 | 0.25 | 0.39 | 1.80 | 0.14 | 0.76 | 1.62 | 0.50 |
| **BbFL** | 0.00 | 0.00 | 0.00 | 1.46 | 0.83 | 0.22 | 0.53 | 1.90 | 0.21 | 0.28 | 1.34 | 0.38 |
| **BkFL** | 0.00 | 0.00 | 0.00 | 0.85 | 0.16 | 0.01 | 0.09 | 0.65 | 0.05 | 0.10 | 0.65 | 0.16 |
| **BaP** | 0.00 | 0.00 | 0.00 | 0.25 | 0.80 | 0.14 | 0.35 | 1.46 | 0.15 | 0.08 | 0.25 | 0.24 |
| **InP** | 0.00 | 0.00 | 0.00 | 0.17 | 0.40 | 0.08 | 0.21 | 0.71 | 0.06 | 0.08 | 0.49 | 0.31 |
| **DBA** | 0.00 | 0.00 | 0.00 | 0.23 | 0.05 | 0.06 | 0.13 | 0.28 | 0.08 | 0.09 | 0.82 | 0.30 |
| **BghiP** | 0.00 | 0.00 | 0.00 | 0.13 | 0.47 | 0.08 | 0.13 | 0.84 | 0.07 | 0.08 | 0.24 | 0.23 |
| **Total PAH subsection** | **6.71** | **5.32** | **0.66** | **9.85** | **11.78** | **2.60** | **9.20** | **30.08** | **4.85** | **9.36** | **14.27** | **5.88** |
| **Grand total** | **12.69** | | | **24.23** | | | **44.12** | | | **29.51** | | |

**Table S.3.7 EPA 16 PAH percentile (%) contribution of four fine scenarios across individual ultrafine-fine-coarse ranges**

| **EPA 16 PAH percentile (%) contribution across individual ultrafine-fine-coarse ranges** | | | | | | | | | | | | |
| --- | --- | --- | --- | --- | --- | --- | --- | --- | --- | --- | --- | --- |
| **EPA 16 PAHs** | **Pine** | | | **Pine+LIB** | | | **Pine+PS** | | | **Pine+PS+LIB** | | |
|  | **Ultrafine** | **Fine** | **Coarse** | **Ultrafine** | **Fine** | **Coarse** | **Ultrafine** | **Fine** | **Coarse** | **Ultrafine** | **Fine** | **Coarse** |
| **NaP** | 0.00 | 0.00 | 0.00 | 8.48 | 7.49 | 18.10 | 11.09 | 7.51 | 39.59 | 5.23 | 6.16 | 5.24 |
| **AcPy** | 19.91 | 15.91 | 0.00 | 3.75 | 1.71 | 5.13 | 3.97 | 2.39 | 1.97 | 1.58 | 3.89 | 0.22 |
| **AcP** | 0.00 | 0.00 | 0.00 | 0.54 | 0.67 | 0.41 | 1.64 | 0.85 | 2.65 | 0.71 | 0.17 | 1.01 |
| **Flu** | 6.17 | 7.78 | 0.00 | 6.98 | 2.02 | 8.64 | 4.01 | 2.52 | 2.59 | 1.46 | 5.18 | 0.88 |
| **Ph** | 33.71 | 40.52 | 68.55 | 2.69 | 5.44 | 8.74 | 19.14 | 18.48 | 8.46 | 6.64 | 5.61 | 13.67 |
| **Ant** | 5.13 | 7.71 | 0.00 | 2.69 | 6.12 | 8.69 | 5.54 | 6.83 | 12.20 | 6.59 | 5.39 | 14.12 |
| **FL** | 21.09 | 28.09 | 0.00 | 11.03 | 20.05 | 11.08 | 14.67 | 18.88 | 6.47 | 37.39 | 18.14 | 7.49 |
| **Pyr** | 14.00 | 0.00 | 31.45 | 6.02 | 18.18 | 6.51 | 11.75 | 11.92 | 5.79 | 19.95 | 9.81 | 16.88 |
| **BaA** | 0.00 | 0.00 | 0.00 | 10.69 | 5.96 | 0.41 | 8.34 | 5.17 | 4.59 | 4.86 | 7.76 | 4.72 |
| **Chr** | 0.00 | 0.00 | 0.00 | 15.70 | 9.32 | 9.61 | 4.24 | 5.98 | 2.84 | 8.08 | 11.32 | 8.43 |
| **BbFL** | 0.00 | 0.00 | 0.00 | 14.79 | 7.09 | 8.44 | 5.72 | 6.33 | 4.37 | 3.04 | 9.42 | 6.45 |
| **BkFL** | 0.00 | 0.00 | 0.00 | 8.64 | 1.33 | 0.20 | 0.96 | 2.17 | 1.06 | 1.02 | 4.56 | 2.65 |
| **BaP** | 0.00 | 0.00 | 0.00 | 2.57 | 6.79 | 5.24 | 3.84 | 4.85 | 3.06 | 0.81 | 1.73 | 4.05 |
| **InP** | 0.00 | 0.00 | 0.00 | 1.77 | 3.38 | 3.10 | 2.23 | 2.37 | 1.15 | 0.86 | 3.44 | 5.19 |
| **DBA** | 0.00 | 0.00 | 0.00 | 2.31 | 0.44 | 2.44 | 1.41 | 0.95 | 1.75 | 0.96 | 5.73 | 5.06 |
| **BghiP** | 0.00 | 0.00 | 0.00 | 1.37 | 4.01 | 3.25 | 1.44 | 2.79 | 1.47 | 0.82 | 1.69 | 3.93 |
| **Total LMW** | **65** | **72** | **69** | **25** | **23** | **50** | **45** | **39** | **67** | **22** | **26** | **35** |
| **Total HMW** | **35** | **28** | **31** | **75** | **77** | **50** | **55** | **61** | **33** | **78** | **74** | **65** |

**Figure S.3.1 Blank quartz filter at 500× magnification. (a) SEM image, (b) EDS spectrum**


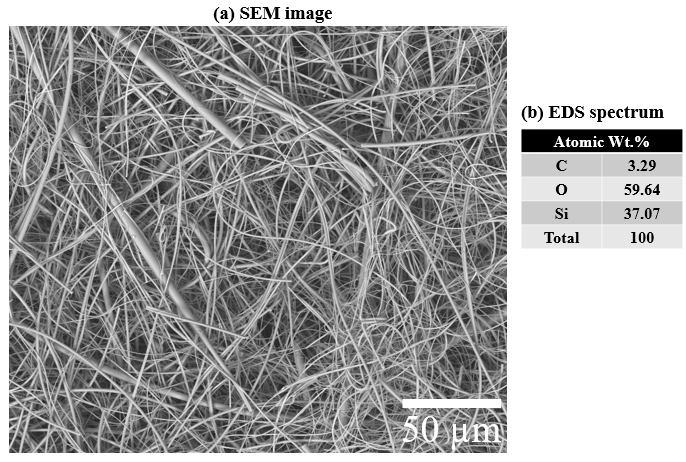


**Figure S.3.2 Particulates (area 1) from Pine+LIB fire in quartz filter at 1500× magnification. (a) SEM image, (b) EDS elemental mapping**


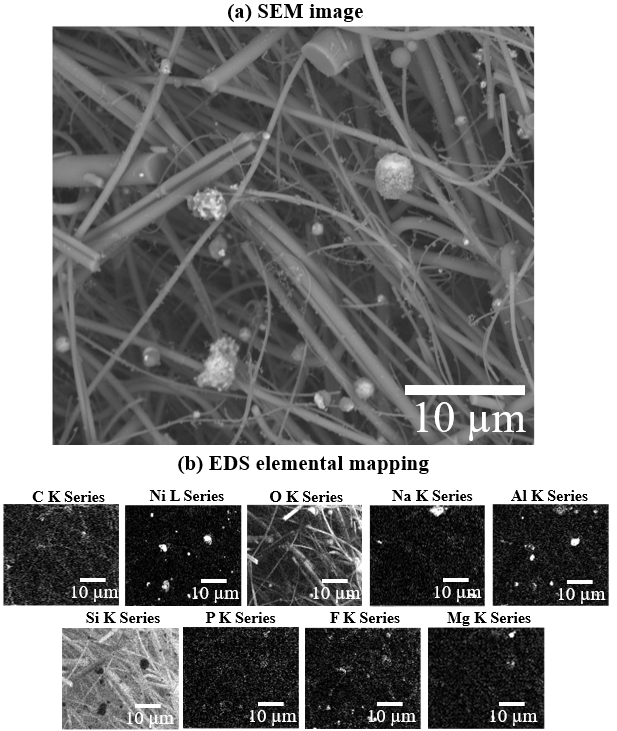


**Figure S.3.3 Particulates (zoom in on area 1) from Pine+LIB fire in quartz filter at 5000× magnification. (a) SEM image, (b) EDS elemental mapping**


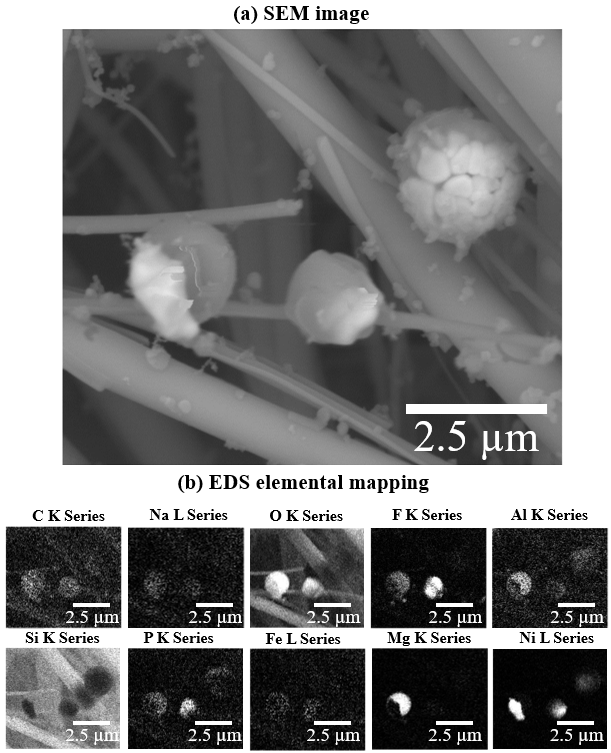


**Figure S.3.4 Particulates (area 2) from Pine+LIB fire in quartz filter at 5000× magnification. (a) SEM image, (b) EDS elemental mapping**


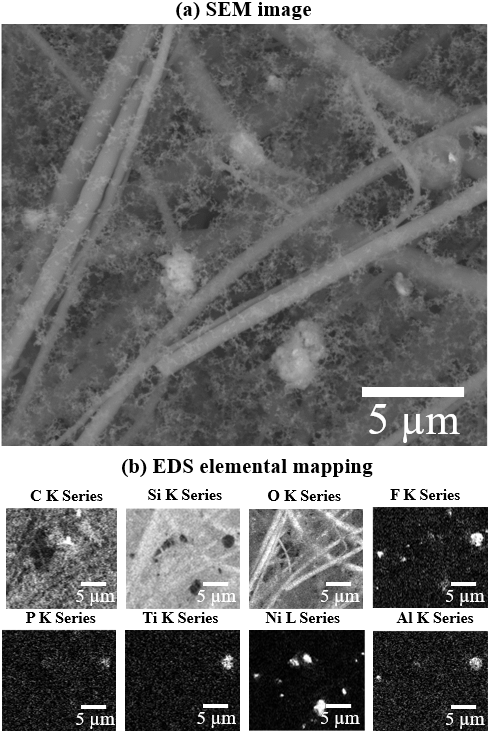


**Figure S.3.5 Particulates (area 3) from Pine+LIB fire in quartz filter at 1500× magnification. (a) SEM image, (b) EDS elemental mapping**


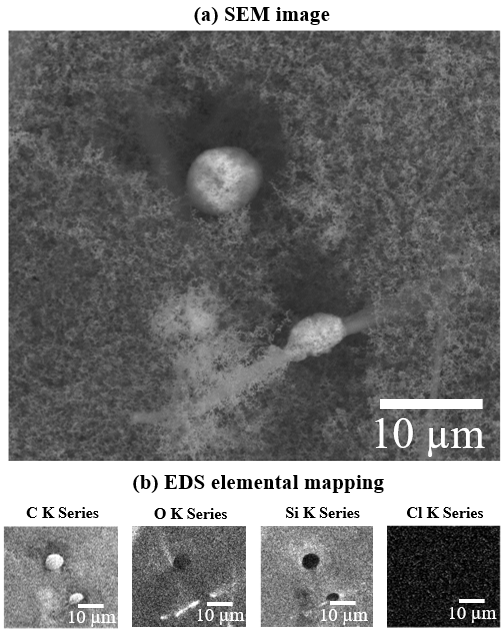

Supplement: Supplementary file 2 [file Data_Sheet_2.docx]
